# Mind your pain: A single-arm feasibility study to assess a smartphone-based interoceptive attention training for patients with chronic low back pain

**DOI:** 10.1371/journal.pone.0307690

**Published:** 2024-10-24

**Authors:** Wolf E. Mehling, Irina A. Strigo, Veronica Goldman, Wendy Hartogensis, Shelley R. Adler, Jeffrey Lotz, Frederick M. Hecht

**Affiliations:** 1 Osher Center for Integrative Health & Department of Family and Community Medicine, University of California, San Francisco, San Francisco, California, United States of America; 2 Department of Psychiatry, Weill Institute for Neurosciences, University of California, San Francisco, San Francisco, California, United States of America; 3 Emotion & Pain Laboratory, San Francisco Veterans Affairs Medical Center, San Francisco, California, United States of America; 4 Osher Center for Integrative Health, University of California, San Francisco, San Francisco, California, United States of America; 5 Department of Orthopedic Surgery, University of California, San Francisco, San Francisco, California, United States of America; 6 Osher Center for Integrative Health & Department of Medicine, University of California, San Francisco, San Francisco, California, United States of America; Universiti Sains Malaysia, MALAYSIA

## Abstract

**Objective:**

People commonly cope with chronic low back pain (cLBP) by ignoring and distraction. Can mindful interoceptive exposure to the pain sensation itself and its phenomenological components be an alternative approach?

**Methods:**

Single-arm feasibility study in patients with cLBP using a 2-minute attention exercise guided by a smartphone app several times per day over 8 weeks. We assessed feasibility, pre/post pain, function, and psychological parameters using mixed methods: standard questionnaires, ecological momentary assessment, and exit interviews that included micro-phenomenology technique and subsequent reflexive thematic qualitative analysis.

**Results:**

We enrolled 31 participants, mostly female, mean age 48, the majority had pain for >5 years; 29 completed. Mean pain intensity [0–10] improved from 4.8 ±1.7 to 3.1 ±1.9 (*p* < .0001); mean PEG scores (intensity and interference with daily life; range 0–30) improved from 13.7 ±6.2 to 8.4 ±6.6 (*p* < .0001); pain impact (9 items incl physical function) 22.3 ±8.7 to 19.7 ±8.1 (*p* = .0010). Twenty-one of 29 improved PEG score ≥30%. There were significant improvements in PCS Rumination and MAIA Not-Worrying. Participants became aware of their usual habit of avoidance and the challenge of and resistance to focusing on pain. They were surprised how pain sensations varied over time, and that pain intensity and the threat value of pain could diminish by focusing on it. They described a variety of 3D pain shapes (e.g., football, pool ball, rod, nail, brick, stars) with a range of colors, transparency, temperature, and density that for some changed with mindful attention. Most struggled to find appropriate words for sensory awareness and attention regulation and found that the threat value of their pain diminished.

**Conclusions:**

Mindful interoceptive exposure to the sensations of their cLBP using a 2-minute attention exercise with a phone app—rather than ignoring and distracting from it—may be a beneficial intervention for cLBP.

**Trial registration:**

ClinicalTrials.gov #NCT06186193.

## Introduction

Chronic low back pain (cLBP) is the number one reason for disability in the US [1] and a major burden for individuals and public health [2]. It also is a leading medical diagnosis associated with the opioid epidemic [3]. Unfortunately, there is still a lack of consensus on underlying mechanisms; discernible phenotypes; and of appropriately targeted therapies [4]. As a result, therapies generally have shown only limited benefits for these patients [4].

CLBP is generally understood as multifaceted and using a biopsychosocial approach to treatment is advocated [5]. The biopsychosocial model acknowledges psychological and social aspects of pain in addition to the biological aspects that are the more traditional focus of medicine. One open question regarding the behavioral management of chronic pain is whether there is a beneficial alternative to the common pain coping style of distraction from and ignoring the sensation. There is consensus that distraction is beneficial with acute LBP [6]. Several studies, however, have raised doubts about this coping style for chronic pain [7]. Although ignoring pain has been found to be a risk factor for the transition from acute to cLBP [8] and for the persistence of LBP [9], ignoring and distraction appear to be the most common coping style among patients with cLBP [10]. (for more comprehensive reviews see [11, 12]) The finding that ignoring pain is a risk factor for the transition to cLBP, together with current theoretical frameworks for understanding chronic pain and some experimental evidence [13, 14], suggest that the opposite coping style of ignoring pain, namely paying close attention to the sensation of pain might have beneficial effects.

This notion—that paying close attention to the sensation of pain may be beneficial—has implicitly been suggested before [15]. Mindfulness approaches are one way to train people to pay attention to body sensations, including pain, while also cultivating awareness of the mental processes that often accompany and can exacerbate pain, such as rumination (thinking repetitive, usually negative thoughts about the pain) and catastrophizing (“the pain will just get worse and worse,” “the pain will cripple me.” When the journal *Emotion* dedicated a full issue to mindfulness-based approaches for pain and negative emotions, Marc Williams, co-developer of Mindfulness-Based Cognitive Therapy (MBCT) and editor of the special issue, summarized key messages from these reports: mindfulness practice can bring about changes in the way pain and emotion are processed [16]. More specifically, students of mindfulness can learn to uncouple the immediate sensory experience from the “narrative,” the story around it, and thereby differentiate these two modes that are usually closely knit together [17]. His second conclusion was that a narrative thinking mode of attention to pain may be a more subtle way of distraction, and that one may be able to instead learn to focus on the sensory immediacy of pain itself. In other words, giving attention to pain by thinking about it may not be a productive attention style. A mode of attention that explicitly emphasizes using sensory awareness over a more cognitive attention style appears to be a key element of MBCT when coping with symptoms of depression [18, 19] and may also be helpful for coping with chronic pain [20]. A meta-analysis of 8 studies comparing distraction from pain to attention to pain found no difference for pain outcomes, but the included studies did not differentiate between these attention styles—sensory versus cognitive attention [21].

Yoga is an approach that can be used to train mindfulness, particularly related to body awareness [22, 23]. A study comparing healthy experienced yoga practitioners with yoga-naïve individuals for their attention style and potentially associated thresholds to experimental pain found that the pain threshold was increased in yogis and closely correlated with the neural density in the insula brain region, the hub for interoception, for perceptions from the inside of the body. Pain is generally an unpleasant interoceptive experience inside the body. Asked about their coping style when submerging a hand in ice water, there was a striking difference between the two groups: avoidant distraction in yoga-naïve individuals, and close sensory and curious attention style in the yogis [24] applying a form of mindful interoceptive awareness [25].

Applying a comparable attention style to chronic pain, the psychologist Bruno Cayoun developed a behavioral intervention for chronic pain based on a task extracted from his Mindfulness-integrated Cognitive Behavior Therapy program (a program distinct from MBCT): the Mindfulness-based Interoceptive Exposure Task [26, 27]. It used an individual, guided, 1-hour introduction session and a 1 to 2-minute attention task subsequently performed several times per day over 12 weeks. In a small pilot study (*N* = 15) with patients with various musculoskeletal pain problems, his team showed significant positive pre-post standardized effects (Cohen’s *d*) of 0.96 for improvements in pain anxiety, 0.86 for pain duration, and 1.37 for pain intensity, which were maintained at 2-month follow-up.

This novel behavioral task—if shown to be valid, feasible, and acceptable in populations with cLBP—implies a potential paradigm shift in the management of chronic pain. While most pain patients by default cope with pain by using distraction from pain, which can lead to rumination and worry, this approach attempts to facilitate a mindful, equipoised, ‘neutral,’ non-evaluative, curious, exploratory attention style of *immediately sensing* pain rather than *thinking* about pain.

We conducted an observational proof-of-concept study, based on the mindfulness-based interoceptive exposure intervention, to pilot test this task with a phone application (app) in patients with cLBP. The phone app can be programmed with individual notifications for brief exercises of guided sensory attention during the course of the day and can be combined with ecological momentary assessments (EMA). This could provide a low-cost scalable intervention that can be added to a more intensive mindfulness-based program or other behavioral interventions. We hypothesized that the new brief mobile phone-based mindful attention task over eight weeks would be feasible and acceptable to patients with cLBP. We explored whether this intervention would improve self-reported pain intensity and/or interference and a series of psychological parameters, such as below average interoceptive awareness. We did not prespecify hypotheses regarding the efficacy of this app-based intervention.

## Methods

### Study design

We conducted a single-arm, mixed-methods study to assess the app’s feasibility, acceptability, and potential benefits for patients with clearly defined cLBP. We applied standard pain self-report outcomes, quantitative sensory testing (QST), and task-based functional magnetic resonance imaging (fMRI). Here we report details of the study protocol and quantitative self-reported patient outcomes, as well as an analysis of qualitative exit interviews illustrating the quantitative results. Detailed QST and fMRI results will be reported separately as these would overextend this already lengthy report even further.

Following IRB approval (20–32001; S1 File), we enrolled patients from May 21^st^, 2021, until February 21^st^, 2023 with follow-up data collected until May 21, 2023. The study was registered with ClinicalTrials NCT06186193 after completion of recruitment (12/6/2023). This was due to an administrative error that was corrected upon detection. The authors confirm that all ongoing and related trials for this drug/intervention are registered. Participants were compensated for their time and incentivized by receiving 50 cents for each entry into the phone app.

### Participants

Through university newsletters, research and study websites, flyers, and social media we recruited English-speaking men and woman aged 18–65 years old with cLBP defined according to the NIH Research Task Force Recommendation on Research Standards for cLBP [28]: pain at least half the days in the past 6 months, by using 2 questions and a human figure drawing illustrating the pain region. Average pain in the last month had to be at least 3 out of 10 on the numeric rating scale (NRS). Using the Multidimensional Assessment of Interoceptive Awareness scales (MAIA-2) [29], we only included patients with a low level of interoceptive awareness and habitual distraction as the dominant pain coping style. This was defined as: a) a MAIA summary score below the population mean score of 3.41 [possible range 0–5] established in a large representative sample of primary care patients [10] and b) a MAIA Non-Distraction score below 2.91 [possible range 0–5], the mean value plus standard deviation in the same sample. These criteria were chosen (a) to test our hypothesis that the mindful attention task will be able to increase below average interoceptive awareness in cLBP patients, who are used to distracting themselves from their pain experience, and (b) to avoid a potential ceiling effect. Participants had to own a smart phone.

Potential participants who visited the study website could check their preliminary eligibility. If they agreed, they were screened over the phone by a clinical research coordinator. We excluded patients with current or history of spine infection, spine tumor, vertebral fracture, cauda equina syndrome, patients with substance abuse, significant mental health, or other medical conditions (malignancies, liver failure, renal failure, pain conditions from inflammatory diseases, malignancies, abdominal aortic aneurysm, muscle weakness from radiculopathy). Radiculopathy or sciatic pain was not excluded if the condition was stable and did not lead to significant movement restrictions or muscle weakness. Regular opioid or antidepressant prescription was not an exclusion if stable over the past three months. We excluded patients involved in a lawsuit or Worker’s Compensation claim related to their back; patients who received steroid or Botox injections near the spine in the past three months; women who were pregnant, planning to get pregnant in the next months, or were less than three months post-partum. Because all participants underwent task-based brain fMRIs, we excluded colorblind and left-handed patients and patients with other typical MRI-related exclusion criteria: any metal in the body; claustrophobia; inability to lie still for approximately 60 minutes; prior neurosurgery; older tattoos with metal dyes; non-removable jewelry, braces, or permanent dental retainers.

The discussion of the electronic consent occurred by Zoom. Final eligibility was determined after answering the MAIA. After signing electronic informed consent, participants received a copy and answered questionnaires on-line at home. All electronic consents are stored on the secured university server.

### Intervention

The Mind your Pain study intervention (MyP) consists of an individual 1-hour educational introduction session over Zoom, a six-page illustrated handout summarizing the discussed pain education (S2 File), and a 1 to 2-minute attention task subsequently performed several times per day over 8 weeks. The 8-week intervention started with the 1-hour education session. We did not include the educational material in the phone app as we did not have the resources for a thorough and effective implementation. In the last minutes of this education session, the task was downloaded on a smart phone app with notifications twice per day according to participant preferences. In addition, participants were asked to use the app whenever they perceive the pain at its worst. The app included an ecological momentary assessment (EMA) of pain intensity and interference each answered on a 0–10 NRS followed by a brief audio recording guiding the participant to attend to pain as the sensation “where it is the most intense”. The audio recording aimed to guide the participants’ attention focus into the center of “the sensation we call pain” in a detached and equanimous way and asks participants to carefully observe and explore it with curiosity in regards to five components of that sensation: feeling tone (sharp versus dull), motion (static versus movement/change), temperature (cold versus hot); density (tense/tight versus loose), and clarity of the borders of the perceived three-dimensional shape (diffuse versus clearly-defined). An optional entry field was offered for participants to add other experiential characteristics of their pain experience. This neutral sensory interoceptive attention focus aimed at minimizing the learned aversive response to pain that may prompt ruminating thoughts and the negative affect commonly intertwined with immediate sensory awareness. The app was self-scheduled by the participants to provide one notification in the morning and one in the evening and used a Qualtrics survey platform [30] to enter data directly to a secure UCSF server. We pretested a prototype of the app for iPhones and Android phones with ten cLBP patients. Brief weekly phone calls by the clinical research coordinator were provided to resolve logistic or technical issues and answer conceptual questions.

### Sample size

The primary purpose of this study was to determine feasibility and validate a novel mindfulness and interoceptive exposure-based intervention in a subtype/phenotype of cLBP patients with low interoceptive awareness to determine initial clinical utility as a basis for future research. In the study by Cayoun, et al., mentioned above, participants with a variety of pain conditions experienced a significant reduction in pain intensity with a standardized effect size of *d* = 1.37 [27]. For preliminary estimations of the effect size, we used power calculations based of the normal approximation with a 5% significance level for a 2-sided test, we determined that a sample size of 30 patients would provide adequate power (.70 –.93) to detect minimal clinically important change, defined as a 30% improvement in combined pain intensity and interference [31]. As this was a proof-of-concept study rather than an efficacy study, we primarily explored standardized effect sizes (Cohen’s *d* with 95% confidence intervals) and did not correct for multiple comparisons [32].

### Measures

All questionnaire data were collected online using the Research Electronic Data Capture (REDCap) [33], a web-based, secure application that includes components for developing and implementing online surveys for clinical research, within 1–2 weeks before the start of the intervention and within 1–2 weeks after the 8-week intervention.

To determine feasibility, we assessed: number screened per month; number enrolled per month; number of completers and retention rate; and proportion of planned pre- and post-intervention assessments completed. For acceptability we assessed reasons for dropouts; proportion of EMA assessments responded to; and qualitative interviews (see below).

We used the following exploratory outcome measures: for pain intensity and interference averages on NRS in the past week as well as EMA assessments “right now”; pain interference also by PROMIS Pain Interference; combined pain intensity and interference on the PEG scale; and Pain Impact by 9-item composite measure (Cronbach’s alpha in our sample 0.84) suggested by the NIH Chronic Low Back Pain Task Force [28]. Consistent with the current standard of LBP research [34], our primary outcome measure for the exploratory analysis was the PEG score [35]. The PEG combines a scale for pain intensity and two scales for pain interference with enjoyment in life and general activity, all three measured on a 0–10 scale for the past week. Cronbach’s alpha in our sample was 0.94. We also assessed for potential differences in PEG scores and in PEG change scores between sexes but found no differences.

We also used additional questionnaires (S3 File), including Pain Self-Efficacy by Pain Self-Efficacy Questionnaire 4-item version [36]; Pain Acceptance by Chronic Pain Acceptance Questionnaire-SF8 [37]; Impression of Change by Patient Global Impression of Change (PGIC) only at 8 weeks [38]; physical Function by PROMIS Physical Functioning SF-6b; Catastrophizing by Pain Catastrophizing Scale SF-6 (PCS) [39]; Fear-Avoidance by Fear-Avoidance Beliefs Questionnaire (FABQ-PA) [40]; Mindfulness by Five Facets Mindfulness Questionnaire (FFMQ) [41]; Interoceptive Awareness by Multidimensional Assessment of Interoceptive Awareness (MAIA) [29].

To compare the questionnaire assessments asking for pain intensity and impact in the past week with EMA data, we summarized the latter for the first 7 days after the introductory educational session and for the last 7 days before answering the post-intervention questionnaire. The timepoints for eligibility screening (pain intensity ≥3/10), for the full questionnaire battery and the introductory intervention session were sometimes 2–3 weeks apart because of scheduling issues for the fMRIs.

### Quantitative analyses

we used descriptive statistics for socio-demographic baseline variables and paired *t* tests for pre-post pain measures with normal distribution and Wilcoxon Signed Rank test for non-normal distribution according to Shapiro-Wilk tests. We defined treatment responders as participants who improved their PEG scores by at least 30% [42]. Using responder status as binary outcome, we explored baseline and change variables for predictive association by logistic regression. We explored whether changes in key outcomes were correlated with changes in psychological variables by Pearson correlations and with demographic variable by Spearman rank correlations. We used statistical software Stata17 for all quantitative analyses [43].

There was a small amount of missingness for a few survey instruments for which we did not substitute, but data for key predictors and outcome variables were complete.

#### Qualitative interviews and analyses

Following the completion of the 8-week intervention, we conducted individual exit interviews with all participants and asked open -ended questions about the experience with the phone app, the attention task, its perceived usefulness and shortcomings, new insights and potential changes in their pain managements, and ways to improve the app. If participants agreed, a second part of the interview was an in-depth inquiry into the participants’ individual perception of pain using microphenomenological interview technique [44, 45]. With this technique the interviewer guides the participant gently away from thoughts and judgements about an experience into the immediacy of sensory experience itself. The interviewer was not aware of the interviewees’ questionnaire responses. The interviews were recorded and transcribed.

We conducted reflexive thematic analysis as proposed by Virginia Braun and Victoria Clarke[46, 47]. We followed a five-phase inductive analysis process (data familiarization; open coding; generating initial themes; developing and reviewing themes; refining and naming themes) to identify and make sense of patterns of meaning across our dataset. The resulting themes were actively created through the authors’ interpretative engagement with the exit interview data from all study participants using “the researcher’s subjectivity as analytical resource and their reflexive engagement with theory, data and interpretation” [47]. The authors interpretations are situated in a phenomenological framework of understanding pain as a bio-psycho-social experience potentially modifiable by mind-body approaches that emphasize mindful interoceptive awareness.

## Results

### Participants

**Fig 1:**
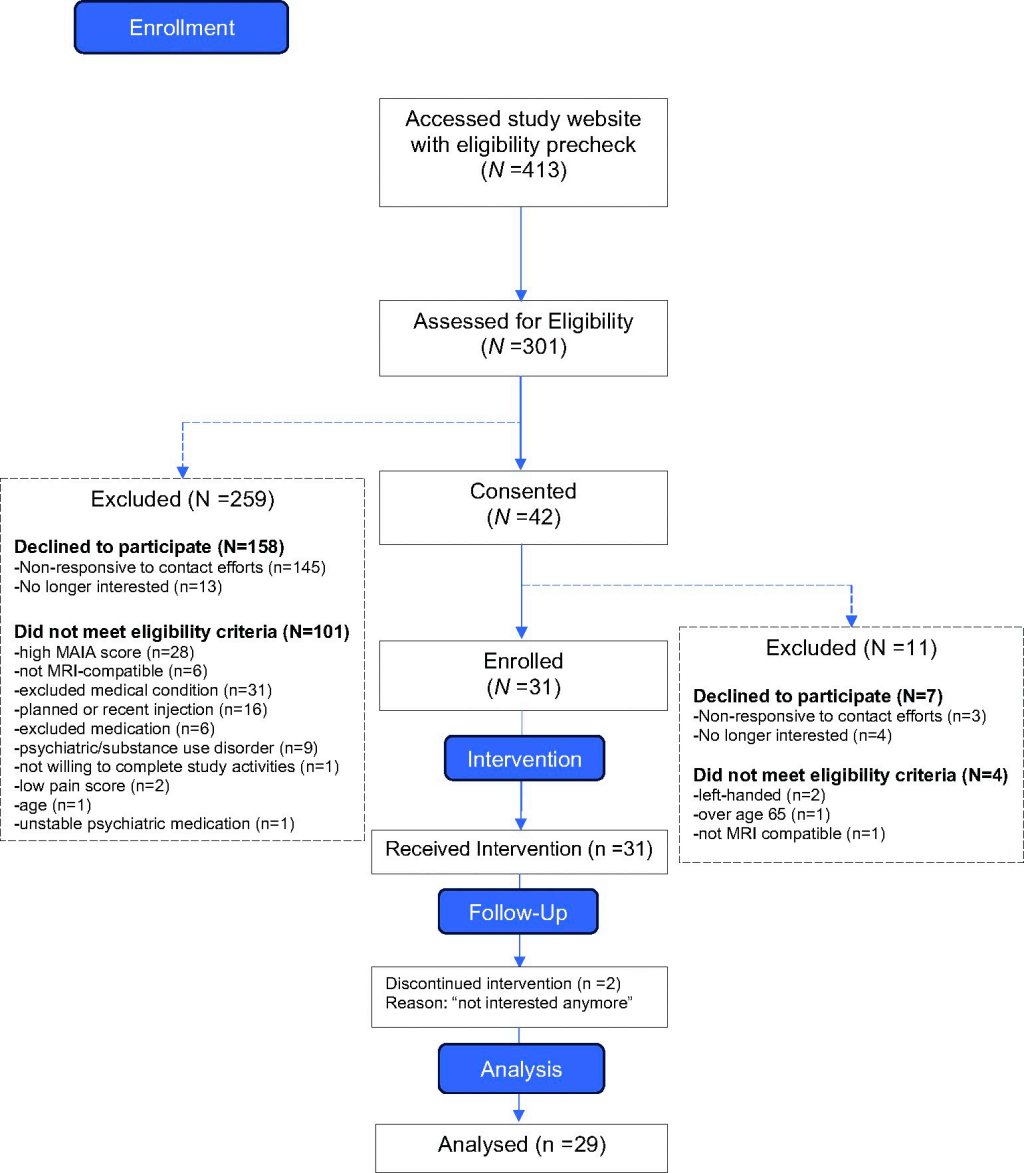
Participant flow diagram.

During 18 months of recruitment, 413 interested individuals were prechecked on our study website, of whom 31 were found to be eligible and enrolled in the study, and 29 completed study procedures (further details in Fig 1). The 29 study completers were mostly female, white, between 26 and 64 years of age with a mean age of 48.4 years (Table 1). They generally were well educated, financially well off, with LBP of moderate intensity (mean 4.8; SD = 1.7) for more than 1 year, 59% for more than 5 years. Five participants suffered from sciatica. Pain interference at baseline by PROMIS was 57.6 (SD = 8.1). Financial strain or perceived discrimination was not a major concern for our participants, but more than half of participants self-reported a history of PTSD. In line with our inclusion criterion of low interoceptive awareness by MAIA self-report, our participants scored below the general population mean on MAIA total score and habitually used a coping style of distraction from pain [10].

**Table 1:** Participant demographics and clinical characteristics at baseline (*N* = 29).

Age (years; SD)	48.4 (12.3)	[observed range 26–64]
Gender, *n*		
Male	7 (24.1%)	
Female	21 (72.4%)	
Other/nonbinary	1 (3.5%)	
Race, *n*		
White or Caucasian	21 (72.4%)	
Black or African American	1 (3.5%)	
Asian	3 (10.3%)	
American Indian or Alaska Native	0	
Native Hawaiian or Pacific Islander	0	
More Than One Race	0	
Other/Unknown/Declined	4 (13.8%)	
Hispanic or Latino, *n*	6 (20.7%)	
Unemployed, *n*	11 (37.9%)	
Married or partnered, *n*	18 (64.3%)	
Education (college degree), *n*	23 (79.3%)	
Household Income (*n* = 27)*	5 (17.2%) <$35,000 14 (48.3%) >$200,000	
High Financial Strain, *n*	1 (3.5%)	
Perceived Discrimination, *n*	3 (10.4%)	Somewhat/very often
PTSD, *n*	18 (62.1%)	9 as child, 14 as adults
PTSD Symptom Screener [0–5 (SD)]	1.17 (±1.69)	
Childhood Trauma [range 28–140]	62.2 (±7.2)	(*N* = 28, 7 missing values substituted with 1)
Expectation of pain relief in %	28.6 (±28.0)	
On antidepressants, *n*	6 (20.7%)	
Duration of pain [ordinal: 1–5]	4.5 (±0.6) 93% >1 year 59% >5 years	
Sciatica (pain below knee), *n*	5 (17.2%)	

* 2 participants did not provide information

### Feasibility/Acceptability

We prescreened about 23 potential participants per month, phone screened about 17 and enrolled about two potential participants. Recruitment was limited to individuals who were able to drive to the hospital where QST and fMRI were performed. Two of the 31 enrolled participants (6.5%) were lost to follow-up: one participant stopped using the app after one week because he preferred to be distracted from his pain; the other dropped out after 1 week without providing any explanation. We did not use their baseline data for analysis. All 29 completers provided pre-post questionnaire data and exit interviews. During the 8 weeks of the intervention, participants used the app at average 116.5 times (SD 62.8, range 32–283). Twenty-six participants used the app at least once a day and 11 at least twice a day. The app functioned without glitches, except a single participant claimed that she had used the app twice a day, but we never received any data entered into the app by her, which remained unexplained. We did not regularly check the entries into the data base and therefore did not have the opportunity to inquire into this issue during weekly phone calls.

In the qualitative exit interviews, almost all participants felt that the app was easy to use. Some participants made suggestions on how to improve the relatively simple platform and make it more attractive for future studies. Participants described initial difficulties in overcoming their habit of distracting themselves from their pain sensations (see details in qualitative results). One participant felt the app had become “a chore” (56 entries), another two reported that they “stayed in their head” particularly when pain intensity was >5/10, and another four participants “did not get into it”. For four participants who used the app less than once a day, the exit interviews provided clear statements that three of these “did not get into it” and did not feel that they learned or benefited from it. Interestingly, the number of app entries was not associated with perceived global benefits or changes in pain outcomes (*F(1*, *27)* = 0.27; *p* = 0.61).

### Quantitative outcomes

Mean pain intensity and PEG scores showed statistically significant reduction from pre to post intervention (Table 2). Eight participants reported no perceived global change and one participant felt mildly worsened, while the remainder reported perceiving minimal (*n* = 11) or much (*n* = 9) improvement. Viewing a 30% change from baseline as clinically meaningful, 18 of 29 completers had a meaningful improvement in pain intensity [31] and 21 in PEG scores for combined pain intensity and interference. Mean Pain Impact, the composite outcome measure suggested by the LBP Task Force, also improved significantly (95% CI -4.4, -0.9; *p* = .0010). Changes in Pain Impact and in PROMIS Pain Interference were highly correlated (*r* = .83; *p* < .0001). Change in pain intensity was associated with education (*ρ* = -.41; *p* = .028) and income (*ρ* = -.42; *p* = .030, indicating that participants with higher education and higher income showed greater improvement. Severity of PTSD reported at baseline was associated with responding to the intervention: the odds for the 6 individuals with the highest degree of PTSD (ordinal scale 0 to 4) to be among the responders was reduced to 0.063 (95% CI: 0.007–0.59; *p* = 0.016) compared to all other participants. Regarding the Patients’ Global Impression of Change Scale, 9 (31%) participants responded as “much improved”, 11 (37.9%) “minimally improved”, 8 (27.6%) “no change”, and 1 (3.5%) “minimally worse”.

**Table 2:** Pre-post changes in pain measures and psychological variables.

Variable [scale range]	Mean BL	SD BL	Mean FU	SD FU	Mean Diff	95% Confidence Interval		*p*		ES
Low Back Pain Intensity [0–10]	4.79	1.72	3.14	1.94	-1.66	-2.23	-1.08	**<0.0001** *		**-1.09**
PEG [0–30]	13.69	6.22	8.41	6.57	-5.28	-7.09	-3.46	**<0.0001** *		**-1.10**
Pain Impact [8–50]	22.3	8.7	19.7	8.1	-2.7	-4.4	-0.9	**0.010** *		-0.32
PROMIS Pain Interference 4a (T)	57.6	8.1	55.4	6.4	-2.2	-4.7	0.3	0.011*		-0.33
PROMIS Physical Function 6b	43.6	7.3	44.4	7.5	0.8	-0.4	2.0	0.18		0.26
PCS Total Score [0–12]	8.34	5.72	6.83	5.88	-1.52	-3.16	0.13	0.15*		-0.35
PCS Rumination [0–4]	3.00	2.07	2.17	2.04	-0.83	-1.50	-0.15	**0.018** *		**-0.47**
PCS Magnification [0–4]	2.62	2.04	2.14	2.07	-0.48	-1.11	0.15	0.17*		-0.29
PCS Helplessness [0–4]	2.72	2.20	2.52	2.21	-0.21	-0.83	0.41	0.73*		-0.13
PROMIS Anxiety4a (T)	52.9	9.6	50.7	10.1	-2.2	-5.3	0.9	0.19*		-0.28
PROMIS Depress4a (T)	51.0	8.5	49.4	10.3	-1.6	-4.4	1.2	0.32*		-0.22
PROMIS Social Role4a (T)	47.2	6.6	51.1	8.8	3.9	1.3	6.6	**0.005**		**0.58**
FABQ Score [0–24]	15.2	6.8	14.0	7.2	-1.2	-3.5	1.1	0.29		-0.21
CPAQ Total Score [0–48]	28.6	5.2	28.7	5.9	0.1	-2.0	2.2	0.947		0.01
Pain Self-Efficacy [0–24]	17.85	4.88	18.74	5.23	0.89	-0.44	2.22	0.16*		0.26
Widespread Pain [0–7]	2.00	1.73	1.66	1.61	-0.34	-0.73	0.04	0.078*		-0.34
FFMQ Acting w Awareness	17.4	4.4	16.5	4.1	-0.8	-1.9	0.3	0.13		-0.29
FFMQ Describing	18.0	3.9	17.5	3.2	-0.5	-1.7	0.7	0.39		-0.17
FFMQ Non-Judging	18.1	3.4	16.7	4.0	-1.4	-2.5	-0.4	**0.008**		**-0.54**
FFMQ Non-Reactivity	15.8	3.8	15.9	4.1	0.2	-1.0	1.4	0.77		0.06
FFMQ Observing	14.2	3.4	14.8	3.3	0.6	-0.3	1.6	0.20		0.25
MAIA Noticing [0–5]	3.28	0.73	3.04	1.07	-0.23	-0.62	0.16	0.30*		-0.23
MAIA Not-Distracting [0–5]	1.87	0.72	2.05	0.88	0.18	-0.11	0.48	0.31*	0.24	
MAIA Not-worrying [0–5]	2.81	1.06	3.19	1.10	0.39	0.10	0.67	**0.013** *	**0.52**	
MAIA Attention Regulation [0–5]	3.03	0.92	3.00	0.93	-0.03	-0.41	0.34	0.98*	-0.04	
MAIA Emotional Awareness [0–5]	2.90	1.14	2.67	1.29	-0.23	-0.65	0.18	0.26*	-0.22	
MAIA Self-Regulation [0–5]	2.77	0.86	2.77	0.85	0.00	-0.42	0.42	0.70*	0.00	
MAIA Body Listen [0–5]	1.90	1.04	1.97	1.16	0.07	-0.27	0.41	0.89*	0.08	
MAIA Trusting [0–5]	3.28	1.19	2.92	1.37	-0.36	-0.87	0.16	0.26*	-0.26	

PROMIS scores are normalized to T-scores on a 0–100 scale. BL: baseline; FU: follow-up; ES: effect size.

*using Wilcoxon Signed Rank tests rather than Paired *t* tests for non-normal distribution according to Shapiro–Wilk W test.

Additional results in S1 Table

The following baseline variables correlated with %-improvements in PEG scores: income (*ρ* = -.49; *p* = .010, CPAQ Pain-Willingness at baseline (*r* = -.40; *p* = .031), whereas these other variables correlated with not being categorized as a responder (i.e., PEG score improvement of <30%): baseline neuropathic pain (*r* = .53; *p* = .004), widespread pain (r = .51; *p* = .005), PROMIS Anxiety (*r* = .49; *p* = .007), and PTSD as adult (*r* = .48; *p* = .045). We found no differences in absolute and percent PEG change scores between birth sex using regression analysis (*F(1*,*27)* = 0.40, *p* = 0.53 and *F(1*,*27)* = 0.09, *p* = 0.76, respectively).

As mentioned above, one study inclusion criterion was a low score for total MAIA and for Non-Distraction. Against our hypothesis, the scores for MAIA total and MAIA Not-Distracting did not have statistically significant changes, though about half of the participants had higher scores in both at follow up. The only MAIA scale that showed significantly improved scores was the Not-Worrying scale from 2.81 (±1.06) to 3.19 (±1.10; 95% CI 0.10, 0.67; *p* = .013; ES = .52.) MAIA Not-Worrying scores were not correlated with pain anxiety CPAQ total scores but negatively with the subscale of Pain Willingness (baseline *r* = .48; *p* = .008. follow-up *r* = .54; *p* = .002) and the Catastrophizing PCS total scores (baseline *r* = .43; *p* = .02. follow-up *r* = .80; *p* < .0001), most strongly (at follow-up *p* < .001) for Rumination and Helplessness. There were no significant pre-post changes for mean CPAQ Pain Willingness. Mean Rumination improved (from 3.00 ±2.07 to 2.17 ±2.04; 95% CI -1.5, -0.2; *p* = .018; *ES* -0.47). Only at follow-up MAIA Not-Worrying scores were negatively correlated with PROMIS Anxiety T-scores (*r* = -.49; *p* = .008), which at average showed no meaningful change.

Although not a major issue at baseline, widespread pain tended to be slightly less frequent at follow-up, whereas pain below the knee was reported as more frequent at the end of the study. Most of the other parameters did not show statistically significant or clinically meaningful changes. This included measures of physical function, pain self-efficacy, depression, positive and negative affect, sleep, fatigue, pain avoidance (PASS subscale), and mindfulness. An exception was the PROMIS measure for Ability to Participate in Social Roles and Activity, which improved by mean 3.9 points (95% CI 1.3, 6.6; *p* = .0050; ES = .58; T-scores). This change was correlated with change in pain intensity (*r* = -.45; *p* = .016), change in PEG scores (*r* = -.44; *p* = .018) and change in PROMIS pain interference (*r* = -.41; *p* = .030). Although mean physical function (by PROMIS) and mean pain self-efficacy did not change significantly, Social Role was correlated with physical function (baseline *r* = .69; *p* = .0001; follow-up *r* = .48; *p* = .010) and with pain self-efficacy (baseline *r* = .73; *p* < .0001; follow-up *r* = .57; *p* = .002).

Interestingly, scores for pain self-efficacy *at follow-up* were correlated with several MAIA scales: Not-Worrying (*r* = .48; *p* = .008), Attention Regulation (*r* = .51; *p* = .004) and Trusting (*r* = .45; *p* = .014), whereas the only MAIA scale score correlated with self-efficacy *before the intervention* was Noticing, and this latter correlation was negative (*r* = -.42; *p* = .029).

In further exploratory analysis, we assessed baseline variables for predicting treatment responders defined as ≥30% improvement in PEG scores. The odds of responding to the intervention were increased with higher $ income (OR = 1.02; *p* = .010), increase in MAIA Trusting (OR = 2.03, *p* = .059), increase in PROMIS Social Role (OR 1.35, *p* = .043) and decreased with baseline widespread pain (OR = .42, *p* = .21), baseline MAIA Body Listening (OR = .05, *p* = .027), higher expectations for percentage of pain relief (OR = .60, *p* = .019), PROMIS Anxiety (OR = .86, *p* = .033), PASS Escape-Avoidance OR = .81, *p* = .047), PASS Physical Anxiety (OR = .81, *p* = .030), and PTSD symptoms (OR = .56, *p* = .025) at baseline. Other variables did not predict a 30% PEG improvement response. Baseline MAIA Body Listening correlated with baseline PROMIS Anxiety (*r* = .47; *p* = .011).

When comparing pain intensity and interference data by questionnaire versus by EMA, both measures were correlated, although EMA data at baseline were lower than questionnaire data and at average even below our inclusion criteria (Table 3). Pre to post changes in mean EMA values improved but did not reach statistical significance.

**Table 3:** Correlation of 1 week of EMA data for pain intensity and pain interference with related questionnaire items “during the past 7 days”.

	1^st^ week of MyP			last week of MyP		
	mean		*P*	mean		*P*
Pain Intensity [0–10]	2.84 ±.23	*r* = .63	.0003	2.56 ±.26	*r* = .76	< .0001
Pain Interference [0–10]	1.91 ±.24	*r* = .62	.0004	1.58 ±.24	*r* = .47	.010

*r*: Pearson correlation coefficient

### Qualitative outcomes

Exit interviews lasted up to a maximum of one hour, depending on the participant’s willingness to share personal experience. We generated the following themes from our interviews with all participants here identified by their study ID in brackets after the quotation.

#### 1) Participants were aware of their usual habit of avoidance and found it challenging to focus on pain

*Definition*. The task of the phone app was to take a few minutes during the day to focus with curiosity one’s attention on the felt sensation of pain. Some participants appeared not to be able to do that—or to choose not to do that—due to highly demanding life circumstances (e.g., caretaking for parents with COVID; partner lost a job; childcare *and* job burden). All participants, however, felt that this approach was in stark contrast to their usual habit of getting on with life by generally ignoring the pain and avoiding paying any attention to it.

“I always thought if you ignore it, it’s just going to go away.” [135]

“Interesting: you’d think that trying to ignore it might be the way to go, but it’s definitely a different thing.” [87]

”Conscious focus on bodily sensations: that’s something that I have always run from. But it’s not really getting me anywhere. At this point I feel that’s gotten me to where I am now, and now that protective mechanism of not paying attention to bodily sensations is no longer serving me.” [10]

“In the back of my head I was always fighting it.” [78]

“I’ve always been a person that—I’ve just pushed through. Regardless of what the pain was, I pushed through. I had things to do, and I’m miserable, and you just put on your happy face and you just get through it as you’re sitting there wanting to cry inside.… and with this [app], what it’s shown me: pushing through is not always the answer. You’ve got to take that time to recognize.” [141]

“The big takeaway is not to ignore your pain” [135]

“Sometimes you would just continue to push through because you are at a much more important task… . Those things are hard to change.” [19]

“I cannot do the app, totally engaged in something else, I am doing other things… that whole life.” [95]

“I definitely felt that resistance… . Old habits die hard.” [10]

*Discussion*. Old habits are not easy to change. To some, the patterns may seem impossible to change, particularly when overwhelmed by stress and life’s demands. But even old dogs can learn new tricks. Participants may recognize that their customary habits of distraction and trying to ignore their pain do not help their chronic condition (thus confirming current research evidence [8, 48]), and are willing to try something new.

#### 2)Participants were surprised about the variance in pain sensations over time

*Definition*. When participants paid attention to the different elements, components, qualities, or characteristics of their pain sensations, they reported being surprised by the discovery that pain sensations change in ways they had not expected.

“A big one for me was noticing how the pain could shift just within 30 seconds that I’m really consciously focusing on it. My experience of the pain would fluctuate.” [10]

“For me the big discovery was that my pain is different every day. Literally different every single time I concentrate and am mindful about what it feels like, where it is, is it moving, what’s the density, etc.?” [128]

“There was a lot of variation. For me it was a new discovery because the pain is different. Whereas before, I am in pain, I am uncomfortable, without really taking the time to identify what type of pain it was. I think that was an eye opener for me because I never really thought about it from the standpoint of is this something that’s radiating outwards. Is it something that is pulsing? Is this a tension? And it did fluctuate, and then taking the time for me going to that next step of what was your day like today that made this pain different than the pain from yesterday?” [141]

*Discussion*. Pain is not a “thing.” What we call pain is a fluctuating experience that we tend to reify and objectify. This has been previously acknowledged primarily by philosophers [49, 50], one of whom actually was involved in the most recent international discussion of the current understanding and definition of pain [51]. Relating noticed variations in pain quality and/or intensity to what happened in preceding hours physically or emotionally may be a learning opportunity.

#### 3) Participants were surprised that the intensity of pain could change by focusing on it

*Definition*. For some participants, a major surprise was that just focusing on the sensation itself and inquiring into its elements could change the pain intensity. This reportedly occurred for a few participants within a minute and/or over weeks of using the app.

“Always, I feel my back is numb. But lately when I sit, I feel like, I don’t know how to describe it, but you know when you sit a lot and your legs get numb, and then you have to stand up because you have to wake up the leg? So, I feel like that, like my back is waking up… it’s good because I feel some relief. I feel like something is going away.’[26]

“I think with the app taking that time mentally to connect [with the body] has decreased my pain and my emotion and my muscle tightness.”[29)

“I think that just by focusing on it and trying to really understand it and feel it helps to lessen it.” [140]

“I did start observing that after I sat down and thought about it, the pain was lessened, if not gone completely. And that was kind of the aha moment, there is actually something to this.” [87]

“When I was really, really trying to focus in on the pain, and identify it and all this, and I had this period of time, and this hadn’t happened in a year and a half—I’ve had this for going on two years now—where all of the sudden the pain diminished substantially… And that was a great pleasure.” [86]

*Discussion*. The mindful interoceptive exposure task of the app appears to loosen a previously more rigid reification/objectification of the pain experience, which is consistent with traditional philosophies of change (Herakleitos, *panta chorei*) [52] and impermanence (Buddhist, *anicca*/ *anitya*) [53]. Although this experience was new to our participants, it has been described previously [27, 54, 55]. We will discuss this further in the general discussion below.

#### 4) Pain has a 3D shape

*Definition*. The concept that the sensation of pain occupies a three-dimensional space within our three-dimensional body made sense to all participants, although it was new to most participants’ felt experience. Participants, who were able to hold attention with a sensory perceptive focus, were surprised by the qualities and characteristics of the pain’s phenomenology, morphing into various shapes with a surprising variety of densities, transparency, temperature, and colors. However, to be able to engage in this microphenomenological sensory focus of deep inquiry into the uncomfortable pain sensation, some participants required verbal one-on-one guidance during the exit interview.

“Surprise that pain had all kinds of different shapes. It wasn’t ever really the same. Before the app, I was not aware of it, it was just like, ‘it hurts.’” [126]

Examples for the shapes of pain: A ball [6, 26]; an amoeba, almost like a living, breathing thing, changing shape or not [56]; radiating rings like a tree trunk, white radiating off into grey and very dark at the outer edges [86]; a brick [126]; a pool table ball [126]; a pole, sometimes very thin like a needle or a pencil, kind of dull and poking [126]; a cylindrical shape with a blurry/fuzzy outside with little pain branching out at segments of the spine [128], a star, white, radiating, cold, a space rather than a light [129]; a flash, a burst [129]; an energy center [86]; an oval shape, horizontal, bluish, opaque see through, feeling alive with energy in it, “it’s my energy, a part of me, beautiful” [135]; an oval like an American football [138]; a stun gun [138]; a hand strangling my vertebra, a cramp [138]; a hot nail in my back [138]; a tight superball with a hard inner core, like a heavy-duty golf ball-type material inside, coal black, and away from the center more like grey rubber and on the outside whit and fluffy, feeling dead, an alien pod [141]; watercolors on a piece of white paper, pink in center, bleeding red on the edges [184].

And when the pain diminishes while focusing on the pain:

“I focus in the spine… Because I never feel that, ‘oh my god, what’s going to happen now?’…The butterflies, they were stuck in my back, and they start flying… purple butterflies flying away… When I feel that sensation, I feel I can breathe better… I feel, it’s hard to say, like when you open a Coca Cola. Pshhw… like the air comes inside my back… this is really amazing.…it helps a lot.” [26]

*Discussion*. Participants explored the three-dimensional shape of their pain experience. This was further explored during the exit interview, where we found that a microphenomenological inquiry into the lived experience of pain could reveal previously unnoticed details. This is consistent with findings from other research using microphenomenology interviewing technique [57] and some traditional or contemporary contemplative practices (e.g., https://www.diamondapproach.org/method). To our knowledge, the variety of elements of the pain experience was rarely described before [27], but never as patient-reported real-life experience.

#### 5) Participants had difficulties finding words to describe sensory awareness: Either just using the word “thinking” for any mind-body activity or by attempting to specify “*really*, *really* thinking”; “*really* focusing”

*Definition*. Since the task included a differentiation (a “breaking down”, “deconstructing”) of the pain sensation into its various elements, components, qualities, or characteristics, the mental activity associated with this task was generally reported by participants as “thinking” about pain. They reported that they perhaps have had thoughts about pain but never before were “*really* thinking” about it, “*really* focusing” on it. They made a distinction between “thinking” of pain, as in “Oh, this is my pain! Nothing new about it” and even identifying with it (“I am the girl with pain”), on one hand, and “*really*, *really* thinking” about pain, such as “trying to be with it, analyze it, understand it,” on the other. They lacked appropriate language to differentiate between sensing with immediacy and curiosity and thinking about pain with avoidance of “*actually* feeling” it. They often gave up after grappling for words, recognizing the limitation of their vocabulary, which could potentially discriminate distinct cognitive elements or mental processes involved in mindful interoceptive exposure.

“*Actually* **thinking** about it, not just **thinking** about: ok, I have pain.” [100]

“Thinking about it…, so sometimes I think I’m mixing up the sensing and the **thinking** about it, but the **thinking** about it, then is that truly what I am feeling?… Periods where you’re trying to **think** about it, whether you’re really sensing versus what you **think** about it, but… those two things are so intertwined that trying to keep them unknotted… That whole approach to **thinking** about it!” [78]

“For some reason identifying it, **thinking** about it, sometimes kind of meditating on it, it helped it… . I found it interesting. It’s almost like while I was focusing in on it, it may have become a little bit less because I’m **thinking** about it. I don’t know quite how to explain it, but… . I was with it, yes.” and later the same participant states: “I felt very present. I didn’t feel distracted, I didn’t feel like I was **thinking**.” [86]

“You’re going to be **thinking** about your pain, I want you to focus and **think** about it with curiosity. That curiosity is kind of a unique word and so it makes you **think**, be curious about this. And I **think** that word does help you kind of get in the right mindset to focus on what you’re supposed to focus on. Without that, it would just be **think** about it, but **thinking** with curiosity definitely has a triggering effect that makes me focus a little more.” [87]

“*Really* **thinking** about it.” [87]

“I have never really thought about **thinking** about it; it was interesting.” [110]

“That’s when I start *really* **thinking** about it. Beforehand I kind of quickly **think** about it, or **not think** about it.” [110]

“I think just being more present with it and *really* **thinking** about how it’s feeling internally or inside.” [126]

*Discussion*. It seems that the way the word “thinking” is used by our participants includes all mental processes that are in contrast with sensory awareness of pain and in line with distraction from that unpleasant sensory experience. As described in research on mindfulness and meditation, novices to these approaches commonly are unable to differentiate thinking about oneself (including pain) from the direct sensory experience of oneself (including pain) [17]. Even experts had to apply considerable effort to agree on consistent language that distinguishes functional dimensions (object orientation, de-reification, and meta-awareness) coupled with various qualitative dimensions (aperture, clarity, stability, and effort), which in turn are associated with specific neurological brain activities [58]. The philosopher Heidegger (1951–52), in his classical lecture series “Was heisst Denken? [What is called Thinking?]” describes a similar lack of clarity in the common use of the word and then discerns two kinds of thinking, “calculative thinking” and “meditative thinking” [59, 60]. As referenced in the introduction, the psychologist Marc Williams made a potentially related intriguing observation in his review of studies on mindfulness interventions for pain, namely that thinking about pain may be a subtle form of distraction from actually sensing it [16]. This extends beyond pain to “thinking” about emotions [16] and our self-concept [61]. There were few exceptions:

”I think not necessarily obviously thinking about it, but paying attention to how it feels like.” [126]

#### 6) The participants described a sequential process of meeting initial resistance, intentionally stopping, slowing down, sitting still, and focusing

*Definition*. Participants described the activities that were involved with the app and its task as organized in a diachronic sequential order: 1) “stop, take time, step away”; 2) “sit, slow down”; 3) “tune in, be with it, *really* focus on pain sensation, *really* think about it”. Some participants added two additional steps: 4) “shut everything else out, shut down your brain”; 5) “let your body speak, and listen.” For a focus on unpleasant bodily sensation, such as pain, participants encountered an initial resistance, that had to be overcome to move from experiential avoidance to mindful awareness.

“Stop, taking time [6, 29, 87, 126, 141], step away [126], sit, slow down [141], calm down [25], going inward [29], focus on pain [6, 10, 16, 19, 86, 89, 100, 126, 128, 141, 148, 161, 167], really, really trying to focus in on the pain [86, 126, 161], tune in [167], honing in [14], be with it [102, 161], think about it [6, 10, 29, 78, 86, 87, 89, 102, 110, 126, 128, 148], being mindful about what it feels like [128].”

A few participants went further and noted:

“30 seconds was not enough time to just really shut down your brain.” [110]

“Really, the important thing is spending that 30 seconds letting your body speak, and just shutting everything else out.” [128]

But this was rather difficult for some:

“Focus on pain frustrates me; my mind never stops.” [92]

*Discussion*. Several sequential steps appear to be necessary to gain benefits from mindful interoceptive exposure to pain. Overcoming experiential avoidance of negative emotions, which, according to Craig [62], include feeling pain, is a common theme in established behavioral approaches to pain (cf. ACT, exposure-based CBT, and other psychotherapies since Freud).

#### 7) Recognition of the role of breath in coping with pain

*Definition*. Focusing on and becoming aware of breathing appears to have facilitated the letting go of tensions that participants noticed in their body as associated with pain.

“Stop and breathe!” [95]

“Connecting the breathing with focusing on pain.” [140, 167]

“The app helped to taking time to slow down, sit and just breathe.” [19, 29, 41, 95, 138, 141, 167]

“Breathe and let emotions come in!” [29]

“I need to stop and breathe.” [148]

“I focus what the breathing is doing to it, it drops the tightness, relaxes a bit, coming from the outside. As soon as it hits the inside there is a release of tension.” [141]

“Acknowledging it and then letting go, instead of holding breath with frustration.” [25]

*Discussion*. That awareness of breath and conscious or controlled breathing can be helpful for chronic pain has been found in prior research [63, 64].

#### 8) The attentional focus on pain (i.e., mindful interoceptive exposure) may moderate affect and the appraisal of pain

*Definition*. Participants reported that using the phone app had effects on their affect and pain-associated emotions. They reported a reduction in the threat value of their pain, being calmer, less scared of pain, less cranky, and less frustrated.

“Focus on my body instead of all the thought brings me into the center of myself and brings awareness of how emotions are creating tightness with pain in my body.” [29]

“I was afraid to relax, now less afraid, more trusting in myself.” [78]

“If there is a relief from pain, hope comes up [10, 19, 86], and trust in oneself [78]

“Losing fear about pain sensation, I had deprived myself of moving.” [126]

“It’s painful, so what? I am not a victim of my pain, I can tolerate it and go for a walk. I feel better about myself. This is not something I should let command my life” [138]

“A little more trusting towards my body, more confidence.” [148]

“Less afraid of what might be in the future.” [161]

“A little less cranky about pain.” [164]

“Looking at my pain, analyze it, calms me down instead of freaking me out.” [167]

“The whole experiment forced me to look at it and sit with it, and in the end it makes you feel less afraid, more comfortable with your pain, if that makes any sense.[167]

“Things are not as dangerous as my mind was telling me.” [78]

Although most participants found some relief with the phone app, a few felt that bringing more awareness to their experience of pain was making it more difficult to ignore their pain during their daily work activities and rejected or resisted practicing mindful attention.

*Discussion*. this finding is consistent with research on mindfulness and its effect on emotions and pain, [65–67] is in line with reduced worry and rumination found in the quantitative data, and with recent research that showed different emotion-related phenotypes for traits of interoceptive skills [68].

#### 9) Participants may use visualization when inquiring into their sensory pain experience

*Definition*. The app asked to focus into the very center of the pain sensations as taking up a circumscriptive space inside the body. To describe the shape, participants used visualization and created 3D models in their mind intended to match interoceptive sensation with a visual image. However, when asked whether they *see* these shapes, participants provided inconsistent statements: seeing the shape of pain inside their body, or in their mind, or described them as a “feeling” in space, and at times the same participant first confirmed the use of visualization and then denied it. Imagination and spacious awareness of pain were difficult to articulate.

“Visualizing in my head, willing the body to relax.” [29]

“Visualizing but not always able to get that. If visualizing worked, did not feel distracted, it was the moment, did not feel like thinking, remembering from something else.” [86]

“Creating a shape [for the pain] in my head. Visualization is my connection to the body.” [128]

“I visualize what I’m picturing in my back, a 2D image shows up, I locate the spot then I start scanning, trying to build this 3D image in my head.” [128]

“Imagining the space, the pain takes in my body.” [129]

“I visualize the area that is in pain. It’s as if pain were a substance, that’s how I visualize it.” [138]

“I visualize it in front of me, the back of my eyelids, really pretty.” and then she explains: “I don’t visualize it, I don’t see it in my back, it’s just in my head, it develops on its own.” [148]

*Discussion*. Describing the process that is labeled as visualization of an interoceptive sensation that is felt in the back appears difficult. Where is the image located? What does it mean if it is located in the head? Is directing the focus of sensory perception towards the shape of pain asking for a visual image? How does a physical sensation felt inside the body space relate to or get transformed into an image? Some of the descriptions were reminiscent of Annie Dillard’s essay “on seeing” [69], where she describes two kinds of “seeing” in nature. One is imbued with and obscured by our incessant thinking, impressed and colored by priors and predictions; the other is experienced with perceptual immediacy in the present moment. This is consistent with the predictive coding model of interoception [70] and pain perception, specifically [71].

#### 10) Listen to your body: The body is communicating and “demanding” to be recognized and acknowledged

*Definition*. A few participants described a kind of communication with their own body, in which the body apparently wants to be recognized, given one’s full attention, and may stop “ramping up the pain” when its pain is acknowledged. The body is viewed as an agent with a voice that uses pain as its language demanding to be listened to, and only then can relax when the body’s owner is fully present with it.

“The body is communicating something.” [10]

“It is about listening better to my body, to what it’s trying to tell me, trying to interpret what it means.” [89]

“Taking time, slow down, breathe, relax a little, unwind helped my body to say, ok, you are recognizing you’re in pain, ok, we are happy with that, we don’t have to keep ramping up the pain level to make you pay attention.” [141]

“The thinking, it’s stopping and just listening, stop listening, don’t think! Let your body communicate up, let your body communicate here… stop scanning, just listen!” [128]

“Acknowledge the pain is a whole new perspective.” [25, 110]

“The body gives an answer. I think the hard part is just finding the words for it.” [128]

“The big takeaway is not to ignore your pain” [135]

*Discussion*. That the body “wants to be listened to” has been proposed previously as a stage in the developmental model of bodily awareness, framed as the dialectic of body and self [72, 73]. It is viewed as being an essential element in the concept of self-reported interoceptive awareness [68, 74].

## General discussion

We explored whether a mindfulness-based interoceptive exposure treatment would be feasible, acceptable and benefit patients with cLBP. Our proof-of-concept study of the MyP smart phone application demonstrated that such innovative approach to cLBP is acceptable to patients, despite it being in marked contrast to the habitual coping style of ignoring pain and distraction. The majority of participants reported clinically meaningful improvements in their pain.

Although drop-out rates in trials of apps can reach 50% [75], only two participants of the 31 (6.5%) who started using the app dropped out. Regarding recruitment and adherence, we found the app-based attention task to be both feasible and acceptable. The instruction asked for using it twice daily upon built-in notifications and to use the app additional times in between. The mean number of app entries were consistent with the twice daily notifications, but the variance for the number of entries was rather large. This may in part have been due to the fluctuating course of pain and the relatively low level of pain intensity at baseline: for entry criteria during recruitment, we required a minimum NRS pain level of only 3 out of 10. Mean pain intensity was reported as 4.8 on NRS. However, EMA data showed that average pain intensity during the first week of using the app was rated only 2.84 (±0.23) and pain impact 1.91 (±0.24). Future studies may include participants with higher average pain of >3 at baseline.

We received valuable feedback on how to improve this prototype of a phone app in its technical and aesthetic aspects. The app was built on Qualtrics, lacked interaction among app users, and presented itself with rather crude design compared to what is available today in our rapidly evolving space of digital mHealth apps [76, 77]. It could be significantly enhanced by adding more variety of content and color, by adding links to relevant pain education and by adding more opportunities for two-way interactive communication with the research team. This could potentially increase the frequency of app usage during the day. A few participants noted that they “did not get into it” and used the app much less frequently than proposed. Although the app was only minimally supported by weekly check-in calls with human interaction, its basic version appeared helpful to participants and may serve as prototype for further development.

Overall, for most participants pain intensity and pain impact improved according to NRS pain and PEG scores. This improvement was statistically significant and clinically meaningful [78]. Pain Impact, the outcome measure recommended by the NIH Task Force, also improved, whereas PROMIS Pain Interference had average improvement that was not considered a clinically meaningful difference and was not statistically significant. However, because this was a single-arm study, we cannot rule out improvements due to natural history, non-specific placebo effects, regression to the mean, and Hawthorne effects. Although EMA measures for mean pain intensity and impact entered during the first and last week of using the app did not show statistically significant improvements, averaged over 7 days they were correlated with questionnaire items for the past week with the highest correlation for pain intensity seen at the end of the intervention. This may be partially explained by EMA measures being collected and averaged in the first week *after* starting the app, whereas baseline questionnaire scores were collected “at average for the past week” preceding enrollment. EMA pain scores for the first week of using the app frequently were entered weeks after enrollment and were lower than during enrollment.

The qualitative interviews partly explained why three participants reported slightly worsened pain at follow-up: They had noted their resistance against the attention task. For one “it became a chore” and “it was just hard to like take the time to do it” (ID 41); they “rushed through it" because “you are at a much more important task that you’re doing” when she “was in a middle of something”, “multitasking” and “wasn’t really totally focused on just answering those app questions or doing the exercise” (ID 19); “time to look at my pain… with a full-time job or something like that: I don’t have that [time] right now”(ID 167). However, during her interview the latter participants reported reduced pain anxiety contradicting her questionnaire responses of unchanged pain anxiety scores a week earlier.

Although across our participants mean questionnaire scores for pain-related physical anxiety or for more general anxiety (both of which were low on average at baseline) did not show significant improvement, rumination and worries about pain diminished. This was further illustrated in the exit interviews (see Theme #8 above). This is in line with prior research on catastrophizing [79] and worry [80] and a study that showed that cognitive defusion, a form of psychological distancing from internal experiences, was more important for sensory-affective uncoupling of pain and pain reduction during mindfulness meditation than changes in mindfulness questionnaire scores [81]. Whether attention to body sensations is positively or negatively associated with anxiety depends on the style of attention [74, 82, 83]. We would not expect that relatively brief mindful attention to pain sensations would reduce general anxiety, but mindful attention to anxiety-related symptoms with decentering and sensory-affective uncoupling in more intensive mindfulness interventions has shown benefits with anxiety [84].

Pain Self-Efficacy, like numerous other psychological variables that had consistently shown associations with changes in pain outcomes in prior research with cLBP, did not change significantly. However, *at follow-up* Self-Efficacy scores were correlated with less worrying, higher Attention Regulation and Trusting, whereas *before the intervention* only reduced Noticing was negatively correlated with self-efficacy. This change in correlations may indirectly imply a learning process for pain self-efficacy along dimensions of interoceptive awareness.

Exploring baseline variables for their association with the outcome of PEG scores, higher scores on MAIA Body Listening and lower scores on experiential avoidance and PROMIS or PASS Anxiety scores reduced the odds of a favorable outcome, as well as having widespread pain and/or a history of PTSD, appear to indicate that if anxiety-driven hypervigilance and experiential avoidance are not specifically addressed, patients may less likely benefit from this approach.

However, we could not confirm our hypothesis that the app would increase mindful interoceptive awareness as measured by the MAIA-2. Our study was advertised as “a study using a new smartphone-based attention task that may help you better cope with your pain!” Mindfulness was not mentioned in it nor in the consent form. It was only briefly mentioned in the introduction session and in the subsequent 6-page summary handout as “paying close and mindful attention to the details of the sensation that we call pain (sometimes called Somatic Tracking),” may restore diminished brain regions, retrain, and reprogram the signal processor, and dial down the signal. We define “close and mindful attention” as “noticing how the pain sensation actually feels.” Participants with regular mindfulness and/or meditation practice and with above average levels of interoceptive awareness, a trait or skill that is trained with mindfulness practices [18, 85], were excluded from study participation. Meta-awareness, Decentering, or De-Reification, central aspects of mindfulness practices, were not explicitly taught in the 1-hour introduction. Equanimity in pain observation and engaging with curiosity and non-reactivity was recommended but never entrained. Using the phone app-based attention practice did not increase average levels of mindfulness or interoceptive awareness in our participants assessed by standard questionnaires (5 scales of FFMQ and 8 scales of MAIA) with one exception: after the attention practice participants apparently worried less about unpleasant pain sensations as indicated by MAIA Non-Worrying. In the future, more frequent human support with more interactions with study staff may improve the participants understanding of the interoceptive exposure task and more specifically train mindful attention. It would be interesting to integrate our phone app intervention with a regular 8-week mindfulness training and to assess the efficacy of such a combination.

Although the intervention did not equal a brief mindfulness intervention, it was based on the theoretical concepts underlying a mindful interoceptive attention training for chronic pain. The results are not completely unexpected, as previous studies have found that *initial brief* mindfulness instructions in untrained individuals can have negative effects on pain perception [86, 87]. For a training that requires additional self-regulatory attention resources, individuals with limited self-regulatory capacities at baseline may quickly reach personal limits [86]. We only recruited participants who scored below average in interoceptive awareness, which included Self-Regulation and Non-Distraction. Furthermore, other studies have noticed that mindfulness-naïve participants may overestimate their self-reported mindfulness skills before initially applying and learning such skills [88] and feel they need to correct the self-assessment downwards *after* training of mindfulness skills [89, 90], which may reduce the ability to see improvements in MAIA and FFMQ scores. In contrast to the quantitative data not showing significant improvements in the below-average baseline MAIA scores, the qualitative interviews showed that most participants were able to—at least temporarily—shift the prior habit of ignoring and distracting from pain to giving it full attention.

In line with the lack of apparent changes in mindfulness, the qualitative interview questions did not explicitly ask about mindfulness or interoception skills that participants might have acquired. Also, the interviewer had not seen any questionnaire data before the interviews that might have led to more specific interview questions. However, the interviews showed that the degree to which participants understood the rationale behind the study varied widely, and participants generally had difficulties expressing themselves on topics of attention self-regulation, skills of perception and cognitive changes (see Themes #5 and 9 above).

The qualitative interviews included an innovative interview technique based on micro-phenomenology [45]. During this guided interview component, participants were able to inquire into the experience of pain and report in more detail what they discovered about the dynamic of pain with mindful interoceptive attention. They described a coherent sequential process of stopping, slowing down, sitting still, and focusing, and the challenge of overcoming resistance to focus on pain. Using breath awareness and slowing down breathing appeared to support the focus [91]. When able to overcome the initial resistance, they were surprised by the great variance of their pain experience and its response to their own attention. A particularly surprising discovery was that pain has a describable shape. Participants were able to report a great variety of 3D pain shape experiences. Paying attention to pain in this curious and mindful way took away some of the threat value of pain thereby changing the appraisal, which is consistent with current theories of how mindfulness alters pain perception [66]. A few participants even discovered an aspect of beauty in the shape of their pain. As a result, patients who improved in pain outcomes appear to have learned to trust their bodies more and increased their ability to take part in social life (by MAIA Trusting and PROMIS Social Role).

### Limitations

This was a single-arm study, which has important limitations in assessing efficacy. In particular, the pre-post changes we assessed provide very preliminary data on the effects of the intervention we studied, and the absence of a control group mean that we cannot distinguish changes in outcomes due to natural history or non-specific effects of participation in a research study from the intervention itself. We also did not adjust for multiple comparisons, as this was a pilot study and not an efficacy study. Our finding of no differences in outcomes by participants’ sex at birth may be limited by the small sample size. A major limitation was the non-industrial basic design of the app and the small amount of human support per phone and online video meetings. However, the study provided ample suggestions for improvements and first-person insights into the cognitive processes involved in changing the common coping style of distraction from pain to a mindful exploration with interoceptive exposure.

## Conclusions

In this single-arm proof-of-concept study we confirmed the feasibility and acceptability of an innovative app-based approach to cLBP that is based on mindful interoceptive exposure to pain, which is in marked contrast to the habitual coping style of ignoring pain and distracting oneself from it. Patients made new discoveries about previously unnoticed details of their pain experience, e.g. about the shape of pain. Our study shows that paying attention to the details of the pain experience with curiosity may be an alternative approach to the management of chronic low back pain for patients, who are not overwhelmed by demands of daily life and have not widespread pain and/or a high degree of anxiety-driven hypervigilance. By decreasing worry and rumination, this approach may work by sensory-affective uncoupling not unsimilar to mindfulness approaches [81] and could potentially integrated into conventional mindfulness-based practice trainings.

## Supporting information

S1 File Study protocol. (DOCX)

S2 File Handout: Neuromatrix of pain. (DOCX)

S3 File Measures: List of questionnaires. (DOCX)

S1 Table Pre-post changes in pain measures and psychological variables (unabbreviated version of Table 2 in manuscript). (DOCX)

## References

[1] Roseen EJ, Smith CN, Essien UR, Cozier YC, Joyce C, Morone NE, et al. Racial and Ethnic Disparities in the Incidence of High-Impact Chronic Pain Among Primary Care Patients with Acute Low Back Pain: A Cohort Study. Pain Med. 2023;24(6):633–43. doi: 10.1093/pm/pnac193 36534910 PMC10233486

[2] Dieleman JL, Cao J, Chapin A, Chen C, Li Z, Liu A, et al. US Health Care Spending by Payer and Health Condition, 1996–2016. JAMA. 2020;323(9):863–84. doi: 10.1001/jama.2020.0734 32125402 PMC7054840

[3] Raad M, Pakpoor J, Harris AB, Puvanesarajah V, Marrache M, Canner JK, Jain A. Opioid prescriptions for new low back pain: trends and variability by state. The Journal of the American Board of Family Medicine. 2020;33(1):138–42. doi: 10.3122/jabfm.2020.01.190254 31907255

[4] Chau A, Steib S, Whitaker E, Kohns D, Quinter A, Craig A, et al. Theoretical Schemas to Guide Back Pain Consortium (BACPAC) Chronic Low Back Pain Clinical Research. Pain Med. 2023;24(Suppl 1):S13–S35. doi: 10.1093/pm/pnac196 36562563 PMC10403312

[5] Mescouto K, Olson RE, Hodges PW, Setchell J. A critical review of the biopsychosocial model of low back pain care: time for a new approach? Disabil Rehabil. 2022;44(13):3270–84. doi: 10.1080/09638288.2020.1851783 33284644

[6] Kohl A, Rief W, Glombiewski JA. Acceptance, cognitive restructuring, and distraction as coping strategies for acute pain. The journal of pain: official journal of the American Pain Society. 2013;14(3):305–15. doi: 10.1016/j.jpain.2012.12.005 23352770

[7] Goubert L, Crombez G, Eccleston C, Devulder J. Distraction from chronic pain during a pain-inducing activity is associated with greater post-activity pain. Pain. 2004;110(1–2):220–7. doi: 10.1016/j.pain.2004.03.034 15275771

[8] Mehling WE, Ebell MH, Avins AL, Hecht FM. Clinical decision rule for primary care patient with acute low back pain at risk of developing chronic pain. The spine journal: official journal of the North American Spine Society. 2015;15(7):1577–86. doi: 10.1016/j.spinee.2015.03.003 25771757 PMC4475413

[9] Hasenbring MI, Verbunt JA. Fear-avoidance and endurance-related responses to pain: new models of behavior and their consequences for clinical practice. The Clinical journal of pain. 2010;26(9):747–53. doi: 10.1097/AJP.0b013e3181e104f2 20664333

[10] Mehling WE, Daubenmier J, Price CJ, Acree M, Bartmess E, Stewart AL. Self-reported interoceptive awareness in primary care patients with past or current low back pain. Journal of pain research. 2013;6:403–18. doi: 10.2147/JPR.S42418 23766657 PMC3677847

[11] Johnson MH. How does distraction work in the management of pain? Current pain and headache reports. 2005;9(2):90–5. doi: 10.1007/s11916-005-0044-1 15745617

[12] Rad AAW P.-M. Insights into pain distraction and the impact of pain catastrophizing on pain perception during different types of distraction tasks. Frontiers in Pain Research. 2024;5(2024). 10.3389/fpain.2024.1266974 PMC10844527 38322588

[13] Zorn J, Abdoun O, Bouet R, Lutz A. Mindfulness meditation is related to sensory-affective uncoupling of pain in trained novice and expert practitioners. European journal of pain. 2020;24(7):1301–13. doi: 10.1002/ejp.1576 32311185

[14] Kim D, Woo CW, Kim SG. Neural mechanisms of pain relief through paying attention to painful stimuli. Pain. 2022;163(6):1130–8. doi: 10.1097/j.pain.0000000000002464 34433768

[15] Cioffi D. Beyond attentional strategies: cognitive-perceptual model of somatic interpretation. Psychological bulletin. 1991;109(1):25–41. doi: 10.1037/0033-2909.109.1.25 2006227

[16] Williams JM. Mindfulness and psychological process. Emotion. 2010;10(1):1–7. doi: 10.1037/a0018360 20141295

[17] Farb NA, Segal ZV, Mayberg H, Bean J, McKeon D, Fatima Z, et al. Attending to the present: mindfulness meditation reveals distinct neural modes of self-reference. Social cognitive and affective neuroscience. 2007;2(4):313–22. doi: 10.1093/scan/nsm030 18985137 PMC2566754

[18] van der Velden AM, Scholl J, Elmholdt EM, Fjorback LO, Harmer CJ, Lazar SW, et al. Mindfulness Training Changes Brain Dynamics During Depressive Rumination: A Randomized Controlled Trial. Biol Psychiatry. 2023;93(3):233–42. doi: 10.1016/j.biopsych.2022.06.038 36328822

[19] Maloney S, Surawy C, Martin M, Montero-Marin J, Kuyken W. The State- and Trait-Level Effects and Candidate Mechanisms of Four Mindfulness-Based Cognitive Therapy (MBCT) Practices: Two Exploratory Studies. Mindfulness. 2023;14(9):2155–71. doi: 10.1007/s12671-023-02193-6 37795338 PMC10545571

[20] Garland EL. Mindfulness-Oriented Recovery Enhancement: An Evidence-Based Social Work Intervention for Addiction, Stress, and Chronic Pain. Soc Work. 2023;68(2):171–4. doi: 10.1093/sw/swad008 36728495

[21] Van Ryckeghem DM, Van Damme S, Eccleston C, Crombez G. The efficacy of attentional distraction and sensory monitoring in chronic pain patients: A meta-analysis. Clinical psychology review. 2018;59:16–29. doi: 10.1016/j.cpr.2017.10.008 29126746

[22] Rivest-Gadbois E, Boudrias MH. What are the known effects of yoga on the brain in relation to motor performances, body awareness and pain? A narrative review. Complementary therapies in medicine. 2019;44:129–42. doi: 10.1016/j.ctim.2019.03.021 31126545

[23] Salmon PL E.; Jablonski M.; Sephton S. E.;. Yoga and mindfulness: clinical aspects of an ancient mind/body practice. Cogn and Behav Practice. 2009;16(1):59–72.

[24] Villemure C, Ceko M, Cotton VA, Bushnell MC. Insular Cortex Mediates Increased Pain Tolerance in Yoga Practitioners. Cerebral cortex. 2013. doi: 10.1093/cercor/bht124 23696275 PMC4153807

[25] Voss S, Boachie DA, Nieves N, Gothe NP. Mind-body practices, interoception and pain: a scoping review of behavioral and neural correlates. Ann Med. 2023;55(2):2275661. doi: 10.1080/07853890.2023.2275661 37939212 PMC10768869

[26] Miettinen OS, Caro JJ. Medical research on a complaint: orientation and priorities. Ann Med. 1989;21(5):399–401. doi: 10.3109/07853898909149228 2532530

[27] Cayoun B, Simmons A, Shires A. Immediate and lasting chronic pain reduction following a brief self-implemented mindfulness-based interoceptive exposure task: a pilot study. Mindfulness. 2017; doi: 10.1007/s12671-017-0823-x

[28] Deyo RA, Dworkin SF, Amtmann D, Andersson G, Borenstein D, Carragee E, et al. Report of the NIH task force on research standards for chronic low back pain. Spine. 2014;39(14):1128–43. doi: 10.1097/BRS.0000000000000434 24887571

[29] Mehling WE, Acree M, Stewart A, Silas J, Jones A. The Multidimensional Assessment of Interoceptive Awareness, Version 2 (MAIA-2). PloS one. 2018;13(12):e0208034. doi: 10.1371/journal.pone.0208034 30513087 PMC6279042

[30] Qualtrics [Internet]. 2020.

[31] Ostelo RW, Deyo RA, Stratford P, Waddell G, Croft P, Von Korff M, et al. Interpreting change scores for pain and functional status in low back pain: towards international consensus regarding minimal important change. Spine. 2008;33(1):90–4. doi: 10.1097/BRS.0b013e31815e3a10 18165753

[32] Althouse AD. Adjust for Multiple Comparisons? It’s Not That Simple. Ann Thorac Surg. 2016;101(5):1644–5. doi: 10.1016/j.athoracsur.2015.11.024 27106412

[33] Harris PA, Taylor R, Thielke R, Payne J, Gonzalez N, Conde JG. Research electronic data capture (REDCap)—a metadata-driven methodology and workflow process for providing translational research informatics support. J Biomed Inform. 2009;42(2):377–81. doi: 10.1016/j.jbi.2008.08.010 18929686 PMC2700030

[34] Batorsky A, Bowden AE, Darwin J, Fields AJ, Greco CM, Harris RE, et al. The Back Pain Consortium (BACPAC) Research Program Data Harmonization: Rationale for Data Elements and Standards. Pain Med. 2023;24(Suppl 1):S95–S104. doi: 10.1093/pm/pnad008 36721327 PMC11288398

[35] Krebs EE, Lorenz KA, Bair MJ, Damush TM, Wu J, Sutherland JM, et al. Development and initial validation of the PEG, a three-item scale assessing pain intensity and interference. Journal of general internal medicine. 2009;24(6):733–8. doi: 10.1007/s11606-009-0981-1 19418100 PMC2686775

[36] Chiarotto A, Vanti C, Cedraschi C, Ferrari S, de Lima ESRF, Ostelo RW, et al. Responsiveness and Minimal Important Change of the Pain Self-Efficacy Questionnaire and Short Forms in Patients With Chronic Low Back Pain. The journal of pain: official journal of the American Pain Society. 2016;17(6):707–18. doi: 10.1016/j.jpain.2016.02.012 26975193

[37] Fish RA, McGuire B, Hogan M, Morrison TG, Stewart I. Validation of the chronic pain acceptance questionnaire (CPAQ) in an Internet sample and development and preliminary validation of the CPAQ-8. Pain. 2010;149(3):435–43. doi: 10.1016/j.pain.2009.12.016 20188472

[38] Kamper SJ, Maher CG, Mackay G. Global rating of change scales: a review of strengths and weaknesses and considerations for design. J Man Manip Ther. 2009;17(3):163–70. doi: 10.1179/jmt.2009.17.3.163 20046623 PMC2762832

[39] McWilliams LA, Kowal J, Wilson KG. Development and evaluation of short forms of the Pain Catastrophizing Scale and the Pain Self-efficacy Questionnaire. European journal of pain. 2015;19(9):1342–9. doi: 10.1002/ejp.665 25766681

[40] Waddell G, Newton M, Henderson I, Somerville D, Main CJ. A Fear-Avoidance Beliefs Questionnaire (FABQ) and the role of fear-avoidance beliefs in chronic low back pain and disability. Pain. 1993;52(2):157–68. doi: 10.1016/0304-3959(93)90127-B 8455963

[41] Baer RA, Smith GT, Hopkins J, Krietemeyer J, Toney L. Using self-report assessment methods to explore facets of mindfulness. Assessment. 2006;13(1):27–45. doi: 10.1177/1073191105283504 16443717

[42] Dworkin RH, Turk DC, Wyrwich KW, Beaton D, Cleeland CS, Farrar JT, et al. Interpreting the clinical importance of treatment outcomes in chronic pain clinical trials: IMMPACT recommendations. The journal of pain: official journal of the American Pain Society. 2008;9(2):105–21. 10.1016/j.jpain.2007.09.005 18055266

[43] Stata 17 SaDS. StataCorp, College Station, Texas, USA 2021.

[44] Petitmengin C. What is it like to meditate? Methods and issues for a microphenomenological description of meditative experience. J Consciousness Studies. 2017;24(5–6):170–98.

[45] Petitmengin C, van Beek M, Bitbol M, Nissou JM, Roepstorff A. Studying the experience of meditation through Micro-phenomenology. Current opinion in psychology. 2018;28:54–9. doi: 10.1016/j.copsyc.2018.10.009 30502663

[46] Braun V, Clarke V. Reflecting on reflexive thematic analysis. Qual Res Sport Exerc. 2019;11(4):589–97.

[47] Braun V, Clarke V. One size fits all? What counts as quality practice in (reflexive) thematic analysis? Qual Res Psychol. 2021;18(3):328–52.

[48] Van Ryckeghem DML, Van Damme S, Vervoort T. Does attention bias modification training impact on task performance in the context of pain: An experimental study in healthy participants. PLoS One. 2018;13(7):e0200629. doi: 10.1371/journal.pone.0200629 30020983 PMC6051628

[49] Cohen M, Weisman A, Quintner J. Pain is Not a "thing": How That Error Affects Language and Logic in Pain Medicine. J Pain. 2022;23(8):1283–93. doi: 10.1016/j.jpain.2022.03.235 35427806

[50] Cohen M, Quintner J, van Rysewyk S. Reconsidering the International Association for the Study of Pain definition of pain. PAIN Reports. 2018;3(2):e634. doi: 10.1097/PR9.0000000000000634 29756084 PMC5902253

[51] Raja SN, Carr DB, Cohen M, Finnerup NB, Flor H, Gibson S, et al. The revised International Association for the Study of Pain definition of pain: concepts, challenges, and compromises. PAIN. 2020;161(9):1976–82. doi: 10.1097/j.pain.0000000000001939 32694387 PMC7680716

[52] van Hoorn S. Everything flows, or does it? Heraclitus on everything. antigonejournal.com: Antigone, an open forum for classics; 2021 [Available from: https://antigonejournal.com/2021/11/heraclitus-everything-flows/?t.

[53] Buswell REL D. S. The Princton Dictionary of Buddhism: Princton University Press; 2014.

[54] Wang Y, Qi Z, Hofmann SG, Si M, Liu X, Xu W. Effect of Acceptance versus Attention on Pain Tolerance: Dissecting Two Components of Mindfulness. Mindfulness (N Y). 2019;10(7):1352–9. doi: 10.1080/16506070902980703 31537989 PMC6752222

[55] Lu C, Moliadze V, Nees F. Dynamic processes of mindfulness-based alterations in pain perception. Front Neurosci. 2023;17:1253559. doi: 10.3389/fnins.2023.1253559 38027503 PMC10665508

[56] Ogden P, Minton K, Pain C. Trauma and the Body: A Sensorimotor Approach to Psychotherapy. WW Norton and Company, Inc, New York, NY. 2006.

[57] Petitmengin C, Lachaux JP. Microcognitive science: bridging experiential and neuronal microdynamics. Frontiers in human neuroscience. 2013;7:617. doi: 10.3389/fnhum.2013.00617 24098279 PMC3784800

[58] Lutz A, Jha AP, Dunne JD, Saron CD. Investigating the phenomenological matrix of mindfulness-related practices from a neurocognitive perspective. The American psychologist. 2015;70(7):632–58. doi: 10.1037/a0039585 26436313 PMC4608430

[59] Gelassenheit Heidegger M. Klett-Cotta, Stuttgart. 1959.

[60] Heidegger M. DISCOURSE ON THINKING; A Translation of Gelassenheit by John M. Anderson and E. Hans Freund UNIVERSITY OF FLORIDA LIBRARIES Internet Archives. 1959/1966;http://archive.org/stream/discourseonthink00heid/discourseonthink00heid_djvu.txt ((accessed 9/2/2013)).

[61] Damasio A. Mental self: The person within. Nature. 2003;423(6937):227. doi: 10.1038/423227a 12748620

[62] Craig AD. A new view of pain as a homeostatic emotion. Trends in neurosciences. 2003;26(6):303–7. doi: 10.1016/s0166-2236(03)00123-1 12798599

[63] Mehling WE, Hamel KA, Acree M, Byl N, Hecht FM. Randomized, controlled trial of breath therapy for patients with chronic low-back pain. Alternative therapies in health and medicine. 2005;11(4):44–52. 16053121

[64] Anderson BE, Bliven KCH. The use of breathing exercises in the treatment of chronic, nonspecific low back pain. Journal of sport rehabilitation. 2017;26(5):452–8. doi: 10.1123/jsr.2015-0199 27632818

[65] Arch JJ, Landy LN. Emotional benefits of mindfulness. 2015.

[66] Zeidan F, Vago DR. Mindfulness meditation-based pain relief: a mechanistic account. Annals of the New York Academy of Sciences. 2016;1373(1):114–27. doi: 10.1111/nyas.13153 27398643 PMC4941786

[67] Roberts RL, Ledermann K, Garland EL. Mindfulness-oriented recovery enhancement improves negative emotion regulation among opioid-treated chronic pain patients by increasing interoceptive awareness. J Psychosom Res. 2021;152:110677. doi: 10.1016/j.jpsychores.2021.110677 34801814

[68] Oliveira I, Vaz Garrido M, Carvalho H, Figueira Bernardes S. Sensing the body matters: profiles of interoceptive sensibility in chronic pain adjustment. Pain. 2023. doi: 10.1097/j.pain.0000000000003032 37768722

[69] Dillard A. On Seeing, chapter in: Pilgrim at Tinker Creek: New York: Harper’s Magazine Press; 1974.

[70] Seth AK. Interoceptive inference, emotion, and the embodied self. Trends in cognitive sciences. 2013;17(11):565–73. doi: 10.1016/j.tics.2013.09.007 24126130

[71] Hoskin R, Talmi D. Adaptive coding of pain prediction error in the anterior insula. European Journal of Pain. 2023. doi: 10.1002/ejp.2093 36799445

[72] Body Gadow S. and self: a dialectic. The Journal of medicine and philosophy. 1980;5(3):172–85. 6162903 10.1093/jmp/5.3.172

[73] Mehling WE, Wrubel J, Daubenmier JJ, Price CJ, Kerr CE, Silow T, et al. Body Awareness: a phenomenological inquiry into the common ground of mind-body therapies. Philosophy, ethics, and humanities in medicine: PEHM. 2011;6:6. doi: 10.1186/1747-5341-6-6 21473781 PMC3096919

[74] Mehling WE, Price C, Daubenmier JJ, Acree M, Bartmess E, Stewart A. The Multidimensional Assessment of Interoceptive Awareness (MAIA). PloS one. 2012;7(11):e48230. doi: 10.1371/journal.pone.0048230 23133619 PMC3486814

[75] Torous J, Lipschitz J, Ng M, Firth J. Dropout rates in clinical trials of smartphone apps for depressive symptoms: A systematic review and meta-analysis. J Affect Disord. 2020;263:413–9. doi: 10.1016/j.jad.2019.11.167 31969272

[76] Rintala A, Rantalainen R, Kaksonen A, Luomajoki H, Kauranen K. mHealth Apps for Low Back Pain Self-management: Scoping Review. JMIR Mhealth Uhealth. 2022;10(8):e39682. 36018713 10.2196/39682 PMC9463614

[77] Ulrich L, Thies P, Schwarz A. Availability, Quality, and Evidence-Based Content of mHealth Apps for the Treatment of Nonspecific Low Back Pain in the German Language: Systematic Assessment. JMIR Mhealth Uhealth. 2023;11:e47502. 37703072 10.2196/47502 PMC10534285

[78] Olsen MF, Bjerre E, Hansen MD, Tendal B, Hilden J, Hrobjartsson A. Minimum clinically important differences in chronic pain vary considerably by baseline pain and methodological factors: systematic review of empirical studies. J Clin Epidemiol. 2018;101:87–106 e2. doi: 10.1016/j.jclinepi.2018.05.007 29793007

[79] Sullivan MJ, Tripp DA. Pain Catastrophizing: Controversies, Misconceptions and Future Directions. J Pain. 2023. 10.1016/j.jpain.2023.07.004 37442401

[80] Rogers AH, Bakhshaie J, Ditre JW, Manning K, Mayorga NA, Viana AG, et al. Worry and rumination: Explanatory roles in the relation between pain and anxiety and depressive symptoms among college students with pain. J Am Coll Health. 2019;67(3):275–82. doi: 10.1080/07448481.2018.1481071 29979938 PMC12828677

[81] Zorn J, Abdoun O, Sonie S, Lutz A. Cognitive Defusion Is a Core Cognitive Mechanism for the Sensory-Affective Uncoupling of Pain During Mindfulness Meditation. Psychosom Med. 2021;83(6):566–78. doi: 10.1097/PSY.0000000000000938 33790200

[82] Mehling WE. Differentiating attention styles and regulatory aspects of self-reported interoceptive sensibility. Philosophical transactions of the Royal Society of London Series B, Biological sciences. 2016;371(1708). doi: 10.1098/rstb.2016.0013 28080970 PMC5062101

[83] Trevisan DA, Mehling WE, McPartland JC. Adaptive and Maladaptive Bodily Awareness: Distinguishing Interoceptive Sensibility and Interoceptive Attention from Anxiety-Induced Somatization in Autism and Alexithymia. Autism Res. 2021;14(2):240–7. doi: 10.1002/aur.2458 33336935

[84] Haller H, Breilmann P, Schroter M, Dobos G, Cramer H. A systematic review and meta-analysis of acceptance- and mindfulness-based interventions for DSM-5 anxiety disorders. Sci Rep. 2021;11(1):20385. doi: 10.1038/s41598-021-99882-w 34650179 PMC8516851

[85] Guu SF, Chao YP, Huang FY, Cheng YT, Ng HH, Hsu CF, et al. Interoceptive awareness: MBSR training alters information processing of salience network. Front Behav Neurosci. 2023;17:1008086. doi: 10.3389/fnbeh.2023.1008086 37025109 PMC10070746

[86] Evans DR, Eisenlohr-Moul TA, Button DF, Baer RA, Segerstrom SC. Self-Regulatory Deficits Associated with Unpracticed Mindfulness Strategies for Coping with Acute Pain. J Appl Soc Psychol. 2014;44(1):23–30. doi: 10.1111/jasp.12196 25843972 PMC4383260

[87] Britton WB. Can mindfulness be too much of a good thing? The value of a middle way. Curr Opin Psychol. 2019;28:159–65. doi: 10.1016/j.copsyc.2018.12.011 30708288 PMC6612475

[88] Rominger C, Schwerdtfeger AR. The misjudgment of interoceptive awareness: Systematic overrating of interoceptive awareness among individuals with lower interoceptive metacognitive skills. Conscious Cogn. 2023;117:103621. doi: 10.1016/j.concog.2023.103621 38113709

[89] Garland EL, Gaylord SA, Boettiger CA, Howard MO. Mindfulness training modifies cognitive, affective, and physiological mechanisms implicated in alcohol dependence: results of a randomized controlled pilot trial. Journal of psychoactive drugs. 2010;42(2):177–92. doi: 10.1080/02791072.2010.10400690 20648913 PMC2921532

[90] Bartos LJ, Posadas MP, Wrapson W, Krageloh C. Increased Effect Sizes in a Mindfulness- and Yoga-Based Intervention After Adjusting for Response Shift with Then-Test. Mindfulness (N Y). 2023;14(4):953–69. doi: 10.1007/s12671-023-02102-x 37090850 PMC10019420

[91] Balban MY, Neri E, Kogon MM, Weed L, Nouriani B, Jo B, et al. Brief structured respiration practices enhance mood and reduce physiological arousal. Cell Rep Med. 2023;4(1):100895. doi: 10.1016/j.xcrm.2022.100895 36630953 PMC9873947

