# Supplementary material for: Mind your pain: A single-arm feasibility study to assess a smartphone-based interoceptive attention training for patients with chronic low back pain
Source: PLoS One. 2024 Oct 24;19(10):e0307690. doi: 10.1371/journal.pone.0307690 (PMC11500934; doi:10.1371/journal.pone.0307690)
Supplement: S1 File — (DOCX) [file pone.0307690.s001.docx]

**S1 File: Mind your Pain: Study Protocol**

(As approved by UCSF IRB 20-322001

Title:

Mind your Pain: Validating the Mindful Interoceptive Exposure Task (MIET) for patients with chronic low back pain.

Department:

UCSF Family and Community Medicine; Osher Center for Integrative Health

San Francisco Veterans Affaires Medical Center

Investigators:

PI: Wolf Mehling, MD

Co-PI: Frederick Hecht, MD

Other investigators:

Irina Strigo, PhD

Wendy Hartogensis, PhD

Jeffrey Lotz, PhD

Clinical Research Coordinator:

Veronica Goldman, UCSF

Emily Murphy, SFVAMC

Project Summary:

*Mind your Pain (MyP),* also known as*Mindfulness-based Interoceptive Exposure Therapy (MIET*) is an innovative brief mindfulness-based task, developed within the frame of mindfulness-based cognitive therapy, and has been pilot tested in a small cohort of 15 patients with chronic musculoskeletal pain, the majority with cLBP, in Australia. It consists of an individual guided 1-hour introduction session and a 1 to 2-minute attention task subsequently performed several times per day over 12 weeks. The task is provided by a smart phone app and sent to participants as phone message reminders at up to 5 times per day: according to participant preference once in the morning and once at bedtime, as well as up to 3 additional times when participants are asked to use the app whenever they perceive the pain at its worst. The task is to focus on the most intense pain sensation in a detached and equanimous way and carefully observe potential changes in five aspects of that sensation: space (region/ borders/ immobile/ moving); sense of mass (heavy/ neutral/ light), temperature (cold/ warm/ hot/ neutral); density (dense/ solid/ loose/ constricted), and borders (diffuse/ sharp). This neutral sensory-descriptive interoceptive attention focus is aiming at preventing the learned aversive response to pain that entails ruminating thoughts, and negative affect rather than immediate sensory awareness. The pilot study in 15 chronic pain patients showed significant beneficial pre-post effects (Cohen’s *d*, ES) of 0.96 for pain anxiety, 0.86 for pain duration and 1.37 for pain intensity, maintained at 2-month follow-up. We offer the MyP intervention to patients with clearly defined chronic low back pain over 8 weeks, validate it with 30 participants on self-report key pain outcomes *and* objectively (using QST and fMRI**)**, who fit a low interoceptive awareness phenotype. We also use qualitative exit interviews.

Risk:

The study is considered of minimal risk.

Funding:

Funding is provided by NIH-NIAMS U19AR076737 (Overall PI: Jeffrey Lotz; directors of Bio-Behavioral Core: Wolf Mehling and Irina Strigo) under title: UCSF Core Center for Patient-centric Mechanistic Phenotyping in Chronic Low Back Pain (UCSF REACH)

Research Plan and Hypotheses:

This is an exploratory, mechanistic observational study with 30 patients undergoing 8-week pre-post assessment without a control group for proof-of-concept: can mindful interoceptive exposure possibly recover decreased interoceptive awareness AND dysfunctional central pain processing, thereby potentially reducing pain impact?

Hypotheses:

This is a proof-of-concept observational study without rigorous testing of hypotheses. Preliminary hypotheses are:

1) the new brief mindful interoceptive exposure task (MIET) is feasible and is acceptable to patients with chronic low back pain (cLBP) (Primary Outcome)

2) in cLBP patients with low interoceptive awareness (= below average scores on the Multidimensional Assessment of Interoceptive Awareness; MAIA), an 8-week mobile phone-based interoceptive exposure task can increase interoceptive awareness and recover dysfunctional central pain processing (assessed by quantitative sensory testing, QST; and fMRI connectivity) (Secondary Outcome)

3) exploratory hypothesis: Pain intensity (NRS) and pain interference (PROMIS-29) will improve from pre to post intervention.

4) exploratory hypothesis: Improvements in pain intensity and interference will be correlated with changes in MAIA scores, QST and fMRI connectivity.

Specific Aims:

| \| 1) to determine whether a mobile phone-based 8-week behavioral program of a brief mindful interoceptive exposure task (MIET) is feasible and acceptable to patients with cLBP.  2) to determine whether MIET can increase below-average interoceptive awareness (as assessed by the MAIA) in patients with cLBP. We will offer the MIET to the participants in the cohort studies of the BACPAC REACH study (U19) living in the Bay Area, applicants of the Intensive Pain Rehabilitation Program at the VA, and other patients with cLBP, and validate it on self-report outcomes (pain intensity/ interference) *and* objectively (QST and fMRI**)**with 30 participants over 8 weeks, who fit a low interoceptive awareness phenotype.  3) To assess potential changes in chronic pain-related attitudes, believes and coping styles secondary to the intervention. In addition to psychological questionnaires, we will use qualitative exit interviews in 20 selected participants to explore phenomenological differences in patients' attention styles that may be associated with benefits for pain scores.  4) to assess whether potential changes in self-reported pain outcomes and/or objective QST measures are correlated with potential changes in interoceptive awareness (MAIA). All 30 participants will undergo pre-post assessment of pain intensity (NRS), pain interference (PROMIS-29), and QST.  5) to assess whether changes in self-reported interoceptive awareness (MAIA) are associated with changes in brain activity and connectivity. All 30 participants will undergo pre-post assessment of the MAIA and the fMRI Anticipation/Attention task. \| \| --- \| |
| --- | --- |

Design:

This is an uncontrolled, exploratory, mechanistic observational pilot study without control group for the validation of a behavioral task in individuals with chronic pain.

All subjects undergo:

1) questionnaire tests

2) psychophysical testing of cutaneous thermal sensitivity. Using previously validated procedures, we will examine each participant's sensitivity to temperature stimulation.

3) Functional Magnetic Resonance Imaging (fMRI) on the brain.

4) daily pain assessments by electronic momentary assessments (EMA) over 8 weeks

Background and Significance:

Chronic low back pain (cLBP) is the number one reason for disability in the US and a major burden for individuals and public health. It is a leading medical diagnosis associated with the opioid epidemic. The lack of understanding of its underlying mechanisms, of potential discernible phenotypes, and of appropriately targeted therapies that generally have shown only limited benefits for these patients is the rationale for a major NIH initiative (HEAL and BACPAC), for which UCSF has received a large U19 grant (PI Jeff Lotz) conducting two observational cohort studies. The U19 includes a biobehavioral core (Directors Dr. Mehling and Dr. Strigo) and one small proof-of-concept study that aims to validate an innovative behavioral approach to chronic pain using mindful interoceptive exposure. For this approach, a task has been developed by a behavioral therapist in Australia. By now, two publications have been published by the developer in peer-reviewed journals, which (a) showed benefits for patients with chronic pain^1^ and  (b) improved pain tolerance in healthy volunteers^2^.

This new behavioral task--if shown valid, feasible and acceptable to a US population with cLBP--implies a potential paradigm shift in the management of chronic pain. Most pain management encourages distraction from pain, whereas this approach attempts to facilitate a mindful, equipoised, neutral, non-evaluative attention style of immediately sensing pain rather than thinking/ruminating/worrying about pain. This may have major implication for future pain management.

^1^ Cayoun B, Simmons A, Shires A. Immediate and lasting chronic pain reduction following a brief self-implemented mindfulness-based interoceptive exposure task: a pilot study. Mindfulness (N Y). 2017;https://doi.org/10.1007/s12671-017-0823-x.

^2^ Shires A, Sharpe L, Newton JTRO. The relative efficacy of mindfulness versus distraction: The moderating role of attentional bias. Eur J Pain. 2019 Apr;23(4):727-738. doi: 10.1002/ejp.1340. Epub 2018 Dec 4.

Preliminary Studies:

*Mindfulness-based Interoceptive Exposure Therapy (MIET*) is an innovative brief mindfulness-based intervention developed within the frame of mindfulness-based cognitive therapy that has been pilot tested in a small cohort of 15 patients with chronic musculoskeletal pain, the majority with cLBP^1^. It consists of an individual guided 1-hour introduction session and a 1-minute attention task subsequently performed several times per day over 12 weeks. The task is to focus on the most intense pain sensation in a detached and equanimous way, and carefully observe potential changes in five aspects of that sensation: space (region/ borders/ immobile/ moving); sense of mass (heavy/ neutral/ light), temperature (cold/ warm/ hot/ neutral); density (dense/ solid/ loose/ constricted) and borders (diffuse/ sharp). This neutral sensory-descriptive interoceptive attention focus is aiming at preventing the learned aversive response to pain that entails ruminating thoughts, and negative affect rather than immediate sensory awareness. This small pilot study conducted by MIET's developer showed significant beneficial pre-post effects (Cohen’s *d*, ES) of 0.96 for pain anxiety, 0.86 for pain duration and 1.37 for pain intensity, maintained at 2-month follow-up.^1^


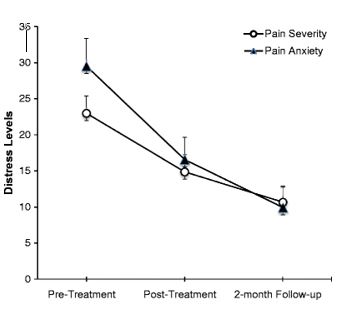


In a 3-arm RCT in 100 healthy participants exposed to cold pressure experimental pain while practicing either MIET or a distraction task, the MIET group outperformed two control groups on pain outcomes (Cohen’s *d*= 1.06/1.42).^2^

^1^: Cayoun B, Simmons A, Shires A. Immediate and lasting chronic pain reduction following a brief self-implemented mindfulness-based interoceptive exposure task: a pilot study. Mindfulness (N Y). 2017; https://doi.org/10.1007/s12671-017-0823-x.

^2^: Shires, A, Sharpe, L, Newton John, TRO. The relative efficacy of mindfulness versus distraction: The moderating role of attentional bias. *Eur J Pain*. 2019; 23: 727– 738. [**https://doi.org/10.1002/ejp.1340**](https://doi.org/10.1002/ejp.1340)

Procedures:

**Screening, consent, baseline questionnaires:** Potential participants will be informed about the study and screened for preliminary eligibility by phone by a clinical research coordinator (CRC) at the UCSF Osher Center. The screening will include a verbal consent to be screened and follow a screening script. Once deemed preliminarily eligible, potential participants will receive a study brochure, the consent forms by mail (with postmarked return envelope) and an invitation to schedule a further discussion by zoom with either the CRC or the PI. Recruitment and screening will occur by phone or Zoom from the UCSF Osher Center.

After signing informed both consent forms (One overall UCSF consent; one QST and fMRI-specific VA consent), they will answer questionnaires on-line at home (Qualtrics). If final eligibility is confirmed according to questionnaire answers, eligible participants will be informed and will be scheduled for quantitative sensory testing (QST) and fMRI at the San Francisco Veterans Affairs Medical Center (SFVAMC; Visit 1). The part of the study conducted at the SFVAMC will include phone calls and will not include any communication or screening using Zoom.

Subjects will be asked to provide an email address on the Informed Consent form to receive the questionnaire online via Qualtrics, an online research survey software with a securely built platform, widely used by academic institutions. The participants will be provided a unique subject ID and password and will be sent an email containing the Qualtrics link or a paper survey as an alternative option to be used for accessing the questionnaires. The questionnaires will take **45 - 60 minutes**.

**Visit 1**: A SRA at the SFVAMC will conduct the QST and fMRI. This will take **2-4 hours**.

All procedures done in Visit 1 have previously been approved (Dr. Strigo: #14-13833; 19-28942)

**1. Sensory Function Testing (~30 minutes):**Subjects will undergo psychophysical testing of their cutaneous thermal sensibility. Using procedures similar to the ones Dr. Strigo has previously used in other IRB-approved protocols, we will examine each patient's sensitivity to cool, warm, cold, and heat. Stimuli will be applied with TSA-II or Pathway NeuroSensory Analyzer (Medoc. Ramat Yishai, Israel) and by submerging participants' hand into a cold circulating water bath. Stimulation paradigms will involve presentation of several non-painful and moderately painful stimuli; both hot and cold temperatures (0-50C, 32-122F) will be applied to the skin for short periods of time (<90 sec). None of these temperatures can damage the skin when applied for short periods of time, as in this study and previous studies. Subjects are asked to rate stimulus on visual analogue scales (VAS) for temperature, pain intensity, and pain unpleasantness in order to construct stimulus-response functions and determine appropriate temperatures to use in the fMRI session. Sometimes subjects are also asked to perform a simple cognitive task that requires subjects to respond via button press to mouse button in response to various visual and auditory stimuli that are presented on a computer screen and through the headphones, respectively. Audio stimuli would consist of mainly speaking with study staff to ensure subject comfort and comprehension, as well low or high pitch sounds/tones corresponding with visual stimuli. Any sounds/tones will be non-invasive, non-abrupt sounds in order to avoid anxiety producing stimuli. Visual stimuli will include different colored crosses or shapes, or IAPS (International Affective Picture System) images, previously used in another study by the Dr. Strigo (#14-13833; 19-28942). Subjects are also instructed to withdraw at any time if the stimulus is too uncomfortable to tolerate.

**- Heat Pain Threshold Testing:** To measure heat pain thresholds, the study team will use the thermode to apply heat ranging from 32F to 122F (0-50C) for short periods of time (<90 sec). After each temperature, participants will be asked to point to a visually representative scale and rate the following: the maximum sensation of pain felt (if the stimulus is painful), the maximum sensation of warmth felt (if the stimulus is not painful), and the maximum unpleasantness of each pulse. Based on ratings, the research team will choose different temperatures to use in the scanning 8.5

**- Pressure Pain Sensitivity**: will be assessed using an analog algometer with a 1-cm^2^rubber probe (FPK20, Wagner Instruments, Greenwich, CT, USA) to quantify pressure pain thresholds (PPT).  The primary test site will be located in the lumbar region by the participant’s response to manual over-pressure (springing palpation) performed in the prone position.  The control site will be located over the contralateral trapezius muscle (diagonal from lumbar site).  Pressure will be manually increased at a rate of rise of 0.5 kgf/cm^2^/s (10 kg max, metronome guided) until participants first report that the pressure sensation becomes painful.  Pressure intensity (in kgf/cm^2^) read from the algometer at that time is considered the PPT.  Measurements will be conducted 3x/site with 60-s rest intervals between each pressure application.  Probe placement will be varied slightly trial to trial to prevent sensitization from repeated testing of the same site.  Mean PPT will be used for analysis. Subject are allowed to end participation if their perceived pain from the procedures is too great to tolerate.

**- Onset and Offset Analgesia Paradigm:**To elicit offset analgesia, the Medoc thermode will deliver a short 3-temperature train protocol, which involves warming the skin for approximately 5-30 seconds and finally returning to the first temperature (T3) for approximately 20 seconds (Grill and Coghill, 2002; Yelle et al., 2008), or the inverse of this sequence. Between the approximately 30 second train, the thermode is held at a constant temperature for at least 90 seconds. Subject are allowed to end participation if their perceived pain from thermal procedures is too great to tolerate.

**- Thermal Grill Testing:** Thermal stimuli will be presented with a specially constructed thermode consisting of interlaced copper tubes. Two separate electronic drivers control the temperatures of the tubes, so that odd and even numbered tubes can be maintained at different temperatures. The thermode will be used in three different stimulation modes. It will be uniformly a) heated to approximately 40C and 20C (thermal grill stimulus, see [Craig and Bushnell, 1994]^4^ for details of thermal grill procedures). Subject are allowed to end participation if their perceived pain from thermal procedures is too great to tolerate.

**- Temporal Summation of Heat Pain Paradigm:**To test whether abnormal response to temporal summation of pain responds to repeated exposure therapy, we will use a modification of the discriminating test provided by the repeated brief-contact heat paradigm of Vierck et al. (1997)^5^. A series of brief-contact heat stimuli will be applied. In this test, a hot probe repeatedly contacts the skin for brief periods of time with the interstimulus intervals (ISI). To measure the degree of temporal summation, subjects will be asked to rate the first and the last stimulus within each train of stimuli using the intensity and unpleasantness scales. Subjects may also continuously rate their subjective pain experience using the electronic VAS (Medoc, Israel). To measure the recovery from repeated heat stimulation, subjects may also be asked to rate their pain and unpleasantness intermittently after the offset of heat stimulation. Subject are allowed to end participation if their perceived pain from thermal procedures is too great to tolerate.

**- Temporal Summation of Pressure Pain Paradigm:**measures increases in excitatory pain pathways and is thought to reflect the progressive increase in dorsal horn neuronal firing in response to repetitive C-fiber stimulation.^1-4^ Enhanced temporal summation is common in chronic pain and is predictive of pain outcomes.^5,6^ We will evaluate temporal summation in triplicate using a 40 g Neuropen Neurotip (Owen Mumford, Oxfordshire, United Kingdom) applied to the skin of volar forearm and lumbar region, followed a train of 10 identical stimuli (1 Hz) using a metronome for timing. Following the single stimulus and the train of 10 stimuli, participants will report the pain intensity of the pinprick sensation using a 0-100 numerical rating scale (NRS; 0 = no pain, 100 = worst pain imaginable).  The palmer forearm and lumbar region will each be tested three times.  Temporal summation for each site will be calculated as the mean difference in pain ratings evoked by the single stimuli and the trains of stimuli, with the subject rating the 1st and 10^th^ stimulus.  Participants will also rate any ongoing *pain aftersensations* at 15- and 30-s following the final train of stimuli. Subject are allowed to end participation if their perceived pain from thermal procedures is too great to tolerate.

**- Conditioned Pain Modulation Paradigm:**Conditioned pain modulation (CPM) is a robust psychophysical phenomenon in which a pain in one area (test stimulus) of the body is reduced with an additional pain stimulus applied to a different part of the body (Nir and Yarnitsky, 2015)^6^. Subjects may be asked to place the nondominant arm in a water bath, set to an uncomfortably cold or hot temperature. At the end of immersion, pain scores will be obtained. After a rest period, subjects immerse one arm in the water bath followed by the heat stimulus. Conditioned pain modulation (CPM) manifests as decreased pain ratings of the dominant arm heat stimulus while the nondominant arm is in the water bath. Subject are allowed to end participation if their perceived pain from thermal procedures is too great to tolerate.

**2. MRI Scanner (~1 hour):**Subjects will be scanned using the 3T Siemens MRI scanner at the SF VAMC in building 203 room BA-30. Scans will include sensory testing, where skin stimulation will be on the lateral forearm using the same MRI-compatible temperature probe described above. Sensory stimuli will be presented in the same fashion as outlined above, and audiovisual stimuli may also be presented during the scan. Sensory and audiovisual stimuli paradigms have been used in another study by Dr. Strigo (#14-13833). Subjects will undergo practice tasks, similar to the paradigms in the scanner, before the scan to ensure that they understand the tasks that will be completed while in the scanner.

When scheduling, participants are reminded to bring food and water unless they would like to purchase meal items from the SFVAMC cafeteria.

*Contacts for future VA research within our research program:* Subjects will be asked for permission to contact them about future VA research within our laboratory. Their contact information will not be used for future research until such time that an SOP is developed and approved by the IRB and VA R&DC per VHA Handbook 1200.12.

**Visit 2 (~ 1 hour)**: Participants will be scheduled for a 1-hour zoom session with the PI (Dr. Mehling), who will explain the MIET, answer all questions, and will teach the task to the participants. The Task is an attention exercise for the back pain to be practiced 3 to 5 times a day for 8 weeks. The Task is conducted based on a Qualtrics phone inquiry, triggered at 2 specific time points by SMS and self-triggered up to 3 times a day by the participants whenever the pain is at its worst.

**Phone/Zoom contacts (~10-30 minutes each for 7 calls)**:  Once a week after the initial training, the SRA will contact the participants by phone or zoom and go over any potential difficulties and barriers. If participants encounter difficulties or have questions, they are always able to leave a voice mail with the SRA for requesting a call back.

**Visit 3 (~ 1 hour)**: Two weeks after the initial training, participants will have another 1-hour zoom session with the PI to go over the experience of the last 2 weeks and discuss potential adjustments if needed.

**Visit 4 (2 to 4 hours)**: Eight weeks after the initial training session, participants will be scheduled for the follow-up QST and fMRI at the VA. Both QST and fMRI will be the same as in Visit 1. Communication from the SFVAMC will be done by phone and not include any video calls by Zoom.

**Repeat Questionnaires (~45-60 minutes)**: Participants will answer questionnaires online (Qualtrics) or on paper. Except demographics and social background questions, the questionnaires are the same as at baseline.

**Potential Visit 5 - Qualitative Interviewing (2 hours):** 20 of the participants will be invited for a follow-up 2-hour interview with Dr. Mehling for (a) debriefing and (b) eliciting details about the phenomenology of the participants' attention style^3^ during the task performance of the past 8 weeks. The20 interviews will be in selected participants, 10 with strong (upper tercile) and 10 with weak (lower tercile) change scores in pain impact outcomes. This interview will be conducted in person if the COVID-19 situation allows, otherwise the interviews will use the Zoom platform.
^3^ Petitmengin C, Lachaux JP. Microcognitive science: bridging experiential and neuronal microdynamics. Front Hum Neurosci. 2013;7:617. PubMed PMID: 24098279; PubMed Central PMCID: PMCPMC3784800.

^4^ Craig AD, Bushnell MC (1994) The thermal grill illusion: unmasking the burn of cold pain. Science 265:253-55. Available at <https://doi.org/10.1126/science.8023144>

^5^ Vierck Jr. CJ, Cannon RL, Fry G, Maixner W, Whitsel BL (1997) Characteristics of temporal summation of second pain sensations elicited by brief contact of glabrous skin by a preheated thermode. J Neurophysiology 78:992-1002

^6^ Nir R, Yarnitsky D (2015) Conditional pain modulation. Curr Opinions Suppot Palliat Care 9:131-7

Questionnaire Instruments:

All instruments have been published. In parenthesis: (number of items), in brackets: *[reference],* measures used in REACH or IPRP studies are marked with *.

1) BACPAC Baseline Demographics*Includes HEAL required questions*(22)* only at baseline. This includes questionnaire items necessary to  adjust for MRI, e.g. to fit MRI-compatible correcting eye glasses.

2) PEG scale assessing pain intensity and interference (Pain, Enjoyment, General Activity) (3)*

3) Generalized Anxiety Disorder (GAD-2)*[Kroenke et al, 2007]*(2)*

4) Patient Health Questionnaire-2 (PHQ-2)*[Kroenke et al, 2003*[**(2)**](https://iris.ucsf.edu/Study_App.jsp?FORM_MODE=EDIT&tab=full&s=1701923559657#_ENREF_2)*

5) Tobacco, Alcohol, Prescription medication, and other Substance use (TAPS) tool (4)*

6) PROMIS Physical Functioning Short Form 6b (2 items not in PROMIS-29)*

7) PROMIS Sleep Disturbance 6a*[Yu et al, 2011]*(2 items not in PROMIS-29)*

8) Sleep Duration Question*[Kurina et al, 2013]*(1)*

9) Pain Catastrophizing Scale - short form 6 (PCS)*[McWilliams, 2015]*[**(6)***](https://iris.ucsf.edu/Study_App.jsp?FORM_MODE=EDIT&tab=full&s=1701923559657#_ENREF_6)

10) Patient Global Impression of Change (PGIC) (1) only at 8 weeks.

11) PROMIS-29+2 Profile v2.1 (PROPr) 02Jan2020 *[PROMIS]*(31)*

12) Charlson Comorbidity Index (CCI), *[MDCalc (*[*https://www.mdcalc.com/charlson-comorbidity-index-cci*](https://www.mdcalc.com/charlson-comorbidity-index-cci)*)]** baseline only
13) PainDETECT Questionnaire (PD-Q)*[Freynhagen et al., 2006]*(7)*

14) Fear-Avoidance Beliefs Questionnaire Physical Activity (FABQ-PA)*[Waddell et al., 1993]*(5)*

15) Chronic Pain Acceptance Questionnaire-SF8*[Fish et al. 2010]*(8)*

16) Multidimensional Assessment of Interoceptive Awareness (MAIA)*[Mehling et al., 2018]*(37)

17) Perceived Stress Scale*[Cohen et al., 1989]*(4)*

18) International Positive (and Negative) Affect Schedule 10-item SF*[Watson et al., 1988]*(5)*

19) PROMIS Emotional Support 4a V2*[Tucker et al, 2014]*(4)* only baseline

20) Pain Self-Efficacy Questionnaire 4-item version (items 4, 6, 8, 9)*[Chiarotto et al, 2016]*(4)*

21) Primary Care PTSD Symptom Screener*[Prins et al, 2016]* (1)* only baseline

22) Financial Strain*[Puterman et al, 2012]*(1)* only baseline

23) Perceived Discrimination *[NIMDH]*(1)* only baseline

24) Expectation of Pain Relief*[Cormier et al, 2016]*(1)* only baseline

25) Pain Anxiety Symptoms Scale - Short Form (PASS-20) - (items 6-10, 16-20)*[McCracken et al. 2002]*(10)*

26) Five Facets Mindfulness Questionnaire (FFMQ) *[Baer et al. 2006]*(39)

27) CHILD TRAUMA QUESTIONAIRE (CTQ) – SHORT FORM [Bernstein et al.1995]

28) COLUMBIA-SUICIDE SEVERITY RATING SCALE (C-SSRS) [Posner et al. 2011]

Analyses:

**Overall Analysis Approach:**Preliminary analysis will be performed to confirm that key data variables are clean and complete. We will use sex and age as covariate, as insula function varies across sex and age. The principal analysis will use 'intent-to-treat' methods, in which all observations will be included for individuals, regardless of adherence to the protocol. As a secondary analytic method, we will also perform as-performed analyses with those who respond and enter data to at least 70% of phone prompts.

| **Table 1: Feasibility and acceptability assessment** | |
| --- | --- |
| **Feasibility Questions** | **Feasibility Measures (benchmark metrics)** |
| Can I recruit my target population? | Number screened (10 per month); number enrolled: (5 per month). *We will assess the number of enrolled participants from each outreach method, identify and address specific barriers to recruitment, modify emphasis on recruitment where needed, and use results to plan recruitment for subsequent studies.* |
| Can I keep participants in the study? | Retention rates for study measures (≥80% completion). *If target is not met, we will use feedback from participant interviews to modify retention steps.* |
| Are the assessments too burdensome? Can we complete outcome assessment? | Proportion of planned pre- and post-intervention assessments completed (≥80%), we will assess duration of assessments with quantitative and qualitative participant feedback. *If targets are not met, we will consider ways to shorten assessments.* |
| Is MIET acceptable to participants? | Acceptability ratings (≥8 on a 0-10 scale for ≥80% of participants); we will add qualitative participant assessments and document reasons for dropouts. *If target is not met, we will use feedback from participant interviews to modify intervention components* |
| Are phone based task and assessment of pain intensity/interference feasible? | Proportion of EMA assessments responded to (≥70%). Qualitative data on acceptability.*If target is not met, we will use feedback from participant interviews to modify EMA assessment procedures* |

**Statistical Design and Power**: The primary purpose of this study is to determine feasibility and validate a novel mindfulness and interoceptive exposure-based intervention (MIET) in a subtype/phenotype of cLBP patients with low (below average) interoceptive awareness to determine initial clinical utility as a basis for future research. To achieve this, we will offer the MIET to the participants in the UCSF REACH Clinical Core cohort and the IPRP program at the SFVAMC, validate it on self-report outcomes (pain impact) *and*objectively (QST and fMRI Anticipation/Attention task à BBC proposal**)** with 30 participants over 8 weeks, who fit a low interoceptive awareness phenotype. Statistical power was estimated based on feasibility to find significant changes in disability outcomes (primary outcome, pain impact at 8 weeks.

In a very first pilot study of MIET in Australia participants experienced a significant reduction in pain intensity with a standardized effect size of *d*=1.37.1 This report may be overly optimistic. Assuming 10% attrition, we would be able to detect a ES *d*=0.78 (two-tailed; alpha 0.05, beta 0.2). For these preliminary estimations of the effect size, we used power calculations on the basis of the normal approximation with a 5% significance level for a 2- sided test, we determined that a sample size of 30 patients would provide adequate power (.70–.93) to detect minimal clinically important change. As this is a proof-of-concept study rather than an efficacy study, we will primarily assess for standardized effect sizes (Cohen’s *d*with 95% confidence intervals).

**Analysis of fMRI data:** All structural and functional image processing will be done with the Analysis of Functional Neuroimages (AFNI) software package (or similar) as has been done before by Dr. Strigo (e.g., Strigo et al., 2013, 2014)^7,8^. Descriptive statistics [M(SD)] of different groups and between group comparisons will be used. All structural and functional image processing (fMRI) and analysis will be performed with the Analysis of Functional Neuroimages (AFNI), FSL, ANTS or similar software packages (Cox, 1996). In order to minimize motion artifact, exoplanar images will be realigned to individually chosen base scan. Additionally, data will be time-corrected for slice acquisition order. Time series data for each individual will be analyzed using a multiple regression model. Data of each subject will be normalized to Talairach coordinates (Lancaster et al., 2000) and a whole-brain mask will be applied to screen out non-brain voxels and voxels falling within the artifact region. The voxel-wise percent signal change data will be entered into a linear mixed effects model or ANOVA with condition as a fixed factor and subjects as a random factor. A threshold/cluster method will then be applied, and the average percent signal difference extracted from regions of activation found to survive this threshold/cluster method.

***Task-based fMRI:***Preprocessed data will be further analyzed with Analysis of Functional NeuroImages (AFNI) software package and optimized based on HCP recommendations. A multiple regression model corrected for autocorrelation consisting of regressors for anticipation and stimulation will be applied to preprocessed time-series data for each individual. For multivariate analysis, a separate regressor will be calculated for each anticipation and stimulation trial such that each event has its own estimated amplitude. To reduce the false positives induced by cross-correlations, time-series data will be fit using the AFNI program 3dREML or 3dLSS to improve classification accuracy.

*Activation maps will be created on a single-subject basis*. Masks of selected ROIs will be created in MNI space using AFNI and will be applied to the functional activation maps using 3dDeconvolve. Using a separate AFNI program, 3dROIstats, the mean activation will be extracted and T-statistics will be calculated in each region to improve classification accuracy.

*Single-subject Functional Analysis of Regional Activation Maps*: Average activation within each region will be subjected to regression analysis by way of LASSO. The LASSO regression model will be executed in R using the glmnet package for Lasso and Elastic-Net Regularized General Linear Models. LASSO will be performed on a single-subject basis to create subject specific models that best separate conditions of interest (e.g. high pain anticipation from low pain anticipation). Logistic regression methods such as LASSO allow for a smaller number of predictors to be included in the model, allowing the experimental pain task to be shortened and optimized. Regression will be then fit to the training set and will be optimized by cross-validating the model 100 times. This will allow for accurate and consistent discrimination between single-subject neurobiological patterns of different conditions. We can then apply these LASSO predictions on the test set (e.g., anticipation during uncertainty) at a probabilistic level, based on the correlation of the activation statistic to the cross-validated glmnet model. Thus, each participation can be labeled individually on the condition of interest.

**Qualitative Exit Interviews:**In-depth qualitative semi-structured interviews will be conducted after the final assessment. We will use the micro-phenomenology method by Petitmengin^3^ and probe the first-person experience in managing pain by either the narrative or the interoceptive awareness approach. This method has been developed to capture subjective processes in meditation and consciousness research (directed by Francisco Varela). It attempts to combine and mutually enlighten neural and experiential descriptions of cognitive processes and holds promise for providing additional heuristic information on the micro-phenomenology of interoceptive awareness and mind-body approaches in general. We will conduct 20 elicitation interviews in selected participants, 10 with strong (upper tercile) and 10 with weak (lower tercile) changes in pain impact outcomes.

References:

^1^: Cayoun B, Simmons A, Shires A. Immediate and lasting chronic pain reduction following a brief self-implemented mindfulness-based interoceptive exposure task: a pilot study. Mindfulness (N Y). 2017; https://doi.org/10.1007/s12671-017-0823-x.

^2^: Shires, A, Sharpe, L, Newton John, TRO. The relative efficacy of mindfulness versus distraction: The moderating role of attentional bias. *Eur J Pain*. 2019; 23: 727– 738. [**https://doi.org/10.1002/ejp.1340**](https://doi.org/10.1002/ejp.1340)

^3^:  Petitmengin C, Lachaux JP. Microcognitive science: bridging experiential and neuronal microdynamics. Front Hum Neurosci. 2013;7:617. PubMed PMID: 24098279; PubMed Central PMCID: PMCPMC3784800.

^4^: Craig AD, Bushnell MC (1994) The thermal grill illusion: unmasking the burn of cold pain. Science 265:253-55. Available at https://doi.org/10.1126/science.8023144

^5^: Vierck Jr. CJ, Cannon RL, Fry G, Maixner W, Whitsel BL (1997) Characteristics of temporal summation of second pain sensations elicited by brief contact of glabrous skin by a preheated thermode. J Neurophysiology 78:992-1002

^6^: Nir R, Yarnitsky D (2015) Conditional pain modulation. Curr Opinions Suppot Palliat Care 9:131-7

^7^: Strigo IA, Matthews SC, Simmons AN (2013) Decreased frontal regulation during pain anticipation in unmedicated subjects with major depressive disorder. Transl Psychiatry 3:e239 Available at: http://www.pubmedcentral.nih.gov/articlerender.fcgi?artid=362914&tool=pmcentrez&rendertype=abstract

^8^: Strigo IA, Spadoni AD, Lohr J, Simmons AN (2014_ Too hard to control: compromised pain anticipation and modulation in mild traumatic brain injury. Transl Psychiatry 4:e340 Available at: <http://www.pubmedcentral.nih.gov.articlerender.fcgi?artid=3905226&tool=pmcentrez&rendertype=abstract>

Sample Size Justification:

Sample size was deemed sufficient for a proof-of-principle pilot study.

For the statistical power: see Analysis section above

Inclusion Criteria:

1. Chronic low back pain (cLBP) defined according to the NIH Research Task Force recommendation on Research Standards for cLBP: pain at least half the days in the past 6 months, by using 2 questions and a human figure drawing illustrating the region as the space between the lower posterior margin of the rib cage and the horizontal gluteal fold.

2. Average pain in the last month at least 3 out of 10 on Numeric Rating Scale [range 0 – 10, for 0 signifying no pain and 10 signifying worst pain imaginable]. This level of pain allows comparability of the study results with the majority of cLBP studies. Pain rated less than 3 is too mild to detect improvement.

3. Men and women aged 18-65 years old. We are not enrolling younger children as they are not part of the Intensive Pain Rehabilitation Therapy program. 65 is our upper limit for age due to changes in blood flow on the MRI.

4. Eligibility will be assessed using the following questions: “(1) How long has back pain been an ongoing problem for you? and (2) How often has low-back pain been an ongoing problem for you over the past 6 months?” A response of greater than three months to question 1, and a response of “at least half the days in the past 6 months” to question 2 would meet the cLBP eligibility criterion.

5. Ability to speak English. We do not have the capacity, given the resources available in this proposal, to translate all course material and conduct groups into another language. We have previously enrolled Hispanic participants into other studies who were fluent in English, and expect to do this in the proposed study.

6. Low level of interoceptive awareness and habitual distraction as coping mechanism with pain. This is defined as:

a) MAIA summary score below the population mean score of 3.41.  The value of 3.41 is the mean value of a sample of primary care patients at Kaiser Permanente in a prior study.

b) the MAIA Non-Distraction score is below 2.91 [possible range 0-5].  The value of 2.91 is the mean value plus standard deviation in the same sample.^4^

This eligibility criterion was chosen to test the hypothesis that the MIET task will be able to increase interoceptive awareness in patients with chronic low back pain with below average interoceptive awareness and preference for distracting themselves from their pain experience.

7. Owning a smart phone: the task is smart phone-based.

Under NIH definition, all individuals under the age of 21 are considered children. Eligible participants who are 18 years of age or older will be recruited for this study. Younger children will not be included in this study because a pediatric sample may require different intervention approaches.

Exclusion Criteria:

1. Unable to provide informed consent.

2. A substance abuse, mental health, or medical condition that, in the opinion of investigators, will make it difficult for the potential participant to participate or that may need immediate changes in medical management that will affect study outcome measures. Such conditions may include cancer, liver failure, renal failure, pain conditions from inflammatory diseases (e.g. rheumatoid arthritis, ankylosing spondylitis, lupus), malignancies or abdominal aortic aneurysm, muscle weakness from radiculopathy. Radiculopathy or sciatic pain is NOT excluded as long as the condition is stable and does not lead to significant movement restrictions or <4/5 muscle weakness. Persons with significant substance abuse or mental health conditions that interfere with social functioning may be disruptive. Oher medical or mental health conditions that need immediate changes in management need to be addressed before starting the study so that more reliable baseline measurements can be made. Patients who may need assessment for potentially necessary surgical interventions may not be able to complete the study. Regular opioid prescription is not an exclusion if stabile over the past 3 months.

3. Spine related current or history of spine infection, spine tumor, vertebral fracture, cauda equina syndrome. Colorblindness or left handedness. Conditions would increase heterogeneity of the sample.

4. Blindness, severe vision problems, deafness, severe hearing problems, bipolar or manic depression and not taking medication, major depression, psychoses (major), a substance abuse condition, dementia, unable to get up and down from the floor. Condition might make it difficult to participate.

5. Some other serious medical conditions that may alter key study outcomes, including untreated hypothyroidism, renal failure, and cirrhosis. Conditions that may alter key study outcomes.

6. Involvement in a lawsuit related to their back. Complicated medico-legal issues that could lead to individuals having a financial incentive to not report improvement.

7. Involved in Worker's Compensation claim.

8. Pregnant, breast-feeding, or planning to get pregnant in the next 12 months or less than 3 months post-partum. Particular back problems than may be associated with pregnancy and delivery may confound study outcomes.

9. Lack of stable housing or plan to move out of the area within the next 6 months.

10. MRI-related exclusion criteria: Cardiac pacemaker, metal fragments in eyes/skin/body (shrapnel), subjects who have ever been a metal worker/welder; history of eye surgery/eyes washed out because of metal, aortic aneurysm clips, prosthesis, by-pass surgery/coronary artery clips, hearing aid, heart valve replacement, subjects with an I.U.D, a shunt (ventricular or spinal), electrodes, metal plates/ pins/screws/wires, or neuro/bio-stimulators (TENS unit), vision problems uncorrectable with lenses, claustrophobia; inability to lie still on one’s back for approximately 60 minutes; prior neurosurgery; older tattoos with metal dyes; unwillingness to remove nose, ear or face jewelry, braces or permanent dental retainers. Iron-containing metal parts in the body can potentially be dislocated by strong magnetic fields and preclude assessment with MRI.

11. Received a steroid or botox injection in or near the spine in the last 3 months. This may alter key study outcomes.

Subject Identification Method:

We plan to recruit both males and females. We anticipate that male/female ratio will match the gender, racial and ethnic composition of San Francisco county population.

at UCSF: UCSF saw 1,658 patients for cLBP in primary care during the past two years (data search 5/2017). The PIs  Dr Mehling and Dr Strigo are co-investigators in the NIH-funded BACPAC REACH study at the UCSF Spine Center. This study plans to enroll an internet cohort of 5,000 patients. Every 2 weeks, the REACH study manager will provide the SRA of the Mind your Pain study with a list of enrollees, which live in the Bay Area. Enrollees have consented to be contacted for other back pain-related studies. They will be invited by mail and e-mail and receive a flyer about the Mind your Pain (MyP) study with contact phone number and e-mail address.

at the VA: Potential participants will be recruited from the Intensive Pain Rehabilitation Program (IPRP), a clinical service offered to veterans with chronic pain at the SFVAMC. In addition, we will identify potentially eligible participants enrolled in research studies with Dr. Strigo at the SFVAMC. For another study, the IPRP program is an established clinical program (Clinical Director Sarah Palyo, Ph.D. is a co-investigator on the proposal) serves about 500 patients every year. If patients enrolled in IRPR have to wait for 8 weeks for their online therapy program they will be informed and invited by IPRP staff to participate in the MyP study. A MyP study flyer with contact phone number and e-mail address will be used.

at the UCSF Osher Center: Our institute is highly experienced in recruiting study participants. We will hang out flyers in its clinic space and may contact participants in prior studies who have consented to be contacted for further studies.

ResearchMatch.org will be utilized as a recruitment tool for this protocol. ResearchMatch.org is a national electronic, web-based recruitment tool that was created through the Clinical & Translational Science Awards Consortium in 2009 and is maintained at Vanderbilt University as an IRB-approved data repository. UCSF is part of the ResearchMatch network, so UCSF researchers are allowed to use this registry with IRB approval.

How it works: Anyone residing in the United States can self-register as a potential research volunteer on ResearchMatch.com. Once registered, volunteers’ coded information becomes part of a pool of data that researchers can search through when looking for people to contact about their studies. The researchers then send a recruitment message (attached to this submission) to potentially eligible volunteers through ResearchMatch's secure web system. After receiving the recruitment email from ResearchMatch, the volunteer can click a button to release their contact information to the researcher if they want to learn more about the study. All volunteer information is kept confidential until the volunteer decides to release it.

Determination of Eligibility:

After making initial contact with the subject and prior to enrollment, eligibility screening will be conducted by trained research staff. Due to the extensive nature of the eligibility screening, participants will be asked to give verbal consent (see section 10.7) before the screen is conducted. If a potential subject is interested in participating and provides verbal consent, he or she will be asked a series of eligibility questions. If he/she meets initial requirements for preliminary eligibility and is interested in participating, the potential participant will be invited and potentially scheduled for a zoom-based appointment with the study team at the UCSF Osher Center.

Eligibility will be determined after 2 steps: (1) Potential participants, which have heard or read about the study, will be screened by phone by the SRA. If passing eligibility criteria and after providing informed consent, (2) provisionally eligible potential participants will sign informed consent and then answer the MAIA online at home by Qualtrics. If their summary score is below the population mean score of 3.41 and the Non-Distraction score is below 2.91 [possible range 0-5], they are fully eligible and will be informed by phone and/or e-mail about their eligibility by the SRA. The value of 3.41 is the mean value of a sample of primary care patients at Kaiser Permanente in a prior study, and the value of 2.91 is the mean value plus standard deviation in the same sample.^10^ This eligibility criterion was chosen to test the hypothesis that the MIET task will be able to increase interoceptive awareness in patients with chronic low back pain with below average interoceptive awareness and preference for distracting themselves from their pain experience.

Potential participants enrolled in REACH have passed the same eligibility criteria applied to the Mind your Pain study except one: the below-average value of the MAIA summary and Non-Distraction scores. Once reached over the phone and after providing informed consent, these provisionally eligible potential participants will answer the MAIA online at home by Qualtrics or REDCap. They will be eligible with the same below-average scores defined above.

^10^: Mehling, W., Daubenmier, J., Price, C., Acree, M., Bartmess, E., Stewart, A. (2013). Self-reported interoceptive awareness in primary care patients with past or current low back pain. *J Pain Research* 2013:6 1–16. PMCID: PMC3677847.

Initiation of Contact/Recruitment:

Investigator's colleagues in clinics (i.e. pain clinic; Osher Center clinic, Family Medicine Clinic, etc) may introduce study to potential participants and provide them with the study team's recruitment material(s) and/or contact information.

As outlined above, potentially eligible participants at UCSF and at the SFVAMC will be handed flyers for this study.

We will use the UCSF study website, UCSF recruitment tools, and social media (such as facebook adds).

We will use a telephone screening script, a recruitment letter, or e-mail template approved by UCSF-IRB.

All recruitment is done by trained research staff who are assigned to this study, using aforementioned recruitment methods as well as methods listed below. Subjects can either contact the study staff, or study staff may contact the subject. The study team will not initiate contact with subjects by telephone unless they have received permission from the potential subject to be contacted by telephone. Permission is provided verbally (in the case of provider referrals), implied or written (in the case of VA approved research mailings), or explicitly by the potential subject. Initial contact by the study team must be in-person (including by phone and/or zoom) or by mail, unless the subject initiates contact. Upon contact, subject will be given information about the study. They will then be asked if they are interested in participating and will be screened for eligibility.

Interested candidates who either 1) respond to recruitment solicitation and initiate contact with the study team via e-mail or phone or 2) who are approached by the study team directly will be given an overview of the study and its enrollment requirements and will be initially screened by an online screener and telephone. If a potential participant is interested and meets initial requirements for participation, he/she will be invited for a zoom appointment with a trained staff member (CRC) at the UCSF Osher Center or the PI. This appointment, which will take place via zoom, will entail meeting with trained research personnel (CRC) who will describe the study in detail, address any questions/concerns, and obtain informed consent for study participation.

Recruitment strategies: Recruitment methods and media may include flyers; in-person presentations; a study specific webpage; informational letters; print newsletters; press releases or advertisements in print, internet, television, and radio, public service announcements; public notice-board postings; contact with and referral from relevant clinicians; social media, pamphlets; informational sessions about the research. In clinical settings, care providers will also be given informational materials to distribute to potential candidates.

The above-mentioned recruitment strategies will also take place at social service agencies, community mental health clinics, community organizations/events, including local professional organizations, consenting support and recovery centers, local hospitals and healthcare systems, regional employee assistance programs, religious organizations, cultural centers, public transportation vehicles and stations, social clubs, and universities.

Any recruitment materials (fliers, brochures, etc.) that will be used will be submitted to IRB for approval prior to use.

My Chart Recruitment:

MyChart (Apex) conducts a search for patients based on the study’s inclusion and exclusion criteria. This is a completely computer-aided search, meaning the computer—and not a person-- searches patient charts. When a patient is identified as potentially eligible, they receive an email from MyChart that says to log in to MyChart to read about a study they might be interested in. The email is short and is the same for every recipient—there is no patient-specific, study-specific or disease information in it.

When the patient logs into MyChart, there is a new “Research” tab with template information about participating in research and how to opt out of receiving recruitment messages. Then, the patient can click through to learn about a specific study they may be eligible for.

The patient has the option of clicking a link/button to let the study team know that they are interested in learning more about the study. Only if the patient takes this action will the study team receive information about the patient. If the patient clicks “No thanks” or simply does not respond, they will not be contacted by the study team, they won’t receive any follow-up emails from MyChart about this study, and their information will not be shared with the study team.

Consent Process:

| \| **Prior to eligibility determination (phone screening):**  In order to determine a subject's eligibility, a phone screening will be conducted. We will obtain verbal consent, as in-person consenting is not an option. Verbal consent is obtained by trained phone screeners. Verbal consent for phone screen is part of the phone screen itself.  **Prior to enrollment:**  After determining the subject is preliminarily eligible and prior to enrolling them in the study, the subject will receive consent documents by mail (with post-marked return envelope) or/and e-mail.  In a separate phone call or zoom meeting, trained research staff will obtain consent and go over the various sections of the consent documents, ensuring that the subject has comprehended the consent documents and does not have any questions. Participants will sign consent via docusign or mailed forms. \| \| --- \| |
| --- | --- |

Only trained study staff will be obtaining consent from participants. These study staff will have gone through training with either the PI or the study coordinator. Training includes but is not limited to becoming familiar with consenting guidelines, mock consent practice, etc.

The study team will engage the potential participant in a dialogue, using open-ended questions about the nature of the study or the experimental treatment, the risks and benefits of participating, and the voluntary nature of participation. Potential participants will be asked or shown a series of questions to assess their understanding of the study purpose, procedures, risks and benefits, as well as the voluntary nature of participation (especially appropriate when the consent process happens online or through a mobile health app).

No information will be collected prior to any form of consent. To determine eligibility over the phone, information on MRI contraindications, general mental health, and physical health will be obtained to assess whether someone can complete the study. All information obtained before informed consent can be seen in the Eligibility Phone Screen, attached in Other Study Documents. All of the health information collected is necessary to determine eligibility. Because this information is collected prior to an in-person consent for eligibility purposes, a verbal consent will be obtained.

All data collected for eligibility purposes is collected via VA RedCap or Qualtrics and will remain electronic throughout the course of the study. While this data will not be used for analysis, it will be preserved as it is used to contact potential future participants (upon their consent to do so). All retained data collected is behind the secure UCSF firewall.

Subject identifiers will be kept separately from the research data in locked cabinets and locked office at the UCSF Osher Center.

Risks and Benefits:

Data are password-protected and stored on secured UCSF PHI drive, only accessible on UCSF server. The computers will be checked regularly for proper and safe operation. Identifiable information is also stored as a hard copy in a locked office and locked file cabinet. Subjects will be carefully screened and subsequentially informed about the fact that the principal investigator will be available at all times during the experiment. Subjects are informed that they may end the test session at any time and that participation in this research is voluntary.

Subjects are told that all information obtained is completely confidential and that their medical treatment at UCSF or the VA will not be affected, whether or not they choose to participate in this study. Coded numbers are assigned to each file in the data base to ensure patient privacy. To minimize the risk that sensitive subject information may be disclosed, all subject information will be kept in locked cabinets or in databases with secured passwords.

Records and data will be rigorously protected. Examiners will be clinically trained and sensitive to signs of stress, anxiety, or fatigue so that testing will be immediately terminated should any subject experience signs of discomfort. Any incidental findings regarding subjects' health will be submitted to his/her physician at subjects' request.

**Risk management procedures associated with sensory function testing:**The subjects will be familiarized with the sensory testing procedures prior to testing. Subjects will receive various levels of painful and nonpainful stimuli and will be asked whether they would like to participate in this study. This will give the subject the opportunity to experience the level of painful stimuli that will be applied and to decide whether he/she wants to continue participating in the study. The sensory tests may cause some pain and/or discomfort and/or temporary reddening of the skin. However, with the proposed stimulus parameters these stimuli will not damage the skin or subcutaneous tissue. We maintain that this is a minimal risk, non-invasive procedure which is not known to produce adverse effects. Indeed, the 15 years that Dr. Strigo has been doing this procedure, there have been no adverse effects. Thus, we believe this is justification for classifying this as a minimal risk study. Subject are allowed to end participation if their perceived pain from thermal procedures is too great to tolerate.

**Reportable information:**Evidence of elder or child abuse, as well as threat to self or others, will be reported. Participants are notified of this in the verbal and in-person consents. If it is suspected that the subject is in danger of harming him/herself or someone else, or if child abuse or neglect or elder abuse has occurred, appropriate authorities will be notified as required by law.

**Self-report measures:** The main risks associated with these procedures are fatigue and irritability with the testing procedure. The investigator and research assistants are trained to frequently check the subjects about their willingness and ability to continue with the testing. If subjects express concerns about continuing with the testing, the investigator has instructed the research assistants to stop testing, offer a break, or, in case the subject is not willing to continue, to terminate the testing session. Overall, however, previous comparable studies have not resulted in any significant discomfort or anxiety expressed by the participating subjects.

**Risk management procedures associated with sensory/repeated heat/thermal grill stimulation:** The subjects will be familiarized with the thermal stimulations prior to testing. Subjects will receive various levels of painful and non-painful stimuli and will be asked whether they would like to participate in the study. This will give the subject the opportunity to experience the level of painful stimuli that will be applied and to decide whether he/she wants to continue participating in the study. The thermal grill and heat stimulation equipment will be regularly checked by the investigators for accurate temperature application so that the programmed temperature corresponds to the applied temperature.

**Risks associated with functional magnetic resonance imaging:**According to the FDA, there is currently no evidence that MRI with approved scanners of up to 7 Tesla signal strength are associated with adverse effects. moreover, other fMRI centers across the country are regularly using up to 7T MRI scanners for research purposes. However, there are two major sources of risk. First, the subject may experience discomfort being in the confined and sometimes noisy environment of the scanner. Second, the strong magnetic field will affect electronic, magnetic, and metal devices that subjects carry with them or that have been implanted in the subject's body. Additionally, female subjects capable of childbearing, will be asked a number of questions regarding their use of reliable contraceptive methods in order to be as sure as possible that they are not pregnant. Even though there are no known risks to an unborn child associated with fMRI, women of child-bearing potential who are not using reliable contraceptive methods will be excluded from this study.

**Data collection:**The risks involve some degree of loss of privacy. This will be minimized as much as possible, as described elsewhere in this application.

Information regarding drug/alcohol will be obtained via the attached eligibility phone interview, and ,may be considered embarrassing or may be illegal/stigmatized. Subjects are reminded that they are free to decline any questions that they are not comfortable providing a response for.

Reports of elder or child abuse, or indications of harm to self or others, will be reported and may result in criminal or civil liability.

Only designated study personnel included explicitly on the current IRB application will have access to any study records (both paper and electronic). No research results will be added to the patient's medical record unless medically necessary. Data will only be shared with those parties specified on the HIPAA form, which includes UCSF, UCSF IRB personnel, VA regulatory personnel, and the subject's primary care physician in the event of a medical complication.

Additionally, U.S.C 7332-protected sensitive information will be protected by the same rigorous standard as all other data. identifying information associated with this data will never be shared in reports or at conference proceedings, as with all other data.

Only designated study personnel included explicitly on the current IRB application will have access to any study records (both paper and electronic). No research results will be added to the patient's medical record unless medically necessary. Data will only be shared with those parties specified on the HIPAA form, which includes UCSF, UCSF IRB personnel, VA regulatory personnel, and the subject's primary care physician in the event of a medical complication.

Additionally, U.S.C. 7332-protected sensitive information will be protected by the same rigorous standard as all other data. Identifying information associated with this data will never be shared in reports or at conference proceedings, as with all other data.

VA research records will be retained within the VA-protected environment (e.g., VA server – please include the R Drive folder location/name, VA lab location, etc.), only accessible by authorized VA personnel, and disposed in accordance with the VHA Records Control Schedule (RCS 10-1). Any VA data shared with an external entity will be transmitted via FIPS 140-2-compliant encrypted methods as required per VA policy.

Financial considerations:

Subjects will be compensated for their time. Subjects will receive $100 per visit to the SFVAMC (2 visits = $200). They will receive $15 for travel reimbursement for each individual visit. Therefore, if a subject completes these 2 visits, they will receive up to $230 for SFVAMC visit compensation and travel compensation.

For the qualitative interview at the UCSF Osher Center, 20 of the 30 participants will receive $100 each and $15 for travel reimbursement.

For responding to the EMA, participants will receive $0.50 for each completed EMA assessment, up to $2.50 per day over 8 weeks (56 days), or up to $140 total.

Participants will be compensated a total of up to $485 for their participation in the study (inc. travel reimbursement).
