# Supplementary material for: Mind your pain: A single-arm feasibility study to assess a smartphone-based interoceptive attention training for patients with chronic low back pain
Source: PLoS One. 2024 Oct 24;19(10):e0307690. doi: 10.1371/journal.pone.0307690 (PMC11500934; doi:10.1371/journal.pone.0307690)
Supplement: S2 File — (DOCX) [file pone.0307690.s002.docx]

**S2 File: Handout:**

**NEUROMATRIX OF PAIN**

**7 Facts from Research:**

**Fact #1)** **Acute pain** originates from the perception of nerve signals from receptors in our body’s tissues that detect potentially damaging processes (nociception). This signal **is protective**. It asks for attention and for action. The goal is to support recovery/tissue repair/healing and to protect your body by avoiding additional exposure to damaging movements or other stimuli. It is similar to the parking sensors on newer cars. Like with these sensors, the signal does not stop the movement of the car. Instead, it indicates that you are getting close to the next car: you need to pay attention, to act by pushing the break, and to stop in order to avoid any damage to your car. There is a loud signal, but not any damage if we act in time. However, ignoring the signal would cause damage to the body of the car. And if damage happens, the signal may not stop beeping. Our body is different from a car in that it has an amazing potential for healing itself. But similar to the car alarm, the signal may persist after the injury has healed. The computer may need a reset.

**Chronic pain** is very different from acute pain. It does not necessarily come from an actual threat to our body. It **is often overprotective** and is closely connected to our emotions (such as “I do not like this!” or “Nobody likes pain”). But the intensity of chronic pain is not closely associated with the degree of tissue damage (as shown with MRI). For a striking example, in phantom pain from a limb that has been lost, pain is felt in a limb that does not exist anymore. Chronic pain can be like an overactive parking sensor that keeps beeping even after damage to the car has been repaired. Or it beeps way too early, long before you really need to push the breaks and stop. The electronic alarm system is no longer a good indicator for what action is needed, for when to stop or when we can safely go. Similar with chronic pain: the brain may think “danger” - and alarm us - when there is no danger.

**Fact #2)** **All pain is real**. Pain is not in our imagination, it is not fantasized or simply made up. It is an experience with specific felt qualities. One could say that it is composed of these qualities or characteristics: e.g. a feeling of dullness or sharpness; tightness or looseness; feeling more stagnant or moving/changing; more cold or hot; more clearly defined with a three-dimensional shape or more diffuse. Pain is a subjective reality that can be seen in brain area activations.

**Fact #3) Hurt is not harm** in chronic pain: hurt is what we feel, the result of brain processes. Hurt calls for our attention and is supposed to be an indicator for what to do to protect our bodies from damage, but with chronic pain the intensity of the hurt is not a reliable gauge or indicator for assessing any potential harm. Most importantly, **pain does not mean my body is damaged**. Deep tissue massage, trigger point massage, relaxing over a foam roller or tennis ball can cause pain, but no damage. Some people even call it “good pain”.

**
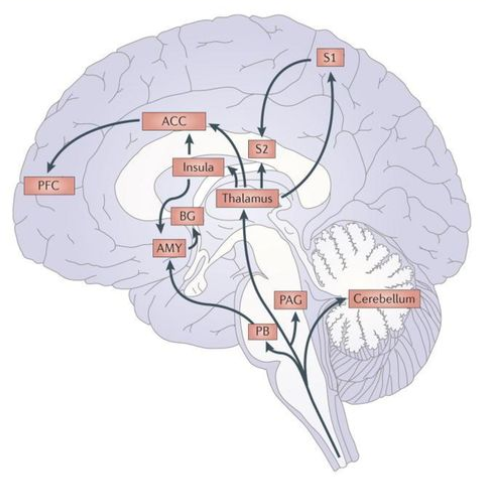
Fact #4)** ***All* pain experience requires the involvement of the brain** when it perceives that danger to body tissue could exist and that action may be required. The brain is a truly amazing, fantastic organ with a complex network of connections between multiple brain regions that activate with pain: the spinal cord, the thalamus, the periaqueductal gray (PAG), the insula, the anterior cingulate cortex (ACC), amygdala, hippocampus and the frontal cortex (no need to remember these names). The sensation of pain may fill a specific space in our body, e.g., the low back, **where** we feel the pain, but the signal from that site in our back that is going from the bottom of the spinal cord up to the brain may just be a call for attention-- or a memory of such a call - that gets transformed in the brain into what we experience as pain. This transformation is the end-product of the interaction of many involved brain regions, where memories are stored and compared to the current felt experience, where expectations are stored and built, emotional reactions are formed, thoughts are made, and beliefs are held (🡪 figure to the right). The intensity of the pain depends on this brain processing, which is called the “**neuromatrix of pain**”. But if the central computer has a glitch, e.g. does not correctly assess how close I am to the next car and goes off too early, or keeps beeping after injury repair, it becomes unreliable, irritating, and bothersome. If this glitch in the computer persists, the brain connections change, some become more and some less active. The more a nerve track is used, the better it becomes in its signaling ability,
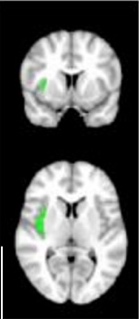

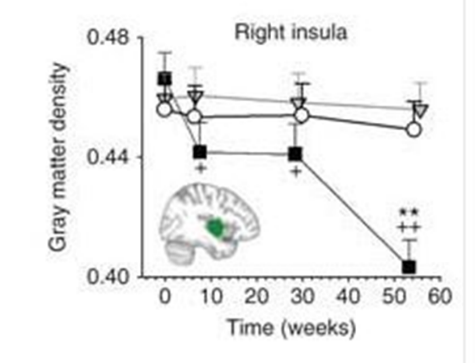
which is called **neuroplasticity**. On the other hand, if it is not used, it atrophies, shrinks. We now know specific brain regions that shrink when pain turns chronic, and that these can recover with successful treatment (research with images published in peer-
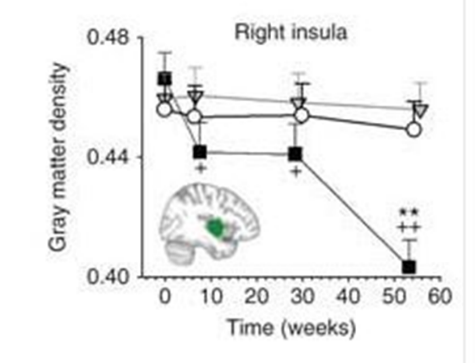
reviewed science journals by: Baliki: *Nature* *Neuroscience* 2012; Hashmi: *Brain* 2013).

*Left*: Slices of the brain with insula cortex in green. *Right*: Nerve cell density in insula cortex stays the same for healthy individuals ( ) and for those in which acute low back pain resolves ( ). But it diminishes in those for which pain becomes chronic ( ) over 1 year.

The good news is that **neuroplasticity goes both ways**. We can change the brain, reactivate weakened brain connections, and reprogram the overprotective signaling. This takes time and may not completely stop the alarm, but it can change the signal threshold and dial the signal intensity down. Shrunken brain areas can recover. **We can retrain the fired-up alarm system.**


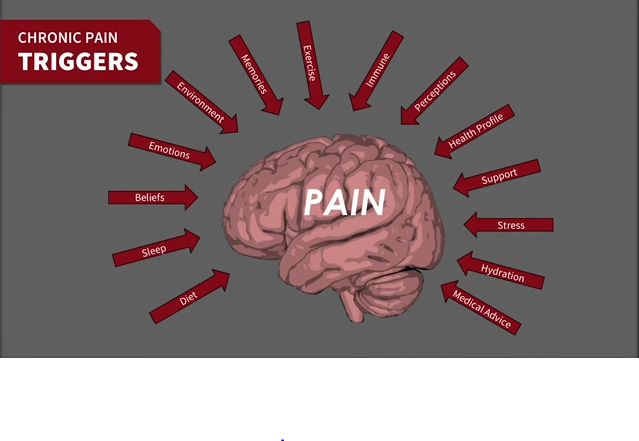


**Fact #5)** The **neuromatrix of pain** is a complex and amazing network of connections in the brain, where **many triggers** (image: Stanford.edu website) come in that **modulate the pain intensity**, dialing it up or down. The sensation that we call chronic pain is constructed in the brain from many different influences. It is not just a simple indicator for a potential harm to the body’s tissues.

**
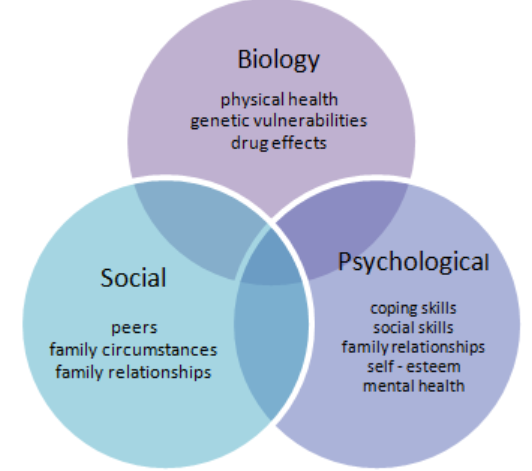
**This scientific insight is also called the biopsychosocial model of pain. Signals from the body’s tissues (discs, joints, ligaments, wherever we have nerve endings in our body) get computed together with memories (“I had that sensation before”), beliefs (“I think something must have gone very wrong!” or “I think I can ignore that”), emotions (e.g. anxiety, frustration, anger, depression), expectations (“oh my god, this may ruin my career, hobby, vacation!” and/or “I better not move at all.”), habits (incessantly checking for phone messages), worry and stress from daily life. Here is a short video by neuroscience pain expert Lorimer Moseley that shows how pain can be a false alarm created in the brain. <https://www.youtube.com/watch?v=Sjes9CuN6Ko>

It can be quite challenging to convince ourselves that the sensation we call chronic back pain is overprotective, a false alarm regardless of MRI findings. 40-60% of people walking down the street without pain have the same MRI findings as people with pain. It may help to consider a few personal points that indicate that our pain may not be well-explained by the MRI findings: did my pain originate during a period of stress? Did it originate without a physical injury? Does my pain vary a lot? Is it gone in times of happiness and joy? Does its location change? Does it get worse with bad news or in a stressful situation? Am I worried a lot? (more details in: Alan Gordon: The Way Out; Avery Penguin Random House, 2021)

**
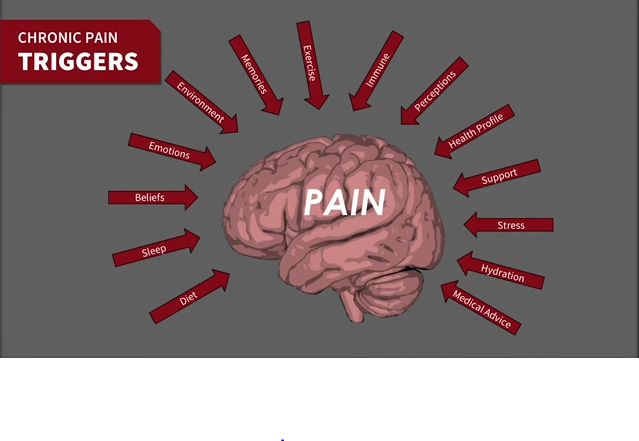
Fact #6)** **Pain needs attention**: From research, we know the brain regions that shrink when pain persists and becomes chronic pain (see above in **4**). This appears to occur primarily in two regions. One is the hub for the *reward system*, where the brain processes joy, contentment, happiness, excitement, from savoring chocolate to sex and drugs, (it is called the nucleus accumbens, but remembering names is not important.) The other is the central hub for *interoception and the salience network for detecting and filtering* salient stimuli, discriminating which *body sensations perceived from inside the body* are worth our attention (it is called the insula, the Latin word for island, but you do not have to remember any names.) Pain is a sensation that is *felt* inside our body. The atrophy of the insula is most likely related to our habit of trying to ignore our pain. Who enjoys pain? Nobody wants to really feel it. Distraction does work well for coping with *acute* pain, but research studies have shown that it does not help with *chronic* pain. We believe that **savoring positive emotions**, sensing and enjoying the “good” feelings that come from our body where it does not hurt (there are parts of our body that still can feel good), **and** **paying close and mindful attention to the details of the sensation that we call pain** (sometimes called Somatic Tracking), may restore diminished brain regions, retrain and reprogram the signal processor, and dial down the signal. We define “close and mindful attention” as noticing how the pain sensation actually feels. What are the characteristics of the pain right now? It is about SENSING the pain, not about evaluating it, or your thoughts about the pain or the stories that come with it, or your history and expectations for the future. In science this is called “interoceptive awareness”, the awareness of what we perceive consciously from inside the body – which includes the sensation we call pain. Recently in 2020, this was declared a research topic of “high priority” by the National Institutes of Health (NIH). It is a new way of thinking about recovery from chronic pain. It is worth remembering that retraining our brain takes time and perseverance. Neuroplasticity happens slowly, little by little. You can do it.

**Fact #7)** **Pacing helps to move smarter, not harder**. *Avoiding* movement out of fear of harming our body is not the way to recovery from chronic pain. On the other side, we do not want to trigger our body’s warning system either by “wrong” movements that irritate the structures of the spine (including the soft tissues around the vertebra) or by simply doing too much. It is not smart to ignore *any* warning signals. If we want our body and brain to learn new ways of dealing with chronic pain, we need to follow the Goldilocks’ rule and find a right middle way to safely *trigger healing responses* rather than triggering an overprotective alarm system. That requires that we take time to explore, to find out which movements are safe and do not hurt, and when exactly it starts to hurt without causing any harm, when some hurt is actually still safe. We can do that by carefully and mindfully exploring movements we are exposed to in daily life, such as walking, or with the yoga movements and postures of our program. We also need to dose the intensity and duration of our activities to an amount that is safe and just right for triggering the body’s healing activities without triggering overprotective reactions.

<https://www.londonpainclinic.com/resources/pacing-and-chronic-pain/>

**SUMMARY:**

1) Chronic pain is different (often overprotective) from acute pain (protective).

2) All pain is real.

3) In chronic pain, hurt is not harm. It may be a false alarm.

4) The experience of pain involves our brain, learning, and two-way neuroplasticity.

5) Many factors are involved in the neuromatrix of pain, not only the condition of the body’s tissues as shown in the MRI.

6) Savoring positive emotions *and* paying close sensory attention are needed to *retrain the brain.*

7) Mindful attention to carefully regulate pacing is needed for triggering a healing response.
