# Supplementary material for: Mind your pain: A single-arm feasibility study to assess a smartphone-based interoceptive attention training for patients with chronic low back pain
Source: PLoS One. 2024 Oct 24;19(10):e0307690. doi: 10.1371/journal.pone.0307690 (PMC11500934; doi:10.1371/journal.pone.0307690)
Supplement: S3 File — (DOCX) [file pone.0307690.s003.docx]

**S3 File: Measures**

**BACBAC Biobehavioral Questionnaires 3/5/2020**

**In accordance with the BACPAC study, MyP used the following questionnaires.**

| *Acronym* | | *Biopsychosocial Questionnaires* | | *# of items* | *min* | *Author(s)* |
| --- | --- | --- | --- | --- | --- | --- |
|  |  | |  |  |  |  |
| Minimum Dataset | | PROMIS-29+2 Vs 2.1  PHQ-2  GAD-2  2 extra items from PROMIS Sleep Disturbance 6a (not in PROMIS-29)  Sleep Duration (in file: BACPAC MDS)  Pain Catastrophizing Scale-SF 6. ((in file: BACPAC MDS)  TAPS  (+ 12 outcome measures) total | | 31  2  2  2  1  6  4  48+12 | 10 | PROMIS[^1^](#_ENREF_1)  Kroenke et al, 2003[^2^](#_ENREF_2)  Kroenke et al, 2007[^3^](#_ENREF_3)  Yu et al, 2011[^4^](#_ENREF_4)  Kurina et al, 2013[^5^](#_ENREF_5)  McWilliams, 2015[^6^](#_ENREF_6) |
|  |  | |  |  |  |  |
| PD-Q | | Pain DETECT Questionnaire (only 7 central items) | | 7 |  | Freynhagen et al., 2006[^7^](#_ENREF_7) |
| FABQ-PA | | Fear-Avoidance Beliefs Questionnaire – Physical Activity | | 5 | 20 | Waddell et al., 1993[^8^](#_ENREF_8) |
| CPAQ-8 | | Chronic Pain Acceptance Questionnaire-SF8 | | 8 |  | Fish et al. 2010[^9^](#_ENREF_9) |
| CSQ-24 | | Coping Strategies Questionnaire CSQ-24 (minus 6 for Catastrophizing) | | 18 |  | Harland, Georgieff, 2003[^10^](#_ENREF_10) |
| MAIA-2 | | Multidimensional Assessment of Interoceptive Awareness (4 of 8 scales) | | 22 |  | Mehling et al., 2018[^11^](#_ENREF_11) |
| BPCQ | | Beliefs in Pain Control Questionnaire | | 13 |  | Brown, 2004[^12^](#_ENREF_12)^,^[^13^](#_ENREF_13) |
| PSS-4 | | Perceived Stress Scale | | 4 |  | Cohen et al., 1983[^14^](#_ENREF_14) |
| iPANAS | | International Positive and Negative Affect Schedule 10-item SF | | 10 |  | Watson et al., 1988[^15^](#_ENREF_15)^,^[^16^](#_ENREF_16) |
| PROMIS ES | | PROMIS Emotional Support 4a V2 | | 4 |  | Tucker et al, 2014[^17^](#_ENREF_17) |
| PSEQ-4 | | Pain Self-Efficacy Questionnaire 4-item version (items 4, 6, 8, 9) | | 4 |  | Chiarotto et al, 2016[^18^](#_ENREF_18) |
| PCPSS | | Adverse Childhood Life Event / Primary Care PTSD Symptom Screener | | 1🡪+5 |  | Prins et al, 2016[^19^](#_ENREF_19) |
| FS | | Financial Strain | | 1 |  | Puterman et al, 2012[^20^](#_ENREF_20) |
| PD | | Perceived Discrimination | | 3 |  | Braveman et al, 2019[^21^](#_ENREF_21) |
| EPR | | Expectation of Pain Relief | | 1 |  | Cormier et al, 2016[^22^](#_ENREF_22) |
| PASS-20 | | Pain Anxiety Symptoms Scale - Short Form: (items 6-10, 16-20) | | 10 |  | McCracken et al. 2002[^23^](#_ENREF_23) |
| PO-SF | | Positive Outlook Short Form | | 6 |  | Greco et al, 2016[^24^](#_ENREF_24) |
| FFMQ | | Five Facets of Mindfulness Questionnaire | | 39 |  | Baer et al., 2006[^25^](#_ENREF_25) |

**PROMIS-29** is a standard measure for health-related quality of life and has been extensively validated.[^1^](#_ENREF_1) PROMIS-29+2 includes 2 items for cognition. (<http://www.healthmeasures.net/index.php?option=com_instruments&view=measure&id=1050&Itemid=992>).

**PainDETECT Questionnaire (PD-Q)**[^7^](#_ENREF_7): *Rationale*: The PD-Q was developed and validated in co-operation with the German Research Network on Neuropathic Pain to detect neuropathic pain. These items can be used instead of the 7 items added in the SF-MPQ-2 version of the McGill. *Method:* 7 items for neuropathic pain quality descriptors on a 6-point scale, scored by multiplying the answer scores by the numbers of items scored that way. It has shown reliability and validity in patients with cLBP and numerous other conditions and is used worldwide.[^26^](#_ENREF_26)

**Pain Catastrophizing Scale SF (PCS-6)**[**^6^**](#_ENREF_6): *Rationale:* Changes in catastrophizing have been reported as mediating therapeutic improvements for cLBP. The PCS is widely used internationally for this key parameter. *Methods*: The PCS has 13 items for 3 subscales with *α* for the total PCS .92., and 85, .75, and .86, respectively, for Rumination, Magnification, and Helplessness.[^27^](#_ENREF_27) Items 4, 5, 6, 10, 11, 13 create the short form for the 3 subscales, which correlates at .95 with the original scale.[^6^](#_ENREF_6) Alpha is .88.[^6^](#_ENREF_6)

**Fear-Avoidance Beliefs Questionnaire Physical Activity (FABQ-PA)**[^8^](#_ENREF_8): *Rationale*: Kinesiophobia is defined by the developers as “an irrational, and debilitating fear of physical movement and activity resulting from a feeling of vulnerability to painful injury or re-injury”. (Kori et al., 1990) Fear of movement or of re-injury has been found to one of the main predictors of persistence of pain. *Method*: The 5-item Physical Activity (PA) scale is much shorter than the TAMPA scale. (A second Work Activity (WA) scale is only for working population.) It contains 5 items on a 7-point Likert scale inquiring about beliefs regarding pain and is widely used internationally. It reflects the belief that activity may result in (re)injury or increased pain. Reliability, validity and sensitivity to change are well supported.[^28^](#_ENREF_28)

**Chronic Pain Acceptance Questionnaire SF8 (CPAQ-8)**[**^9^**](#_ENREF_9): *Rationale:*  Acceptance of chronic pain has been shown to be associated with the trajectory of chronic pain; it is modifiable by MBSR.[^29^](#_ENREF_29) It is a precondition for the capacity to develop mindful interoceptive awareness of pain. *Methods:* The CPAQ-8 has 8 items and two factors: Activity Engagement and Pain Willingness. It has shown strong correlations (.93 and .95) with the 20-item original version, which is widely used internationally. CPAQ-8 demonstrated good psychometric properties and sensitivity to rehabilitation changes.[^30^](#_ENREF_30)

**Coping Strategies Questionnaire (CSQ-24)**[**^10^**](#_ENREF_10): *Rationale*: According to the psychological stress appraisal model coping styles have a major influence on distress and pain. The CSQ-24 is an abbreviated version of the original 50-item CSQ[^31^](#_ENREF_31), shortened to 32-items,[^32^](#_ENREF_32) and validated with 24 items (losing e.g. the Ignoring Pain subscale) by Harland and Georgieff (2003) to assess pain coping strategies used by pain patients. *Methods*: It is composed of 24 items (one global coping item and 23 specific coping items) and one global item. The internal consistency reliability coefficients (Cronbach’s alpha) of .85, .84, .77, and .75 are moderately high.[^10^](#_ENREF_10) It contains 6 items for Catastrophizing already included in the PCS. The remaining subscales are for Diversion, Reinterpreting and Cognitive Coping using a 7-point Likert scale.[^10^](#_ENREF_10)

**Multi-dimensional Assessment of Interoceptive Awareness – Version 2 (MAIA-2)**[**^11^**](#_ENREF_11): *Rationale:* The MAIA has become a standard self-report measure for interoceptive awareness or sensibility, purportedly a key mechanism of action for mind-body interventions[^33^](#_ENREF_33), and 4 of its 8 scales have shown to be a strong predictor of treatment response for cLBP.[^34^](#_ENREF_34) *Methods*: we will use 22 items for 4 scales Not-Distracting, Attention Regulation, Emotional Awareness, and Self-Regulation, answered on a 6-point Likert scale with acceptable Cronbach *α* values, excellent sensitivity to change and validity.[^11^](#_ENREF_11)

**Beliefs in Pain Control Questionnaire (BPCQ)**[**^13^**](#_ENREF_13)**:** *Rationale:* The BPCQ assesses beliefs about controlling pain and has 3 subscales: The Internal Scale measures beliefs that pain is personally controlled, the Powerful Doctor scale that pain control is in the hands of powerful others, and the Chance Events scale that it is controlled by chance or misfortune. *Method*: 5 items for each subscale are responded to on a 6-point Likert scale.[^12^](#_ENREF_12) Cronbach’s Alpha for the first two scales was excellent, for Chance Events was inadequate.[^35^](#_ENREF_35)

**Perceived Stress Scale – 4 (PSS-4)**[**^14^**](#_ENREF_14): *Rationale*: Perceived stress has shown to be associated pain perception. The PSS is applied worldwide with numerous clinical conditions that vary according to stress levels. *Methods*: Four items ask about feelings and thoughts in the past month on a 5-point Likert scale. It is the shortest version of the PSS that has shown excellent validity and reliability in multiple versions.

**Adverse Childhood Life Events/ PTSD:** *Rationale:* ACLE and PTSD are predictors of chronic pain. *Method:* we will use a single clinic use screening tool for ACLE and PTSD: **a single Primary Care PTSD screening tool**[**^19^**](#_ENREF_19), that if answered positively, branches out to 5 sub-items: “Sometimes things happen to people that are unusually or especially frightening, horrible, or traumatic. For example: a serious accident or fire, a physical or sexual assault or abuse, an earthquake or flood, a war, seeing someone be killed or seriously injured, having a loved one die through homicide or suicide. Have you ever experienced this kind of event? No/Yes A) as a child? B) as an adult?” If Yes 4 PTSD symptom items are asked. The addition asking for “as child or adult” is a modification.

**Positive and Negative Affect Schedule (PANAS)**[**^15^**](#_ENREF_15) **SF**: *Rationale*: Emotion are strongly correlated to pain perception as the brain regions for emotions and pain overlap. Chronic pain has been proposed to be viewed as a homeostatic emotion.[^36^](#_ENREF_36) *Methods*: 5 items for frequency of each positive or negative emotions are assessed on a 5-point Likert scale. The PANAS is a reliable and valid measure of the constructs it was intended to assess, although the hypothesis of complete independence between positive and negative affect was rejected.[^37^](#_ENREF_37) iPANAS SF is an internationally validated version.[^16^](#_ENREF_16)

**PROMIS Emotional Support 4a V2**[**^17^**](#_ENREF_17)**:** *Rationale*: Social support can predict life stress and in turn persistence of pain*. Method*: We will use the PROMIS measure for emotional support, which has been mapped against the World Health Organization's International Classification of Functioning.[^17^](#_ENREF_17)

**Pain Self-Efficacy Questionnaire (PSEQ-4)**[**^18^**](#_ENREF_18)**:** *Rationale*: Pain self-efficacy is an independent predictor of poor recovery after initial consultation for LBP and is described as the attitudes and beliefs that people with chronic pain hold to carry out certain daily activities, even in the presence of pain. *Methods:* We will use the 4-item PSEQ-4 as short versions of the 10-item PSEQ: items 4, 6, 8, and 9. Both versions are adequately responsive instruments in patients with CLBP.[^18^](#_ENREF_18)

**Social determinants of health:** *Rationale*: financial strain and perceived discrimination may predict chronic pain*. Method*: We will use single items for **Financial Strain** (used/developed in[^20^](#_ENREF_20))**:** “How hard is it for you (and your family) to pay for the very basics like food, medical care, and heating?” Response options: 1 = very hard, 2 = hard, 3 = somewhat hard, 4 = not very hard, and 5 = do not know; recoded into a binary variable, in which very hard and hard were considered high financial strain, and somewhat hard and not very hard were considered low financial strain.

**Perceived Discrimination:** *Rationale:* Perceived discrimination may be a stronger predictors than physical health variables in minorities.[^38^](#_ENREF_38) Methods: We will use three modified items used in a large statewide survey by the California Department of Public Health in its recent 2019 Maternal and Infants Health Assessment (MIHA). The items developed at UCSF assess perceived experience of discrimination and worries.[^21^](#_ENREF_21)^,^[^39^](#_ENREF_39) The modification is that we added discrimination for sexual orientation. The items are 1) Overall during your life until now, how often *have you worried* that you might be treated or judged unfairly because of your race, ethnic group or social discrimination? 2) Overall during your life until now, how often *have you worried* that a loved one like your spouse, partner, child, or parent might be treated or judged unfairly because of their race, ethnic group or sexual orientation?  3) Overall during your life until now, how often *have you been* discriminated against, prevented from doing something, or hassled or made to feel inferior because of your race, ethnicity, color or sexual orientation? Ordinal scale 0 = ‘never’; 1 = ‘not very often’; 2 = ‘somewhat often’; 3 = ‘very often’.

**Expectations of Pain Relief:** *Rationale:* expectations are strong predictors of treatment outcomes.[^40^](#_ENREF_40) Expectations of pain relief have shown stronger predictive values than expectations of pain intensity. *Methods:* we will use a single item that has been used in a large (*N* > 2,000) multicenter cohort study by the Canadian Institutes of Health Research with chronic pain patients (mostly cLBP) and was found to be the strongest expectation item predicting treatment outcome and patient satisfaction.[^22^](#_ENREF_22) “Please indicate how much pain relief you expect over the coming three months?” on an 11-point ordinal scale ranging from “no relief” to “complete relief” with 10% steps.

**Positive Outlook**: *Rationale*: Positive Outlook or Optimism are personality traits that have shown to predict treatment outcomes in pain studies with Ajay Wasan, UPitt. *Methods:* This is a 6-item scale developed by Carol Greco, UPitt in the Healing Encounters and Attitudes List according to PROMIS methodology.[^24^](#_ENREF_24) Its value in 400+ pain patients awaits publication.

**Pain Anxiety Symptoms Scale - Short Form (PASS-20)**[**^23^**](#_ENREF_23)**:** *Rationale:* There is a significant relation between fear and avoidance of pain and the suffering and disability of chronic pain. The concept of pain avoidance is highlighted. *Methods:* we will use items 6-10 for Avoidance and 16-20 for Physiological Pain Anxiety; the other subscales are already covered with anxiety and catastrophizing.

**Five-Facet Mindfulness Questionnaire (FFMQ)**[**^41^**](#_ENREF_41): *Rationale:* The FFMQ measures dispositional and, despite reported shortcomings,[^42^](#_ENREF_42) is among the most widely used mindfulness self-report measure. *Methods*: The FFMQ has 39 items for 5 scales with *α* between .77 and .92.[^42^](#_ENREF_42) The factor structure has been confirmed in non-meditators[^42^](#_ENREF_42).

References:

1. Craig BM, Reeve BB, Brown PM, et al. US valuation of health outcomes measured using the PROMIS-29. Research Support, N.I.H., Extramural. *Value Health*. Dec 2014;17(8):846-53. doi:10.1016/j.jval.2014.09.005

2. Kroenke K, Spitzer RL, Williams JB. The Patient Health Questionnaire-2: validity of a two-item depression screener. *Med Care*. Nov 2003;41(11):1284-92. doi:10.1097/01.MLR.0000093487.78664.3C

3. Kroenke K, Spitzer RL, Williams JB, Monahan PO, Lowe B. Anxiety disorders in primary care: prevalence, impairment, comorbidity, and detection. *Ann Intern Med*. Mar 6 2007;146(5):317-25. doi:10.7326/0003-4819-146-5-200703060-00004

4. Yu DT, Jones AY, Pang MY. Development and validation of the Chinese version of the Massachusetts General Hospital Acupuncture Sensation Scale: an exploratory and methodological study. *Acupunct Med*. May 22 2012;doi:10.1136/acupmed-2012-010145

5. Kurina LM, McClintock MK, Chen JH, Waite LJ, Thisted RA, Lauderdale DS. Sleep duration and all-cause mortality: a critical review of measurement and associations. *Ann Epidemiol*. Jun 2013;23(6):361-70. doi:10.1016/j.annepidem.2013.03.015

6. McWilliams LA, Kowal J, Wilson KG. Development and evaluation of short forms of the Pain Catastrophizing Scale and the Pain Self-efficacy Questionnaire. *Eur J Pain*. Oct 2015;19(9):1342-9. doi:10.1002/ejp.665

7. Freynhagen R, Baron R, Gockel U, Tolle TR. painDETECT: a new screening questionnaire to identify neuropathic components in patients with back pain. *Curr Med Res Opin*. Oct 2006;22(10):1911-20. doi:10.1185/030079906X132488

8. Waddell G, Newton M, Henderson I, Somerville D, Main CJ. A Fear-Avoidance Beliefs Questionnaire (FABQ) and the role of fear-avoidance beliefs in chronic low back pain and disability. *Pain*. Feb 1993;52(2):157-68.

9. Fish RA, McGuire B, Hogan M, Morrison TG, Stewart I. Validation of the chronic pain acceptance questionnaire (CPAQ) in an Internet sample and development and preliminary validation of the CPAQ-8. *Pain*. Jun 2010;149(3):435-43. doi:10.1016/j.pain.2009.12.016

10. Harland NJ, Georgieff K. Development of the Coping Strategies Questionnaire 24, a clinically utilitarian version of the Coping Strategies Questionnaire. *Rehabilitation Psychology*. 2003;48(4 . ):296–300.

11. Mehling WE, Acree M, Stewart A, Silas J, Jones A. The Multidimensional Assessment of Interoceptive Awareness, Version 2 (MAIA-2). *PLoS One*. 2018;13(12):e0208034. doi:10.1371/journal.pone.0208034

12. Brown CA. The beliefs of people with chronic pain in relation to 'important' treatment components. *Eur J Pain*. Aug 2004;8(4):325-33. doi:10.1016/j.ejpain.2003.10.005

13. Skevington SM. A standardised scale to measure beliefs about controlling pain (B.P.C.Q.): A preliminary study. *Psychology and Health*. 1990;4(3):221-233.

14. Cohen S, Kamarck T, Mermelstein R. A global measure of perceived stress. Research Support, U.S. Gov't, Non-P.H.S.

Research Support, U.S. Gov't, P.H.S. *J Health Soc Behav*. Dec 1983;24(4):385-96.

15. Watson D, Clark LA, Tellegen A. Development and validation of brief measures of positive and negative affect: the PANAS scales. *J Pers Soc Psychol*. Jun 1988;54(6):1063-70.

16. Thompson ER. DEVELOPMENT AND VALIDATION OF AN INTERNATIONALLY RELIABLE SHORT-FORM OF THE POSITIVE AND NEGATIVE AFFECT SCHEDULE (PANAS). *JOURNAL OF CROSS-CULTURAL PSYCHOLOGY*. 2007;38(2):227-42. doi:10.1177/0022022106297301

17. Tucker CA, Escorpizo R, Cieza A, et al. Mapping the content of the Patient-Reported Outcomes Measurement Information System (PROMIS(R)) using the International Classification of Functioning, Health and Disability. *Qual Life Res*. Nov 2014;23(9):2431-8. doi:10.1007/s11136-014-0691-y

18. Chiarotto A, Vanti C, Cedraschi C, et al. Responsiveness and Minimal Important Change of the Pain Self-Efficacy Questionnaire and Short Forms in Patients With Chronic Low Back Pain. *J Pain*. Jun 2016;17(6):707-18. doi:10.1016/j.jpain.2016.02.012

19. Prins A, Bovin MJ, Smolenski DJ, et al. The Primary Care PTSD Screen for DSM-5 (PC-PTSD-5): Development and Evaluation Within a Veteran Primary Care Sample. *J Gen Intern Med*. Oct 2016;31(10):1206-11. doi:10.1007/s11606-016-3703-5

20. Puterman E, Adler N, Matthews KA, Epel E. Financial strain and impaired fasting glucose: the moderating role of physical activity in the Coronary Artery Risk Development in Young Adults study. *Psychosom Med*. Feb-Mar 2012;74(2):187-92. doi:10.1097/PSY.0b013e3182448d74

21. Braveman P, Heck K, Egerter S, et al. Worry about racial discrimination: A missing piece of the puzzle of Black-White disparities in preterm birth? *PLoS One*. 2017;12(10):e0186151. doi:10.1371/journal.pone.0186151

22. Cormier S, Lavigne GL, Choiniere M, Rainville P. Expectations predict chronic pain treatment outcomes. *Pain*. Feb 2016;157(2):329-38. doi:10.1097/j.pain.0000000000000379

23. McCracken LM, Dhingra L. A short version of the Pain Anxiety Symptoms Scale (PASS-20): preliminary development and validity. *Pain Res Manag*. Spring 2002;7(1):45-50. doi:10.1155/2002/517163

24. Greco CM, Yu L, Johnston KL, et al. Measuring nonspecific factors in treatment: item banks that assess the healthcare experience and attitudes from the patient's perspective. *Qual Life Res*. Jul 2016;25(7):1625-34. doi:10.1007/s11136-015-1178-1

25. Baer RA, Smith GT, Hopkins J, Krietemeyer J, Toney L. Using self-report assessment methods to explore facets of mindfulness. Validation Studies. *Assessment*. Mar 2006;13(1):27-45. doi:10.1177/1073191105283504

26. Cappelleri JC, Koduru V, Bienen EJ, Sadosky A. A cross-sectional study examining the psychometric properties of the painDETECT measure in neuropathic pain. *J Pain Res*. 2015;8:159-67. doi:10.2147/JPR.S80046

27. Sullivan MJL, Bishop SR, Pivik J. The Pain Catastrophizing Scale: Development and validation. *Psychological Assessment*. 1995;7(4):524-32.

28. George SZ, Calley D, Valencia C, Beneciuk JM. Clinical Investigation of Pain-related Fear and Pain Catastrophizing for Patients With Low Back Pain. *Clin J Pain*. Feb 2011;27(2):108-15. doi:10.1097/AJP.0b013e3181f21414

29. la Cour P, Petersen M. Effects of mindfulness meditation on chronic pain: a randomized controlled trial. *Pain Med*. Apr 2015;16(4):641-52. doi:10.1111/pme.12605

30. Rovner GS, Arestedt K, Gerdle B, Borsbo B, McCracken LM. Psychometric properties of the 8-item Chronic Pain Acceptance Questionnaire (CPAQ-8) in a Swedish chronic pain cohort. *J Rehabil Med*. Jan 2014;46(1):73-80. doi:10.2340/16501977-1227

31. Rosenstiel AK, Keefe FJ. The use of coping strategies in chronic low back pain patients: relationship to patient characteristics and current adjustment. *Pain*. Sep 1983;17(1):33-44.

32. Swartzman LC, Gwadry FG, Shapiro AP, Teasell RW. The factor structure of the Coping Strategies Questionnaire. Research Support, Non-U.S. Gov't. *Pain*. Jun 1994;57(3):311-6.

33. Mehling WE. Differentiating attention styles and regulatory aspects of self-reported interoceptive sensibility. Review. *Philos Trans R Soc Lond B Biol Sci*. Nov 19 2016;371(1708)doi:10.1098/rstb.2016.0013

34. Vachon-Presseau E, Berger SE, Abdullah TB, Griffith JW, Schnitzer TJ, Apkarian AV. Identification of traits and functional connectivity-based neuropsychotypes of chronic pain. *bioRxiv preprint doi* [*http://dxdoiorg/101101/421438*](http://dxdoiorg/101101/421438). 2018;

35. Czerw A, Religioni U, Deptala A, Fronczak A. Application of the BPCQ questionnaire to assess pain management in selected types of cancer. *Ann Agric Environ Med*. Dec 23 2016;23(4):677-682. doi:10.5604/12321966.1220212

36. Craig AD. A new view of pain as a homeostatic emotion. Research Support, U.S. Gov't, P.H.S.

Review. *Trends Neurosci*. Jun 2003;26(6):303-7.

37. Crawford JR, Henry JD. The positive and negative affect schedule (PANAS): construct validity, measurement properties and normative data in a large non-clinical sample. Validation Studies. *Br J Clin Psychol*. Sep 2004;43(Pt 3):245-65. doi:10.1348/0144665031752934

38. Edwards RR. The association of perceived discrimination with low back pain. *J Behav Med*. Oct 2008;31(5):379-89. doi:10.1007/s10865-008-9160-9

39. Health CDoP. Maternal and Infant Health Assessment (MIHA). [*https://wwwcdphcagov/Programs/CFH/DMCAH/MIHA/Pages/Questionnaireaspx*](https://wwwcdphcagov/Programs/CFH/DMCAH/MIHA/Pages/Questionnaireaspx). 2019;(2019 (unpublished, communicated by Paula Braveman, MD, PhD, Director of UCSF Center for Social Disparities in Health))

40. Fields HL. How expectations influence pain. *Pain*. Sep 2018;159 Suppl 1:S3-S10. doi:10.1097/j.pain.0000000000001272

41. Baer RA, Smith GT, Lykins E, et al. Construct validity of the five facet mindfulness questionnaire in meditating and nonmeditating samples. Research Support, Non-U.S. Gov't

Validation Studies. *Assessment*. Sep 2008;15(3):329-42. doi:10.1177/1073191107313003

42. Williams MJ, Dalgleish T, Karl A, Kuyken W. Examining the factor structures of the five facet mindfulness questionnaire and the self-compassion scale. *Psychol Assess*. Jun 2014;26(2):407-18. doi:10.1037/a0035566
