# Supplementary material for: Mind your pain: A single-arm feasibility study to assess a smartphone-based interoceptive attention training for patients with chronic low back pain
Source: PLoS One. 2024 Oct 24;19(10):e0307690. doi: 10.1371/journal.pone.0307690 (PMC11500934; doi:10.1371/journal.pone.0307690)
Supplement: S1 Table — (DOCX) [file pone.0307690.s004.docx]

**S1 Table**:

**Table S1:** **Pre-post changes in pain measures and psychological variables (unabbreviated version of Table 2 in manuscript)**

PROMIS scores are normalized to T-scores on a 0-100 scale.­­­­­­­­­­­­­­­ BL: baseline; FU: follow-up; CI: Confidence Interval; #: Number of....

Both paired t tests and Wilcoxon Signed Rank tests comparing psychometric scale values from before to after the intervention (for variables listed in column A). This Wilcoxon test of the equality of the distributions is a nonparametric analogue of a paired t test. Data are from the Mind Your Pain study intervention. The table below includes data from n=29 participants. Means and standard deviations (SD) at each time are shown along with a mean difference and its 95% confidence interval. The paired t test p-value is shown, alongside the Wilcoxon Signed Rank test p-value. The number of participants with pre/post data who contributed to each test is shown in column L. As a check on the data, the number of unique responses at baseline (BL) and at follow up (FU) are shown in columns M & N. Paired t test p-values are shown in column I for comparison.

| **Variable Description** | **Mean at BL** | **SD (BL)** | **Mean at FU** | **SD (FU** | **Mean diff (FU-BL)** | **----95% CI----** | | **Paired t test p** | **Wilcoxon test p** | **# pre/ post data** | **# response levels at BL** | | **# response levels at FU** | |  | |
| --- | --- | --- | --- | --- | --- | --- | --- | --- | --- | --- | --- | --- | --- | --- | --- | --- |
| Low-back pain intensity, last month [0-10] | 4.79 | 1.72 | 3.14 | 1.94 | -1.66 | -2.23 | -1.08 | <0.0001 | <0.0001 | 29 | 8 | | 7 | |  | |
| PEG Score [0-30] | 13.69 | 6.22 | 8.41 | 6.57 | -5.28 | -7.09 | -3.46 | <0.0001 | <0.0001 | 29 | 17 | 17 | |  | |  |
| Pain Impact Score [8-50] | 22.34 | 8.66 | 19.69 | 8.11 | -2.66 | -4.38 | -0.93 | 0.0038 | 0.0010 | 29 | 20 | 18 | |  | |  |
| PROMIS Pain Interference 4a (T) | 57.61 | 8.11 | 55.40 | 6.36 | -2.22 | -4.74 | 0.31 | 0.083 | 0.011 | 29 | 13 | 12 | |  |  |  |
| PROMIS Physical function 6b (T) | 43.64 | 7.28 | 44.43 | 7.47 | 0.79 | -0.39 | 1.98 | 0.18 | 0.34 | 29 | 15 | 14 | |  |  |  |
| PROMIS Sleep Disturbance 6a (T) | 54.02 | 10.19 | 52.15 | 9.40 | -1.86 | -4.16 | 0.43 | 0.11 | 0.17 | 28 | 17 | 18 | |  |  |  |
| Pain Catastrophizing Total Score [0-24] | 8.34 | 5.72 | 6.83 | 5.88 | -1.52 | -3.16 | 0.13 | 0.069 | 0.15 | 29 | 13 | 14 | |  | |  |
| Pain Catastrophizing Rumination [0-8] | 3.00 | 2.07 | 2.17 | 2.04 | -0.83 | -1.50 | -0.15 | 0.018 | 0.018 | 29 | 8 | 7 | |  | |  |
| Pain Catastrophizing Magnification [0-8] | 2.62 | 2.04 | 2.14 | 2.07 | -0.48 | -1.11 | 0.15 | 0.13 | 0.17 | 29 | 8 | 8 | |  |  |  |
| Pain Catastrophizing Helplessness [0-8] | 2.72 | 2.20 | 2.52 | 2.21 | -0.21 | -0.83 | 0.41 | 0.50 | 0.73 | 29 | 8 | 8 | |  |  |  |
| PROMIS Anxiety 4a (T) | 52.87 | 9.61 | 50.68 | 10.10 | -2.19 | -5.29 | 0.90 | 0.16 | 0.19 | 28 | 11 | 9 | |  |  |  |
| PROMIS Depression 4a (T) | 51.02 | 8.52 | 49.43 | 10.34 | -1.59 | -4.36 | 1.18 | 0.25 | 0.32 | 29 | 10 | 10 | |  |  |  |
| PROMIS Fatigue 4a (T) | 53.45 | 10.02 | 53.88 | 11.24 | 0.43 | -2.84 | 3.70 | 0.79 | 0.72 | 28 | 13 | 12 | |  |  |  |
| PROMIS Social Role Ability 4a (T) | 47.20 | 6.61 | 51.13 | 8.82 | 3.92 | 1.28 | 6.57 | 0.0051 | 0.0050 | 28 | 12 | 8 | |  |  |  |
| Neuropathic Pain (Pain Detect Q) [0-35] | 7.11 | 6.55 | 7.39 | 6.02 | 0.29 | -1.08 | 1.65 | 0.67 | 0.36 | 28 | 13 | 13 | |  | |  |
| FABQ-PA Score [0-30] | 15.21 | 6.77 | 14.00 | 7.22 | -1.21 | -3.50 | 1.07 | 0.29 | 0.32 | 28 | 18 | 19 | |  | |  |
| Chronic Pain Acceptance total Score [0-48] | 28.59 | 5.17 | 28.66 | 5.86 | 0.07 | -2.04 | 2.18 | 0.95 | 0.81 | 29 | 17 | 17 | |  | |  |
| CPAQ Pain Willingness Score [0-24] | 12.79 | 4.30 | 13.21 | 5.36 | 0.41 | -1.05 | 1.88 | 0.57 | 0.79 | 29 | 12 | 18 | |  |  |  |
| CPAQ Activity Engagement Score [0-24] | 17.38 | 4.31 | 17.86 | 3.95 | 0.48 | -0.66 | 1.62 | 0.39 | 0.28 | 29 | 13 | 14 | |  |  |  |
| Perceived Stress Scale Score [0-16] | 8.50 | 1.35 | 8.61 | 1.20 | 0.11 | -0.54 | 0.75 | 0.74 | 0.68 | 28 | 7 | 6 | |  | |  |
| Intl PANAS Negative Score [0-25] | 10.32 | 3.72 | 10.54 | 3.38 | 0.21 | -0.73 | 1.16 | 0.65 | 0.56 | 28 | 12 | 12 | |  |  |  |
| Intl PANAS Positive Score [0-25] | 17.46 | 3.91 | 17.86 | 3.27 | 0.39 | -0.85 | 1.63 | 0.52 | 0.62 | 28 | 15 | 11 | |  |  |  |
| PROMIS Emotional Support 4a (T) | 53.01 | 8.74 | 52.35 | 9.83 | -0.67 | -2.62 | 1.28 | 0.49 | 0.68 | 28 | 9 | 8 | |  |  |  |
| Pain Anxiety Escape Avoidance [0-25] | 9.56 | 5.75 | 8.81 | 5.62 | -0.74 | -2.10 | 0.62 | 0.27 | 0.19 | 27 | 14 | 17 | |  |  |  |
| Pain Anxiety Physiological Anxiety [0-25] | 4.43 | 5.93 | 5.71 | 6.73 | 1.29 | -0.09 | 2.67 | 0.066 | 0.060 | 28 | 12 | 13 | |  |  |  |
| Pain Self-Efficacy [0-24] | 17.85 | 4.88 | 18.74 | 5.23 | 0.89 | -0.44 | 2.22 | 0.18 | 0.16 | 27 | 13 | 13 | |  | |  |
| Widespread pain [0-7] | 2.00 | 1.73 | 1.66 | 1.61 | -0.34 | -0.73 | 0.04 | 0.077 | 0.078 | 29 | 7 | 7 | |  | |  |
| FFMQ Acting with Awareness Score [5-25] | 17.36 | 4.43 | 16.54 | 4.10 | -0.82 | -1.90 | 0.26 | 0.13 | 0.11 | 28 | 14 | 15 | |  | |  |
| FFMQ Describing Score [5-25] | 18.00 | 3.88 | 17.50 | 3.16 | -0.50 | -1.67 | 0.67 | 0.39 | 0.59 | 28 | 14 | 10 | |  | |  |
| FFMQ: Non-Judging Score [5-25] | 18.14 | 3.41 | 16.71 | 3.96 | -1.43 | -2.45 | -0.41 | 0.01 | 0.01 | 28 | 11 | 12 | |  | |  |
| FFMQ: Non-Reactivity Score [5-25] | 15.75 | 3.83 | 15.93 | 4.07 | 0.18 | -1.04 | 1.40 | 0.77 | 0.57 | 28 | 12 | 11 | |  | |  |
| FFMQ: Observing Score [4-20] | 14.18 | 3.41 | 14.79 | 3.27 | 0.61 | -0.34 | 1.56 | 0.20 | 0.19 | 28 | 13 | 12 | |  | |  |
| FFMQ: Total Score [24-120] | 83.43 | 12.62 | 81.46 | 11.29 | -1.96 | -5.15 | 1.22 | 0.22 | 0.32 | 28 | 22 | 21 | |  | |  |
| MAIA Noticing Score [0-5] | 3.28 | 0.73 | 3.04 | 1.07 | -0.23 | -0.62 | 0.16 | 0.23 | 0.30 | 29 | 11 | 12 | |  | |  |
| MAIA Not Distracted Score [0-5] | 1.87 | 0.71 | 2.05 | 0.88 | 0.18 | -0.11 | 0.48 | 0.21 | 0.31 | 29 | 13 | 18 | |  | |  |
| MAIA Not Worrying Score [0-5] | 2.81 | 1.06 | 3.19 | 1.10 | 0.39 | 0.10 | 0.67 | 0.0091 | 0.013 | 29 | 16 | 17 | |  | |  |
| MAIA Attention Regulation Score [0-5] | 3.03 | 0.92 | 3.00 | 0.93 | -0.03 | -0.41 | 0.34 | 0.85 | 0.98 | 29 | 23 | 14 | |  | |  |
| MAIA Emotional Awareness Score [0-5] | 2.90 | 1.13 | 2.67 | 1.29 | -0.23 | -0.65 | 0.18 | 0.25 | 0.26 | 29 | 18 | 15 | |  | |  |
| MAIA Self-Regulation Score [0-5] | 2.77 | 0.86 | 2.77 | 0.85 | 0.00 | -0.42 | 0.42 | 1.00 | 0.70 | 29 | 12 | 11 | |  | |  |
| MAIA Body Listening Score [0-5] | 1.90 | 1.04 | 1.97 | 1.16 | 0.07 | -0.27 | 0.41 | 0.68 | 0.89 | 29 | 11 | 13 | |  | |  |
| MAIA Trusting Score [0-5] | 3.28 | 1.19 | 2.92 | 1.37 | -0.36 | -0.87 | 0.16 | 0.17 | 0.26 | 29 | 11 | 12 | |  | |  |
| MAIA Total Score [0-5] | 2.73 | 0.48 | 2.70 | 0.61 | -0.03 | -0.27 | 0.22 | 0.82 | 0.99 | 29 | 28 | 29 | |  | |  |

**Description of the applied questionnaires:**

Low-back pain intensity, last month: Numeric Rating Scale 0 to 10, high values indicating higher intensity, the standard single-item measure for pain intensity. (1, 2) We used the scale for a 1-month pain recall.

PEG Scale (3): assessing pain intensity and interference (**P**ain, **E**njoyment, **G**eneral Activity), three items each on 0-10 scale to create a summary score between 0 and 30 “in the past week”. PEG-1 is for pain intensity, PEG-2 for pain interference with enjoyment in life, PEG-3 for pain interference with general activity. Cronbach’s alpha in our sample was 0.94.

Pain Impact (4): Pain Impact is a 9-item composite measure derived from PROMIS-29 and was suggested by the NIH Chronic Low Back Pain Task Force for any research on chronic low back pain. It includes pain intensity, pain impact, and physical function. Scores range from 8-50. Cronbach’s alpha in our sample was 0.84.

Pain Self-Efficacy Questionnaire (PSEQ-4)(5): *is a* short versions of the 10-item PSEQ: items 4, 6, 8, and 9. Both versions are adequately responsive instruments in patients with CLBP.(5) Scores range from 0-24. Cronbach’s alpha in our sample was 0.94. PSEQ is an independent predictor of poor recovery after initial consultation for LBP and is described as the attitudes and beliefs that people with chronic pain hold to carry out certain daily activities, even in the presence of pain. *Methods:*

Pain DETECT Questionnaire (PD-Q) (6): The PD-Q was developed and validated in co-operation with the German Research Network on Neuropathic Pain to detect neuropathic pain. 7 items for neuropathic pain quality descriptors on a 6-point scale, scored by multiplying the answer scores by the numbers of items scored that way produced a score range from 0-35. It has shown reliability and validity in patients with cLBP and numerous other conditions and is used worldwide.(7)

Widespread Pain [0-7] is a proxy measure for the intensity or severity of ‘neuroplastic’ pain and central sensitization, such as for fibromyalgia. It simply sums up the number of up to seven pain sites in the body from 0-7. It is an abbreviated version of the Widespread Pain Index WPI(8)

PROMIS stands for Patient-Reported Outcomes Measurement Information System and was developed by the NIH. (<http://www.healthmeasures.net/index.php?option=com_instruments&view=measure&id=1050&Itemid=992>). Scores are based on the T-score metric that standardizes score values around the mean of a normative population sample. Scores can range from 0-100 with 50 indicating the population mean. We used the T-scores for PROMIS Pain Interference 4a, PROMIS Physical Function 6b, PROMIS Anxiety 4a, PROMIS Depression 4a, PROMIS Social Role Ability 4a, PROMIS Sleep Disturbance 6a, PROMIS Fatigue 4a.

Pain Catastrophizing Scale PCS-6 [0-24] (9): The PCS is widely used internationally as a key parameter for changes in catastrophizing that have been reported as mediating therapeutic improvements for cLBP. The PCS has 13 items for 3 subscales with Cronbach/s α for the total PCS .92., and 85, .75, and .86, respectively, for Rumination, Magnification, and Helplessness.(10) Items 4, 5, 6, 10, 11, 13 create the short form for the 3 subscales, which correlates at .95 with the original scale.(9) Alpha was .88 in prior studies.(9)

Fear-Avoidance Beliefs Questionnaire Physical Activity FABQ-PA(11): The 5-item Physical Activity (PA) scale is much shorter than the TAMPA Kinesiophobia scale, defined as “an irrational, and debilitating fear of physical movement and activity resulting from a feeling of vulnerability to painful injury or re-injury”. (Kori et al., 1990). A second FABQ-Work Activity (WA) scale is only for working population. It consists of 5 items on a 7-point Likert scale inquiring about beliefs regarding pain and is widely used internationally with a score range from 0-30. It reflects the belief that activity may result in (re)injury or increased pain. Fear of movement or of re-injury has been found to one of the main predictors of persistence of pain. Reliability, validity and sensitivity to change are well supported.(12)

Chronic Pain Acceptance Questionnaire CPAQ-8 (13): The CPAQ-8 has 8 items with a summary score from 0-48 and two factors: Activity Engagement and Pain Willingness. Acceptance of chronic pain has been shown to be associated with the trajectory of chronic pain; it is modifiable by MBSR.(14) It is a precondition for the capacity to develop mindful interoceptive awareness of pain. CPAQ-8 demonstrated good psychometric properties and sensitivity to rehabilitation changes.(15) Cronbach’s alpha in our sample was 0.80.

Perceived Stress Scale PSS-4 (16): 4 items ask about feelings and thoughts in the past month on a 5-point Likert scale. It is the shortest version of the PSS that has shown excellent validity and reliability in multiple versions. Perceived stress has shown to be associated pain perception. The PSS is applied worldwide with numerous clinical conditions that vary according to stress levels. Cronbach’s alpha in our sample was 0.77.

Positive and Negative Affect Schedule (PANAS)(17) SF: *5* items for frequency of each positive or negative emotions are assessed on a 5-point Likert scales. Positive and negative affect are separate scales each scoring between 0 and 25. Emotion are strongly correlated to pain perception as the brain regions for emotions and pain overlap. Chronic pain has been proposed to be viewed as a homeostatic emotion.(18) The PANAS is a reliable and valid measure of the constructs it was intended to assess, although the hypothesis of complete independence between positive and negative affect was rejected.(19) iPANAS SF is an internationally validated version.(20)

Pain Anxiety Symptoms Scale - Short Form (PASS-20)(21): we used two of the four subscales, items 6-10 for Avoidance and 16-20 for Physiological Pain Anxiety; the other subscales are already covered with anxiety and catastrophizing. There is a significant relation between fear and avoidance of pain and the suffering and disability of chronic pain. Cronbach’s alphas in our sample were 0.83 and 0.94, respectively.

Five-Facet Mindfulness Questionnaire FFMQ (22): The FFMQ measures dispositional mindfulness and, despite reported shortcomings,(23) is among the most widely used mindfulness self-report measure. The FFMQ has 39 items for 5 scales with *α* between .77 and .92.(23) The factor structure has been confirmed in non-meditators(23). Total summary scores range from 24 to 120. The five scales assess Acting with Awareness [5-25], Describing [5-25], Non-Judging [5-25], Non-Reactivity [5-25], and Observing [4-20].

Multi-dimensional Assessment of Interoceptive Awareness – Version 2 (MAIA-2)(24): The MAIA has become a standard self-report measure for interoceptive awareness or sensibility, purportedly a key mechanism of action for mind-body interventions(25), and its 8 scales have shown to be strong predictors of treatment response for cLBP.(26) *W*e used all scales Noticing, Not-Distracting, Not-Worrying, Attention Regulation, Emotional Awareness, Self-Regulation, Body Listening, and Trusting, all answered on a 6-point Likert scale with acceptable Cronbach *α* values, excellent sensitivity to change and validity.(24) Cronbach’s alpha for the entire scale in our sample was 0.91.

Financial Strain was assessed by a single item used/developed in(27)**:** “How hard is it for you (and your family) to pay for the very basics like food, medical care, and heating?” Response options: 1 = very hard, 2 = hard, 3 = somewhat hard, 4 = not very hard, and 5 = do not know; recoded into a binary variable, in which very hard and hard were considered high financial strain, and somewhat hard and not very hard were considered low financial strain.

Primary Care PTSD screening tool(28), uses a single screening question, that if answered positively, branches out to 5 sub-items: “Sometimes things happen to people that are unusually or especially frightening, horrible, or traumatic. For example: a serious accident or fire, a physical or sexual assault or abuse, an earthquake or flood, a war, seeing someone be killed or seriously injured, having a loved one die through homicide or suicide. Have you ever experienced this kind of event? No/Yes A) as a child? B) as an adult?” If Yes 4 PTSD symptom items are asked. The addition asking for “as child or adult” is a modification.

Perceived Discrimination.(29, 30) Perceived discrimination may be a stronger predictors than physical health variables in minorities. We used three modified items used in a large statewide survey by the California Department of Public Health in its recent 2019 Maternal and Infants Health Assessment (MIHA). The items assess perceived experience of discrimination and worriesThe modification is that we added discrimination for sexual orientation. The items are 1) Overall during your life until now, how often *have you worried* that you might be treated or judged unfairly because of your race, ethnic group or social discrimination? 2) Overall during your life until now, how often *have you worried* that a loved one like your spouse, partner, child, or parent might be treated or judged unfairly because of their race, ethnic group or sexual orientation?  3) Overall during your life until now, how often *have you been* discriminated against, prevented from doing something, or hassled or made to feel inferior because of your race, ethnicity, color or sexual orientation? Ordinal scale from 0 = ‘never’ to 3 = ‘very often’.

**References:**

1. Holdgate A, Asha S, Craig J, Thompson J. Comparison of a verbal numeric rating scale with the visual analogue scale for the measurement of acute pain. Emerg Med (Fremantle). 2003;15(5-6):441-6.

2. Krebs EE, Carey TS, Weinberger M. Accuracy of the pain numeric rating scale as a screening test in primary care. Journal of general internal medicine. 2007;22(10):1453-8.

3. Krebs EE, Lorenz KA, Bair MJ, Damush TM, Wu J, Sutherland JM, et al. Development and initial validation of the PEG, a three-item scale assessing pain intensity and interference. Journal of general internal medicine. 2009;24(6):733-8.

4. Deyo RA, Dworkin SF, Amtmann D, Andersson G, Borenstein D, Carragee E, et al. Report of the NIH task force on research standards for chronic low back pain. Spine. 2014;39(14):1128-43.

5. Chiarotto A, Vanti C, Cedraschi C, Ferrari S, de Lima ESRF, Ostelo RW, Pillastrini P. Responsiveness and Minimal Important Change of the Pain Self-Efficacy Questionnaire and Short Forms in Patients With Chronic Low Back Pain. The journal of pain : official journal of the American Pain Society. 2016;17(6):707-18.

6. Freynhagen R, Baron R, Gockel U, Tolle TR. painDETECT: a new screening questionnaire to identify neuropathic components in patients with back pain. Current medical research and opinion. 2006;22(10):1911-20.

7. Cappelleri JC, Koduru V, Bienen EJ, Sadosky A. A cross-sectional study examining the psychometric properties of the painDETECT measure in neuropathic pain. Journal of pain research. 2015;8:159-67.

8. Wolfe F, Clauw DJ, Fitzcharles MA, Goldenberg DL, Katz RS, Mease P, et al. The American College of Rheumatology preliminary diagnostic criteria for fibromyalgia and measurement of symptom severity. Arthritis Care Res (Hoboken). 2010;62(5):600-10.

9. McWilliams LA, Kowal J, Wilson KG. Development and evaluation of short forms of the Pain Catastrophizing Scale and the Pain Self-efficacy Questionnaire. European journal of pain. 2015;19(9):1342-9.

10. Sullivan MJL, Bishop SR, Pivik J. The Pain Catastrophizing Scale: Development and validation. Psychological assessment. 1995;7(4):524-32.

11. Waddell G, Newton M, Henderson I, Somerville D, Main CJ. A Fear-Avoidance Beliefs Questionnaire (FABQ) and the role of fear-avoidance beliefs in chronic low back pain and disability. Pain. 1993;52(2):157-68.

12. George SZ, Calley D, Valencia C, Beneciuk JM. Clinical Investigation of Pain-related Fear and Pain Catastrophizing for Patients With Low Back Pain. The Clinical journal of pain. 2011;27(2):108-15.

13. Fish RA, McGuire B, Hogan M, Morrison TG, Stewart I. Validation of the chronic pain acceptance questionnaire (CPAQ) in an Internet sample and development and preliminary validation of the CPAQ-8. Pain. 2010;149(3):435-43.

14. la Cour P, Petersen M. Effects of mindfulness meditation on chronic pain: a randomized controlled trial. Pain medicine. 2015;16(4):641-52.

15. Rovner GS, Arestedt K, Gerdle B, Borsbo B, McCracken LM. Psychometric properties of the 8-item Chronic Pain Acceptance Questionnaire (CPAQ-8) in a Swedish chronic pain cohort. Journal of rehabilitation medicine. 2014;46(1):73-80.

16. Cohen S, Kamarck T, Mermelstein R. A global measure of perceived stress. Journal of health and social behavior. 1983;24(4):385-96.

17. Watson D, Clark LA, Tellegen A. Development and validation of brief measures of positive and negative affect: the PANAS scales. Journal of personality and social psychology. 1988;54(6):1063-70.

18. Craig AD. A new view of pain as a homeostatic emotion. Trends in neurosciences. 2003;26(6):303-7.

19. Crawford JR, Henry JD. The positive and negative affect schedule (PANAS): construct validity, measurement properties and normative data in a large non-clinical sample. The British journal of clinical psychology. 2004;43(Pt 3):245-65.

20. Thompson ER. DEVELOPMENT AND VALIDATION OF AN INTERNATIONALLY RELIABLE SHORT-FORM OF THE POSITIVE AND NEGATIVE AFFECT SCHEDULE (PANAS). JOURNAL OF CROSS-CULTURAL PSYCHOLOGY. 2007;38(2):227-42.

21. McCracken LM, Dhingra L. A short version of the Pain Anxiety Symptoms Scale (PASS-20): preliminary development and validity. Pain research & management. 2002;7(1):45-50.

22. Baer RA, Smith GT, Lykins E, Button D, Krietemeyer J, Sauer S, et al. Construct validity of the five facet mindfulness questionnaire in meditating and nonmeditating samples. Assessment. 2008;15(3):329-42.

23. Williams MJ, Dalgleish T, Karl A, Kuyken W. Examining the factor structures of the five facet mindfulness questionnaire and the self-compassion scale. Psychological assessment. 2014;26(2):407-18.

24. Mehling WE, Acree M, Stewart A, Silas J, Jones A. The Multidimensional Assessment of Interoceptive Awareness, Version 2 (MAIA-2). PloS one. 2018;13(12):e0208034.

25. Mehling WE. Differentiating attention styles and regulatory aspects of self-reported interoceptive sensibility. Philosophical transactions of the Royal Society of London Series B, Biological sciences. 2016;371(1708).

26. Vachon-Presseau E, Berger SE, Abdullah TB, Griffith JW, Schnitzer TJ, Apkarian AV. Identification of traits and functional connectivity-based neuropsychotypes of chronic pain. bioRxiv preprint doi <http://dxdoiorg/101101/421438>. 2018.

27. Puterman E, Adler N, Matthews KA, Epel E. Financial strain and impaired fasting glucose: the moderating role of physical activity in the Coronary Artery Risk Development in Young Adults study. Psychosomatic medicine. 2012;74(2):187-92.

28. Prins A, Bovin MJ, Smolenski DJ, Marx BP, Kimerling R, Jenkins-Guarnieri MA, et al. The Primary Care PTSD Screen for DSM-5 (PC-PTSD-5): Development and Evaluation Within a Veteran Primary Care Sample. Journal of general internal medicine. 2016;31(10):1206-11.

29. Health CDoP. Maternal and Infant Health Assessment (MIHA). <https://wwwcdphcagov/Programs/CFH/DMCAH/MIHA/Pages/Questionnaireaspx>. 2019(2019 (unpublished, communicated by Paula Braveman, MD, PhD, Director of UCSF Center for Social Disparities in Health)).

30. Braveman P, Heck K, Egerter S, Dominguez TP, Rinki C, Marchi KS, Curtis M. Worry about racial discrimination: A missing piece of the puzzle of Black-White disparities in preterm birth? PloS one. 2017;12(10):e0186151.
